# Supplementary material for: Iterative Mechanism of Macrodiolide Formation in the Anticancer Compound Conglobatin
Source: Chem Biol. 2015 Jun 18;22(6):745–54. doi: 10.1016/j.chembiol.2015.05.010 (PMC4504003; doi:10.1016/j.chembiol.2015.05.010)
Supplement: Document S1. Tables S1 and S2, Figures S1–S4, and NMR Data Assignment and the Spectra [file mmc1.pdf]

Chemistry & Biology, Volume *22*

Supplemental Information

Iterative Mechanism of Macrodiolide Formation

in the Anticancer Compound Conglobatin

Yongjun Zhou, Annabel C. Murphy, Markiyan Samborskyy, Patricia Prediger, Luiz Carlos Dias, and Peter F. Leadlay

1. Tables and Figures

Table S1. Deduced ORF functions in the 41 kbp insert cloned in pYJ24 (related to Figure 3B).

Protein	Size (aa)	Proposed function	Identity/ query cover	Protein Homolog [a]
**CongA**	1210	Loading module	47%, 94%	OzmO, ABS90476.1
**CongB**	856	Module 1a	53% , 99%	OzmQ, ABS90478.1
**CongC**	4476	Module 1b, 2, and 3	47%, 95%	DEBS2, Q03132
**CongD**	2124	Module 4	48%, 87%	DEBS2, Q03132
**CongE**	375	Unknown, N-terminal ATP binding motif	80%, 99%	OzmP, WP_037634315
**R1**	195	LuxR family transcriptional regulator	46%, 98%	FscRI, AAQ82551.1
**1**	487	NADP-specific glutamate dehydrogenase	67%, 90%	P31026.2
**2**	315	Oxidoreductase	46%, 90%	P35320.1
**3**	163	HTH-type transcriptional regulator	32%, 77%	P44558.1
**4**	217	Methyltransferase	28%, 66%	B3QLI9.1
**5**	446	Unknown	no hits	
**6**	350	Unknown	no hits	
**7**	208	HTH-type transcriptional regulator	28%, 80%	P96676.1
**8**	381	L-threonine 3-dehydrogenase	90%, 89%	Q82MN2.1
**9**	400	Glycine acetyltransferase	66%, 94%	P0AB78.1
**10**	332	LysR-family transcriptional regulator	29%, 75%	P20668.3

[a] CongA-E and R1 are given GenBank No. Orf 1 to 10 are given the No. of UniProtKB/Swiss-Prot

Table S2. HR-MS Data of the Compounds Used or Generated in Cong-TE Assay (related to Figure 4).

Products	Formula	calc. m/z	det. m/z	Error (ppm)
**2**	$C_{28}H_{38}N_2O_6H^+$	499.2803	499.2791	-2.29
**4a**	$C_{14}\ H_{21}\ O_4\ N_1H^+$	268.1543	268.1533	-4.05
**4b**	$C_{18}H_{28}O_4N_2SH^+$	369.1843.	369.1829	-3.59
**5**	$C_{32}H_{47}N_3O_7SNa^+$	640.3027	640.3007	-3.08
**8**	$C_{31}H_{48}N_2O_7SH^+$	593.3255	593.3256	0.17
**9**	$C_{27}H_{41}NO_7H^+$	492.2956	492.2976	4.06
**10**	$C_{45}H_{67}N_3O_{10}SH^+$	842.4620	842.4644	2.85
**11**	$C_{42}H_{61}N_3O_9SH^+$	784.4201	784.4186	-1.99

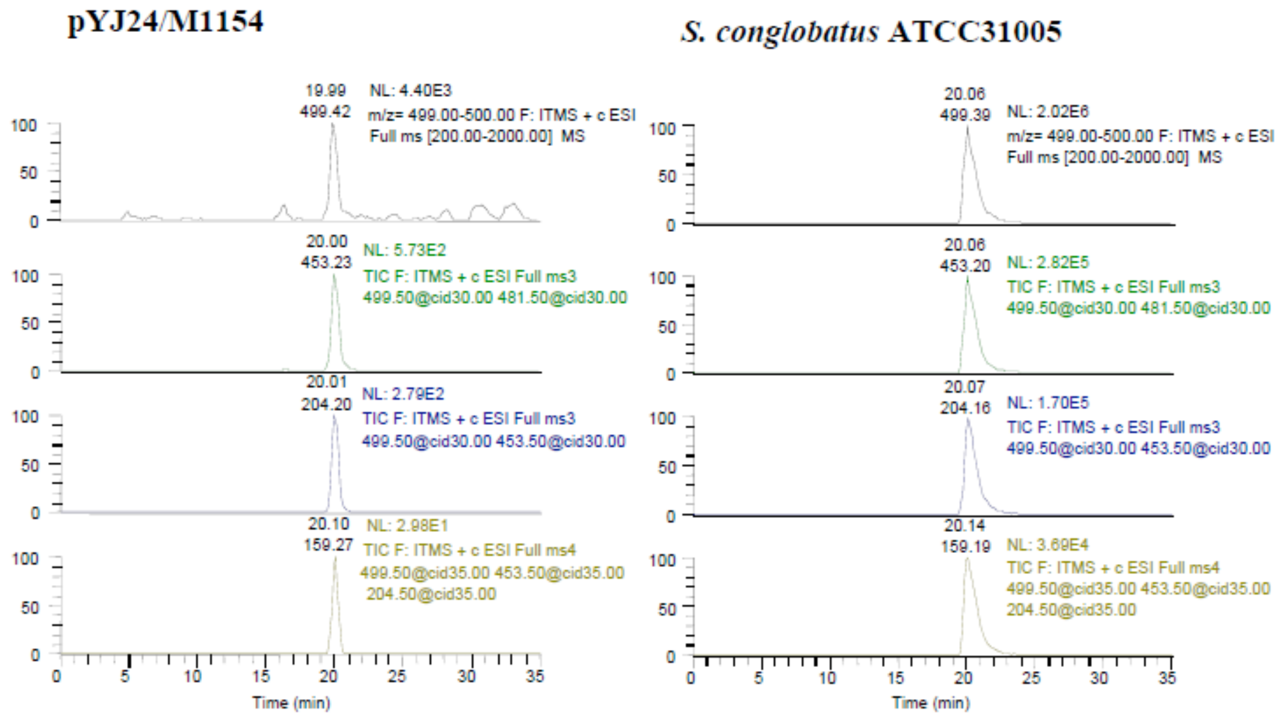

Figure S1. LC-MS/MS analysis of conglobatin heterologous expression in M1154 (related to Figure 3C).

The authentic compound produced by *S. conglobatus* ATCC31005 served as a positive control.

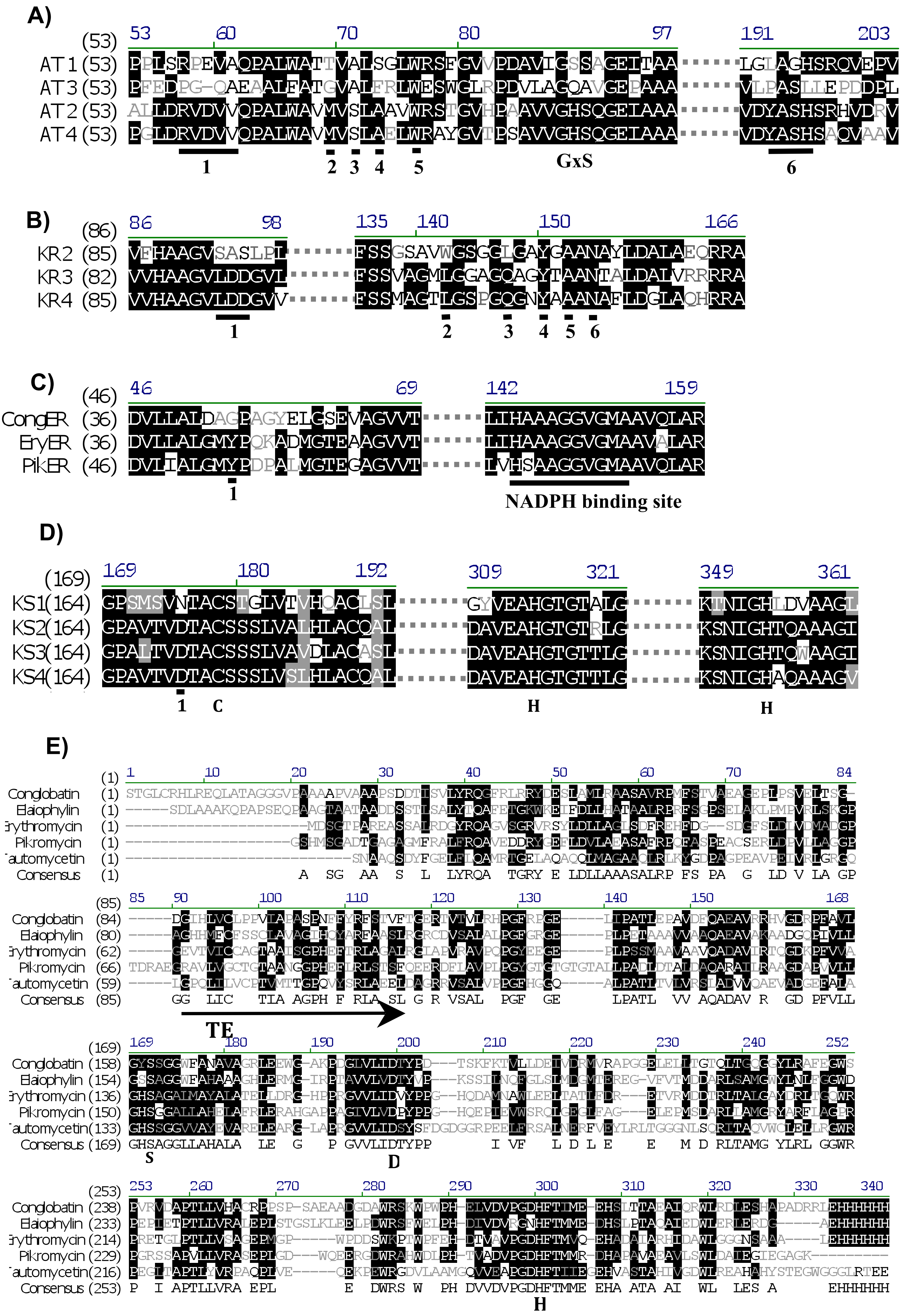
A)
AT1 (53)
AT3 (53)
AT2 (53)
AT4 (53)
1
2 3 4
5
GxS
6
B)
KR2 (85)
KR3 (82)
KR4 (85)
1
2
3 4 5 6
C)
CongER (36)
EryER (36)
PikER (46)
1
NADPH binding site
D)
KS1(164)
KS2(164)
KS3(164)
KS4(164)
1 C
H
H
E)
Conglobatin
Elaiophylin
Pikromycin
Consensus
TE
S
D
H

Figure S2. Amino Acid Sequence Alignment of AT, KR, ER, KS and TE Domains (related to Figure 2). A) Both AT2 and AT4 contain the “YASH” motif specific for loading methylmalonyl unit (Del Vecchio et al., 2003). AT3 has no Ser residue in the “GxS” motif and should be a cryptic domain. The “RVDVV” motif marked by 1 and “MxSxAxxW” motif marked by 2 to 5 are also the footprint of methylmalonyl-CoA specificity. B) KR2 should yield the configuration of *2R* and *3S* according to the residues of position 1: no LDD, 2: W, and 3: no H. KR3 and KR4 should deliver the product with the configuration of *2R* and *3R* to further reduction carried on respectively by DH3 and ER3 in module 3 and DH4 in module 4 according to the residues of position 1: LDD, 5: no P (Keatinge-Clay AT, 2007). C) ER (CongER) from module 3 should generated *2R* configuration rather than *2S* in the case of Y present at 1 position (Kwan *et al*., 2010). EryER and PikER are ER respectively derived from erythromycin and pikromycin. D) All of the four KS domains have the essential active site cysteine (C) and two conserved histidine (H) residues. KS1 differs from the other three KS domains by the alteration of residue N instead of D in position 1. E) Multiple alignment of the functional TE domains expressed in *E. coli*. Cong-TE was investigated in this work. The TEs respectively derived from erythromycin (Tsai et al., 2001), pikromycin (Giraldes et al., 2006), and tautomycetin (Scaglione *et al*., 2010) were characterized by crystal structure. The TE from eliophylin was characterized by *in vitro* experiments (Zhou et al., 2015).The start region of the TE domains and the active residues are all labeled.

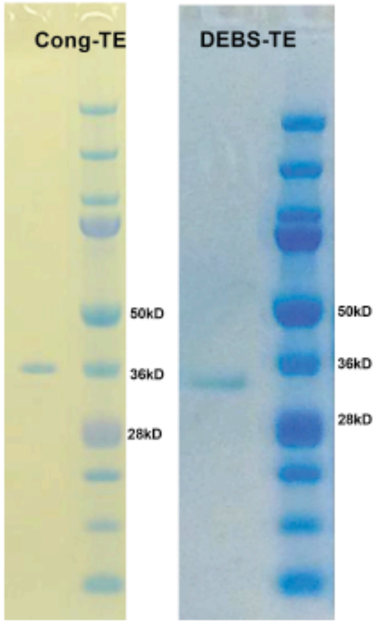

Figure S3. SDS-PAGE gel of the purified proteins (related to Figure 4 and Figure 5).

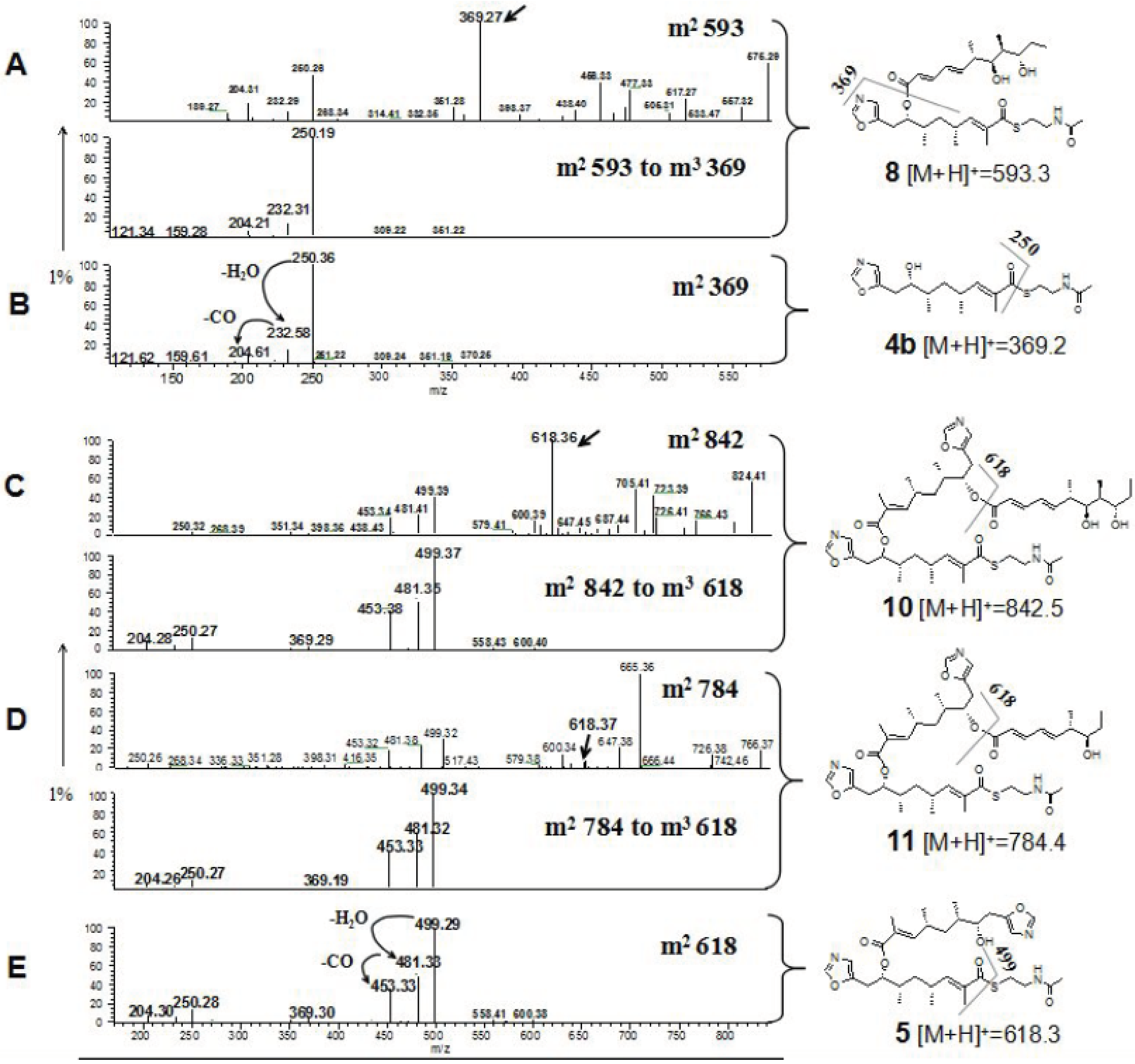

Figure S4. **MS/MS Analysis of Compounds 8, 10, and 11 (related to Figure 5).**

The MS-MS fingerprints of known compounds **4b** and **5** are elucidated respectively in sections B and E. The MS-MS of **4b**, *m/z* 369 (B) is identical to the MS-MS-MS of *m/z* 369 derived from the MS-MS of **8**, *m/z* 593 (A). The MS_2 of **5**, *m/z* 618 (E) is identical to the MS_3 of *m/z* 618 derived from the MS-MS of either **10**, *m/z* 842 (C) or **11**, *m/z* 784 (D). The predicted fragmentations are indicated in the chemical structures.

2. NMR Spectra

2. 1. Compound 2 (produced from fermentation).

^{1}H NMR

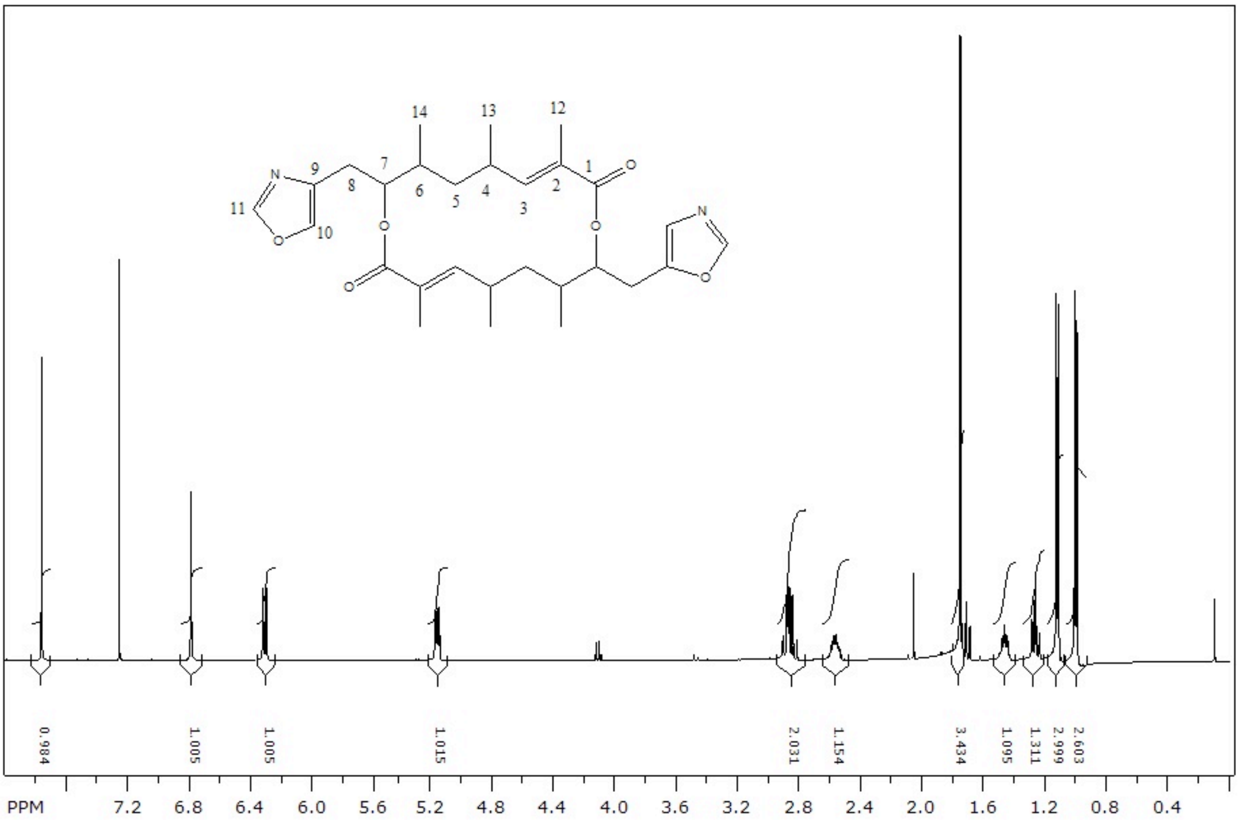

^{13}C NMR

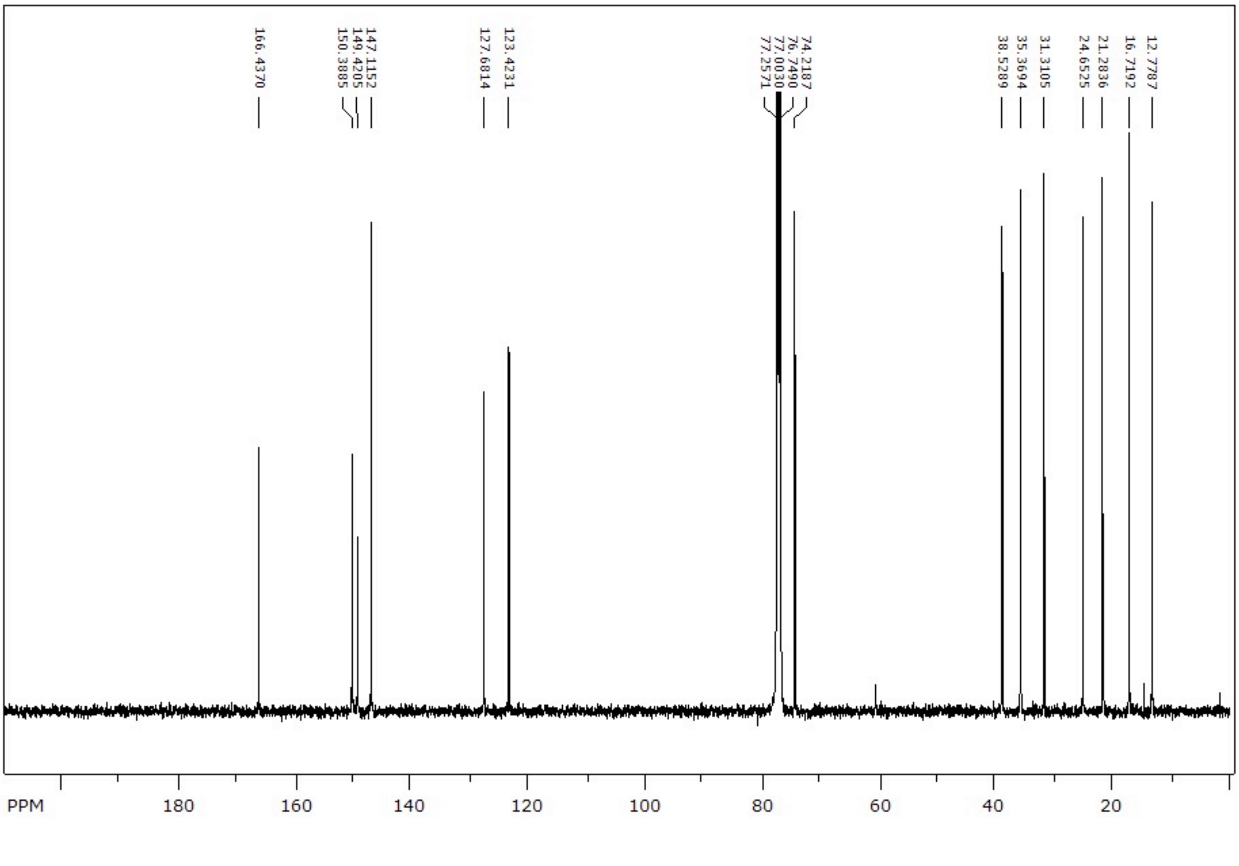

COSY NMR

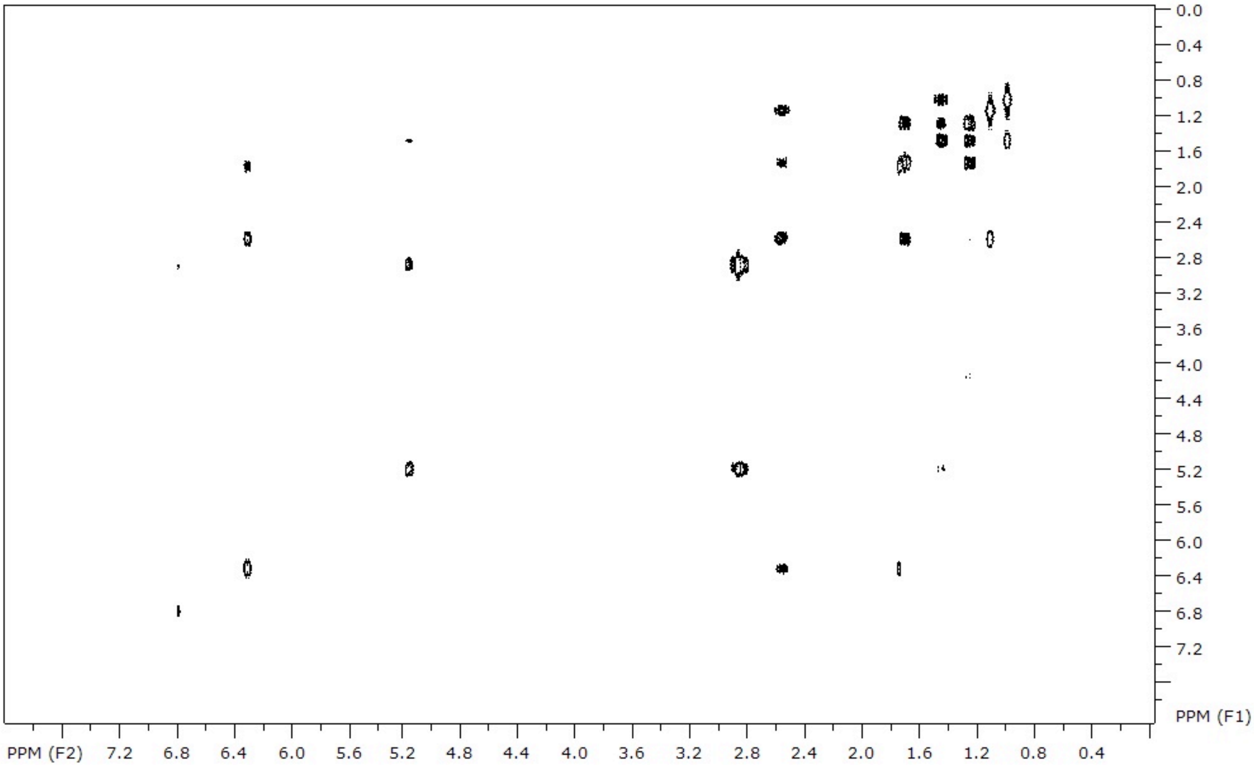

HSQC NMR

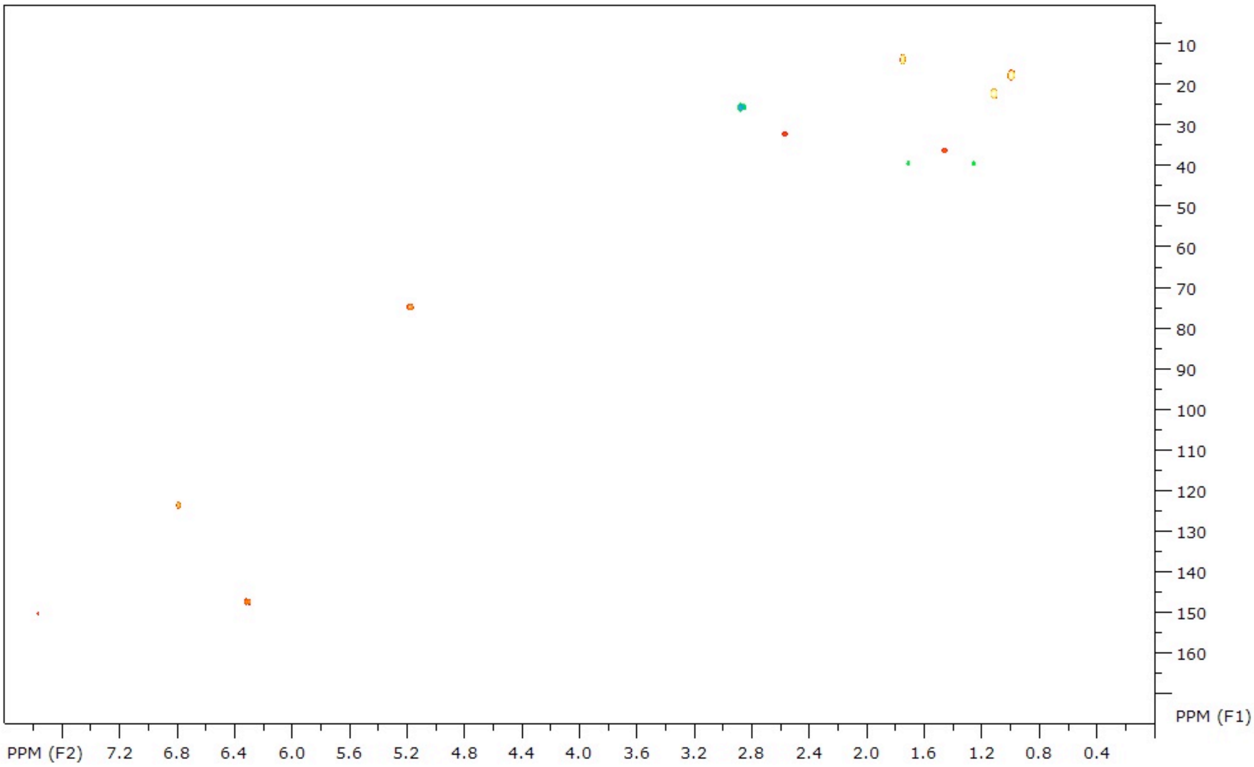

HMBC NMR

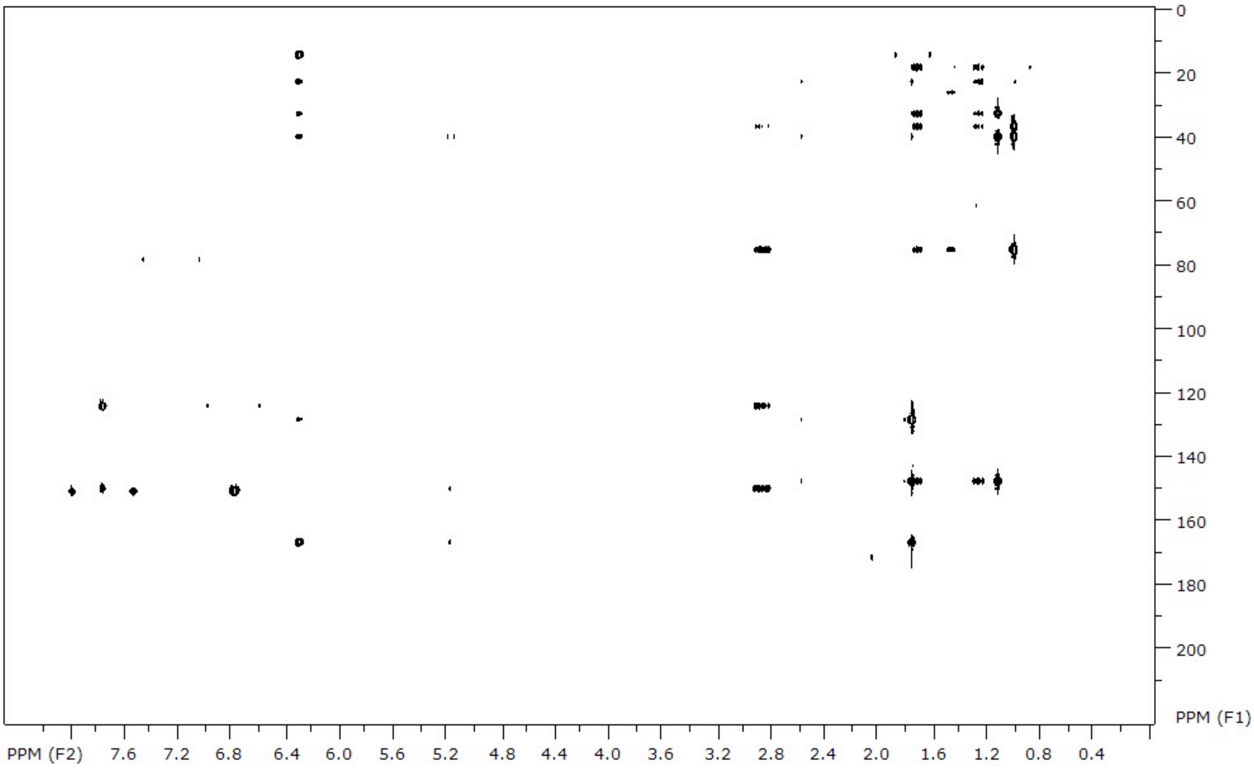

2.2. Compound 4a.

^{1}H NMR

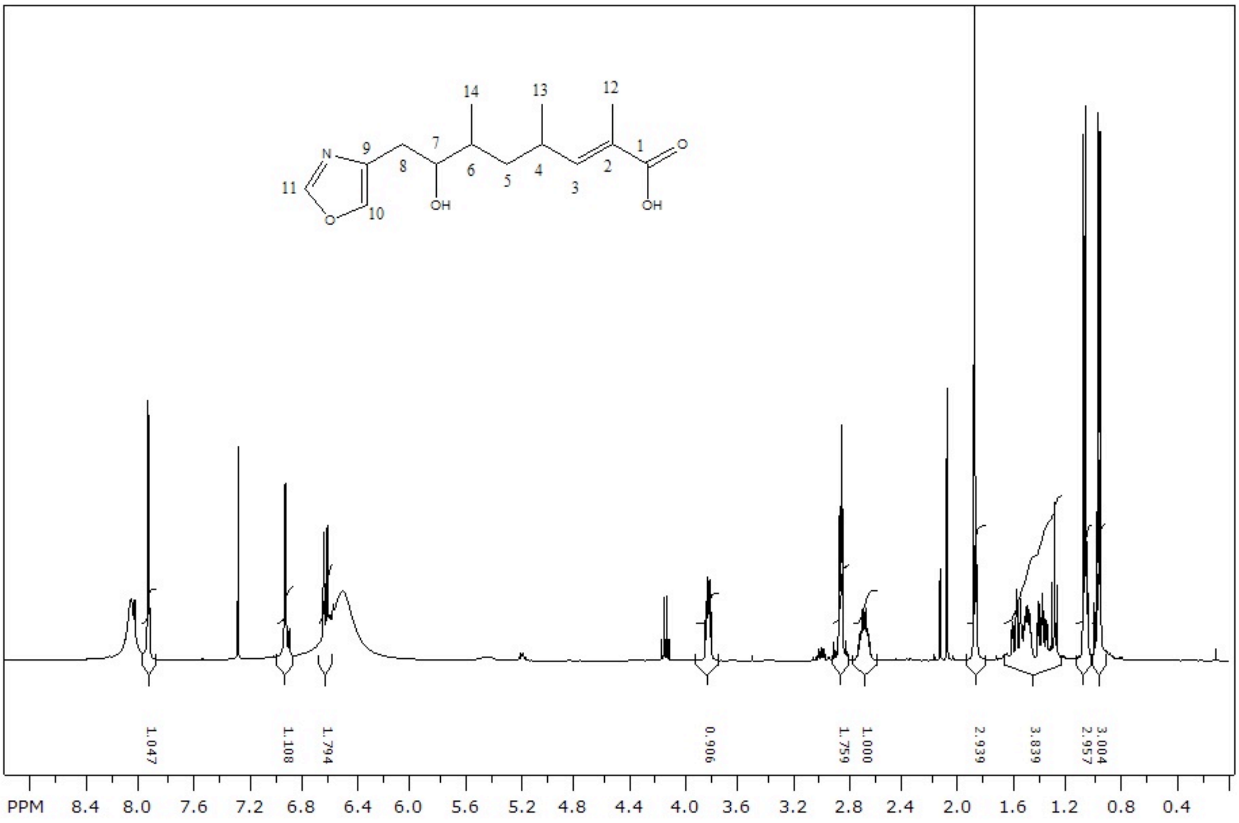

^{13}C NMR

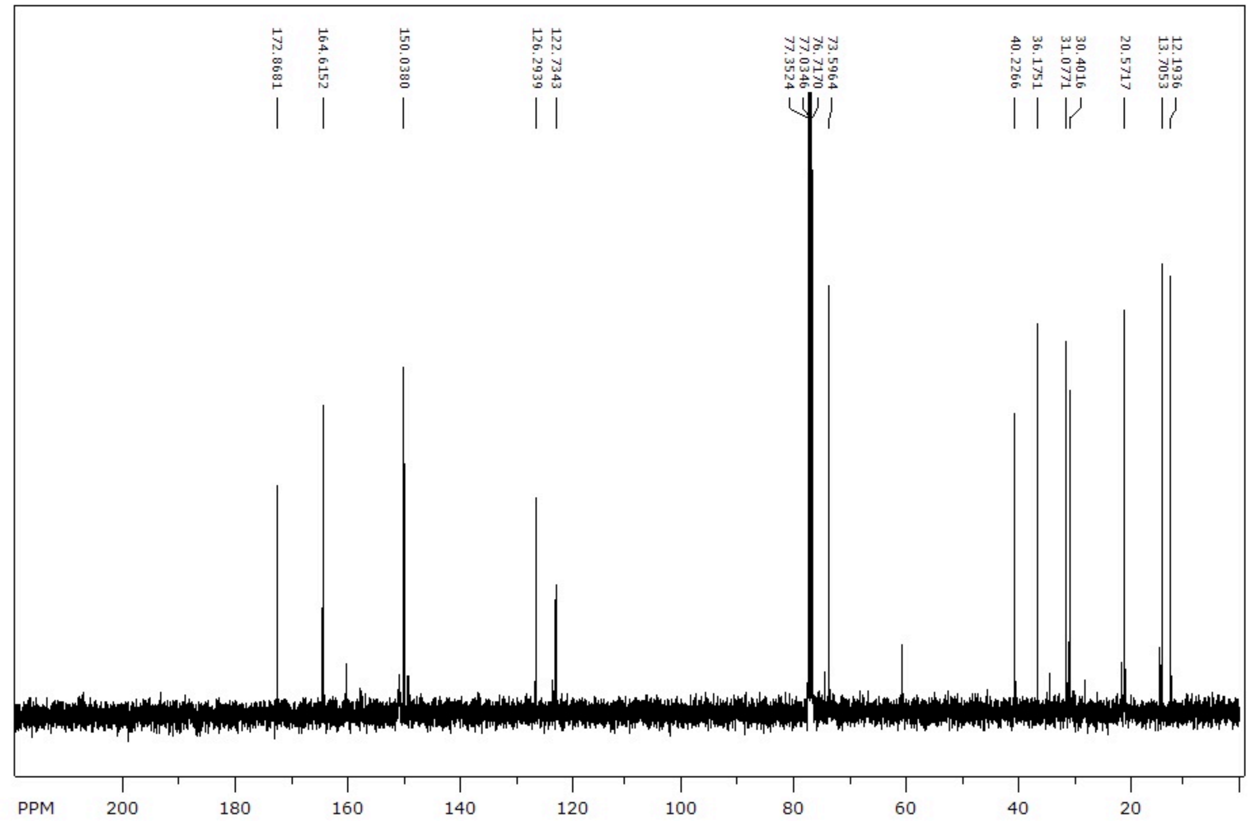

COSY NMR

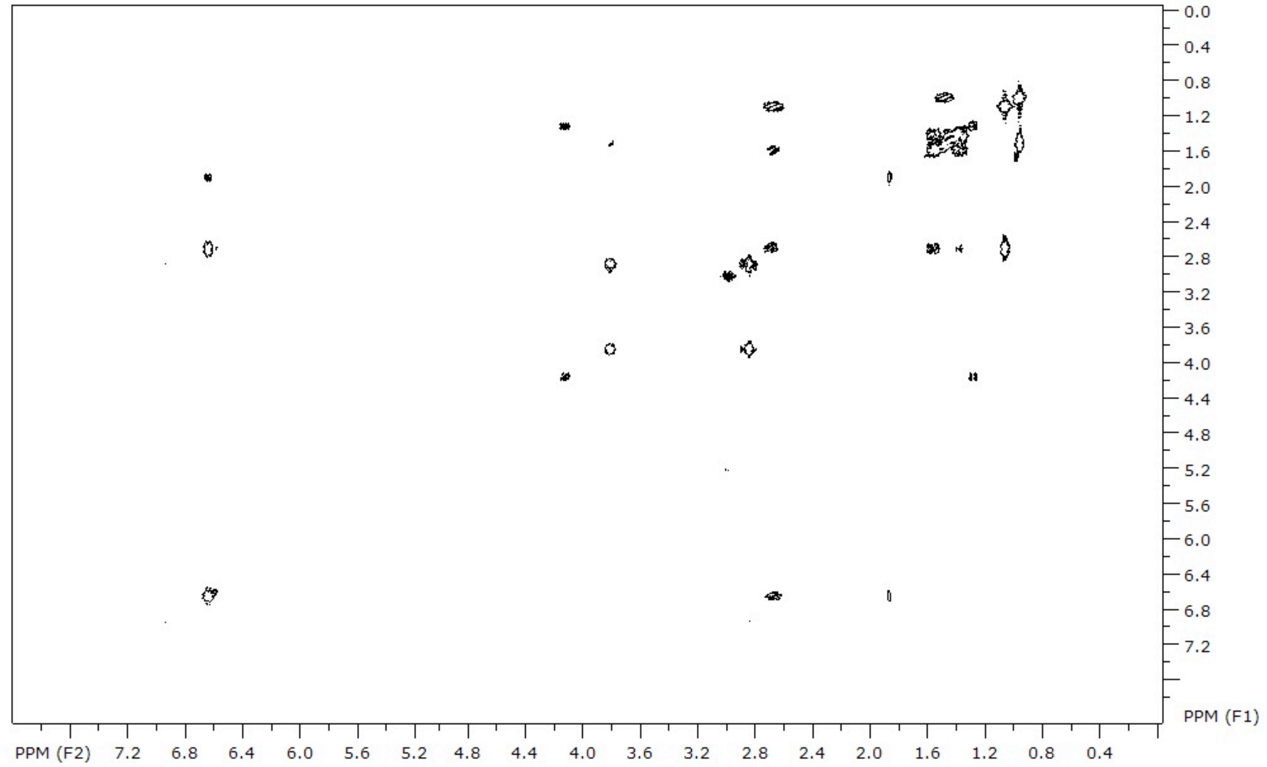

HSQC NMR

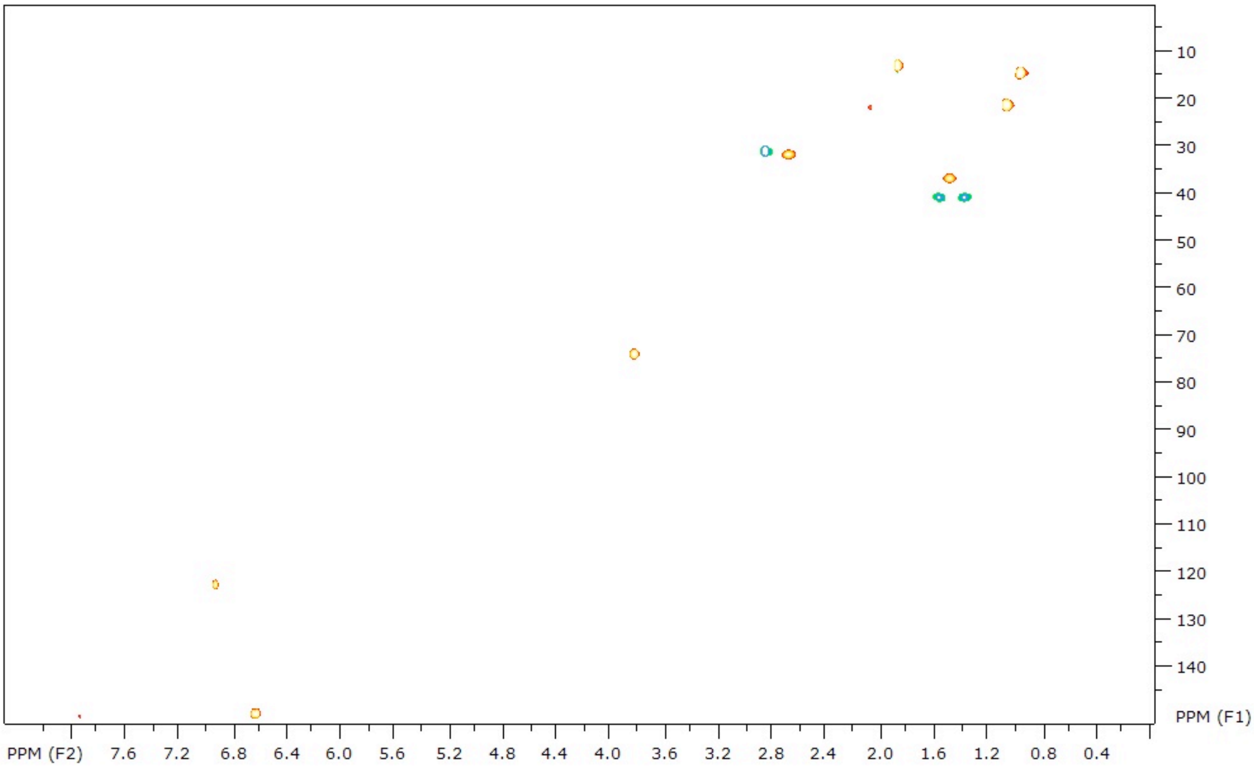

HMBC NMR

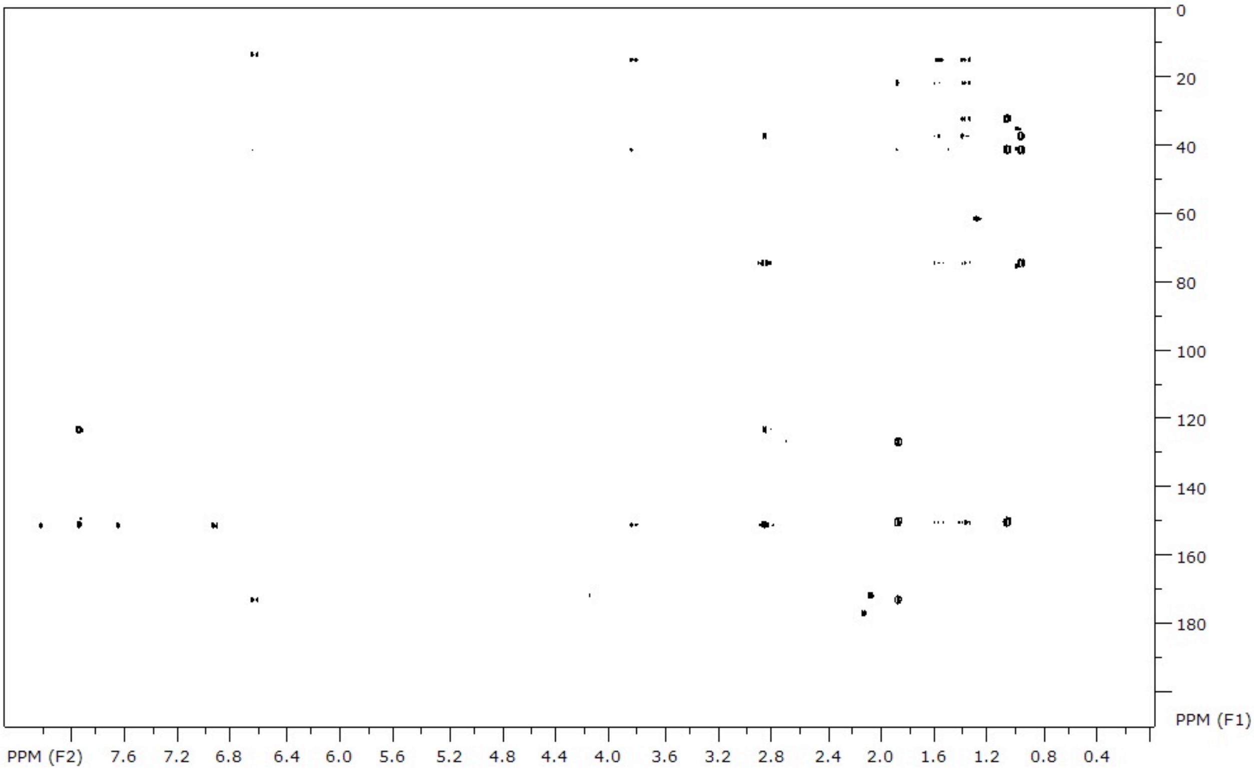

2.3. Compound 4b.

1H NMR

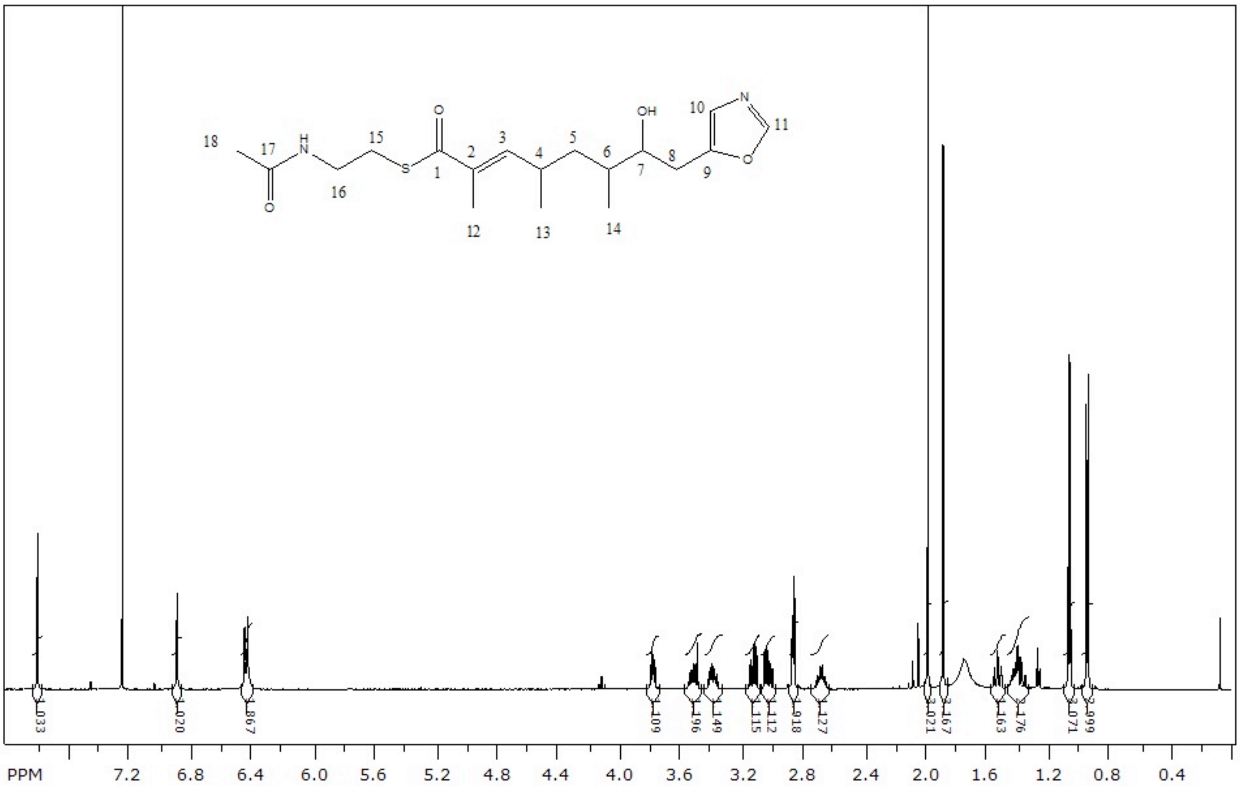

13C NMR

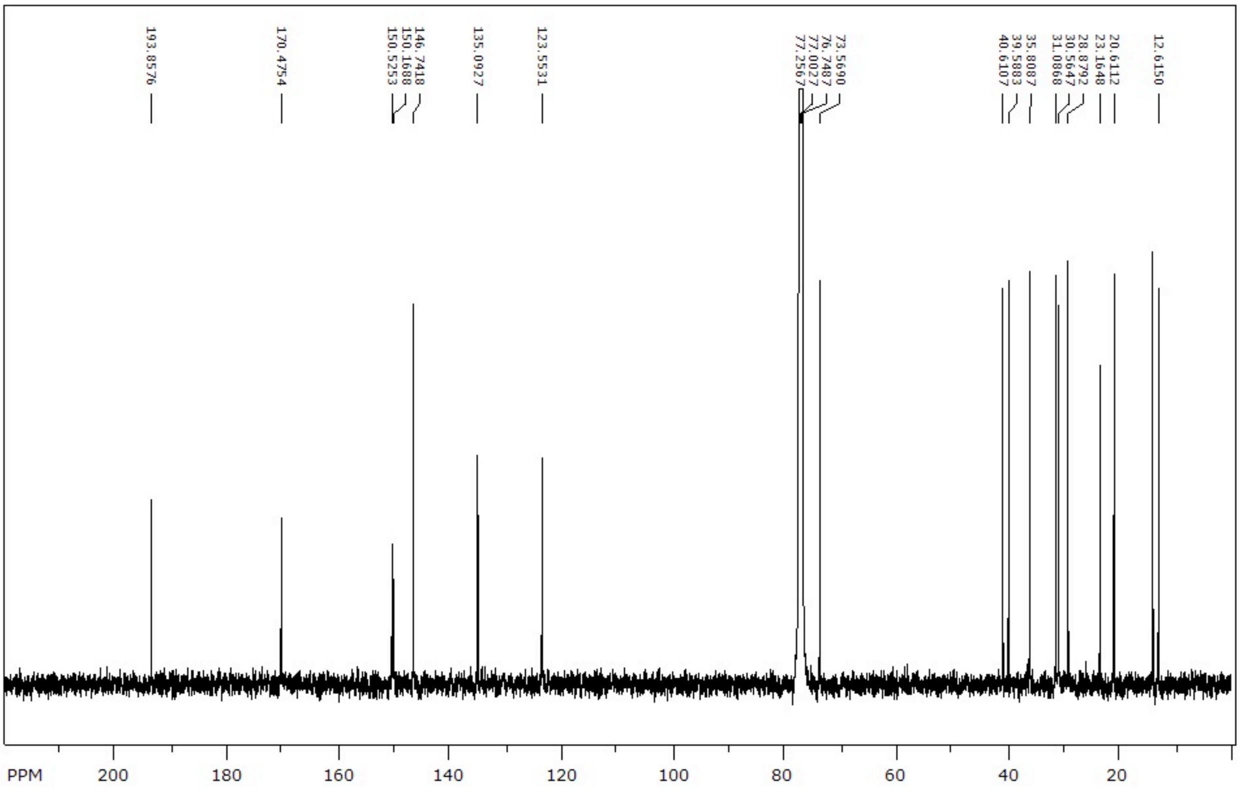

COSY NMR

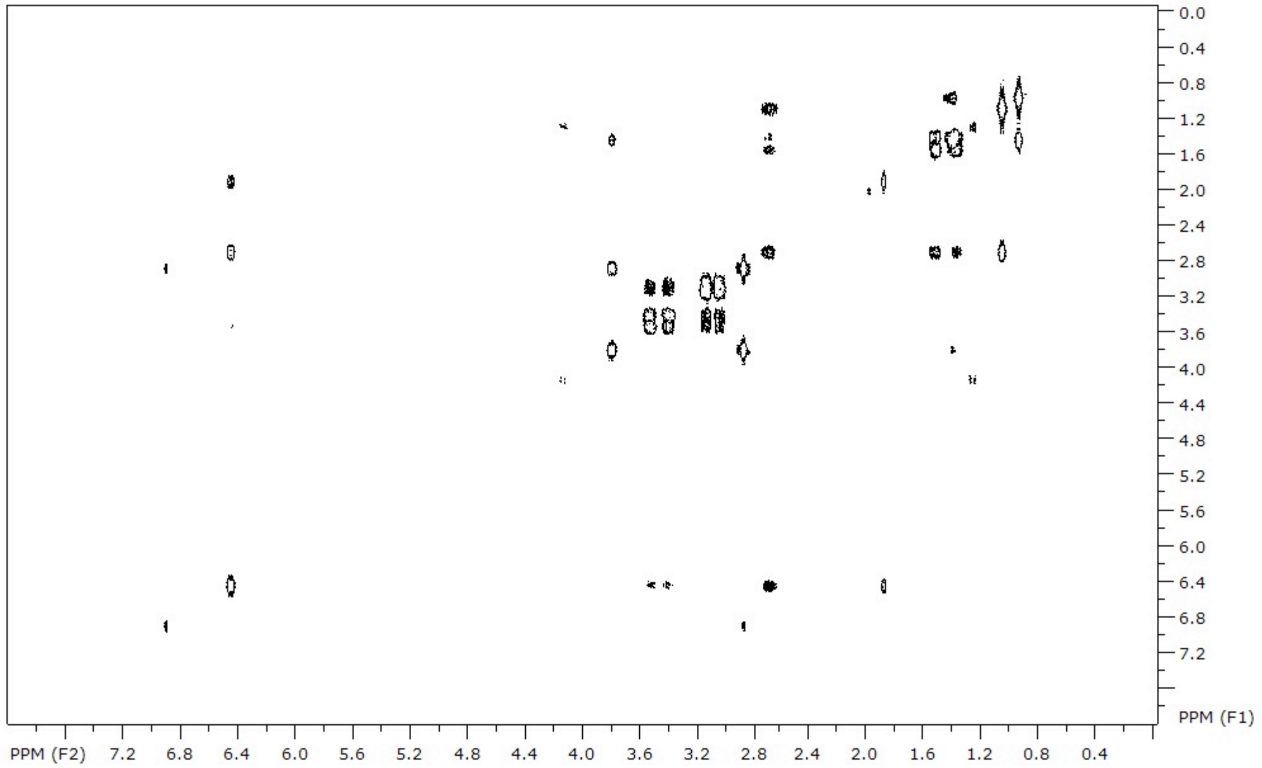

HSQC NMR

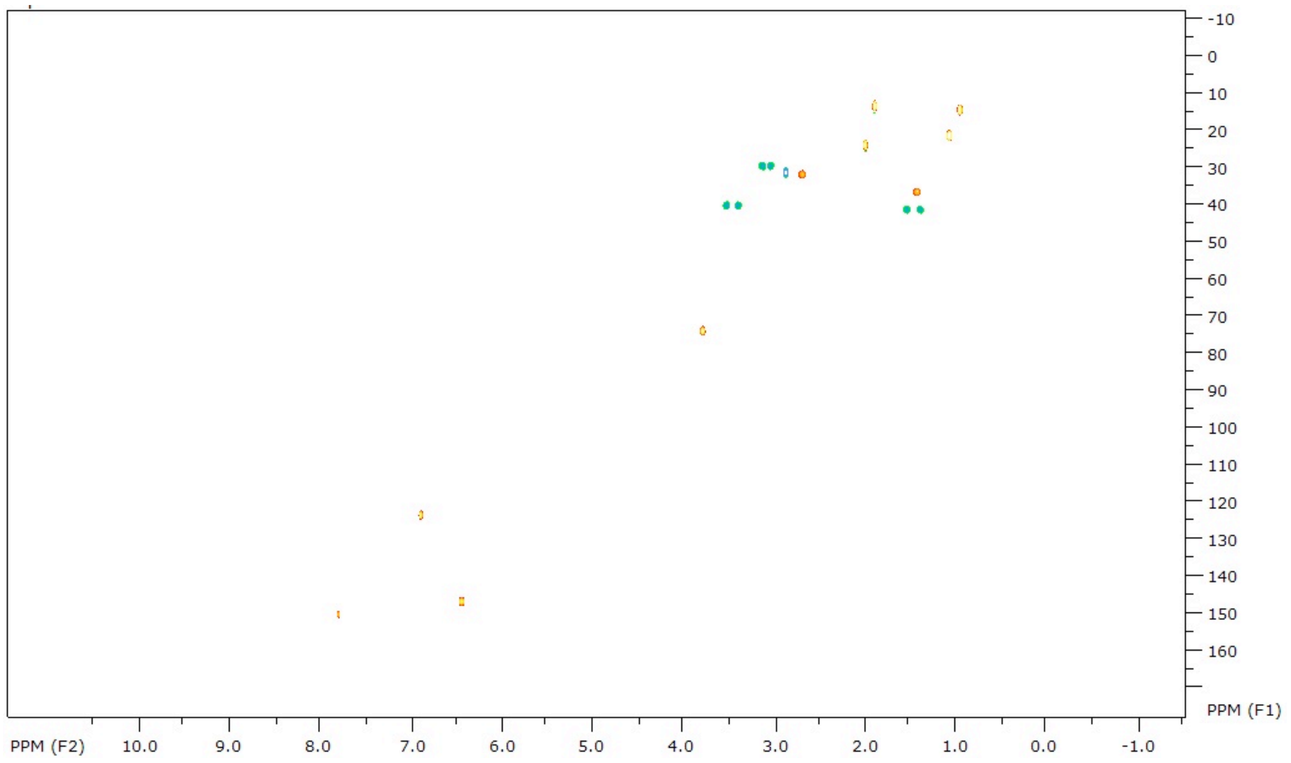

HMBC NMR

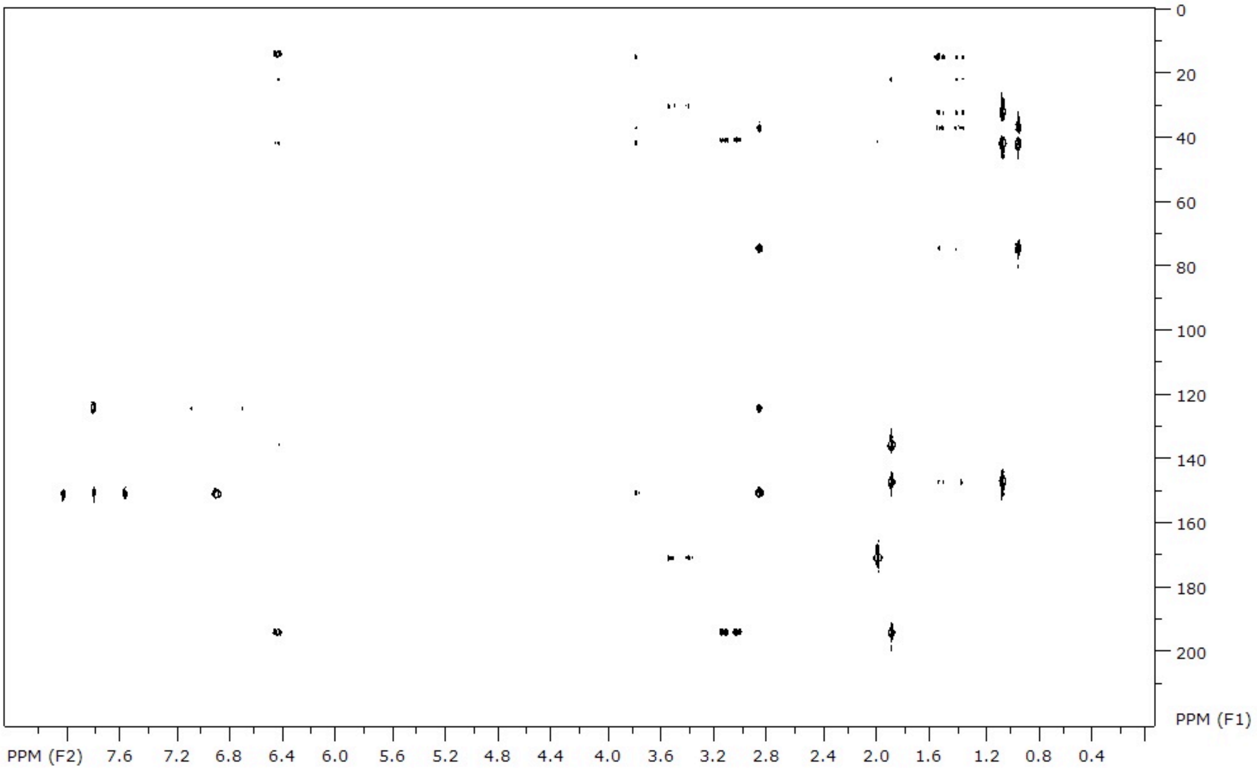

2.4. Compound 5.

^{1}H NMR

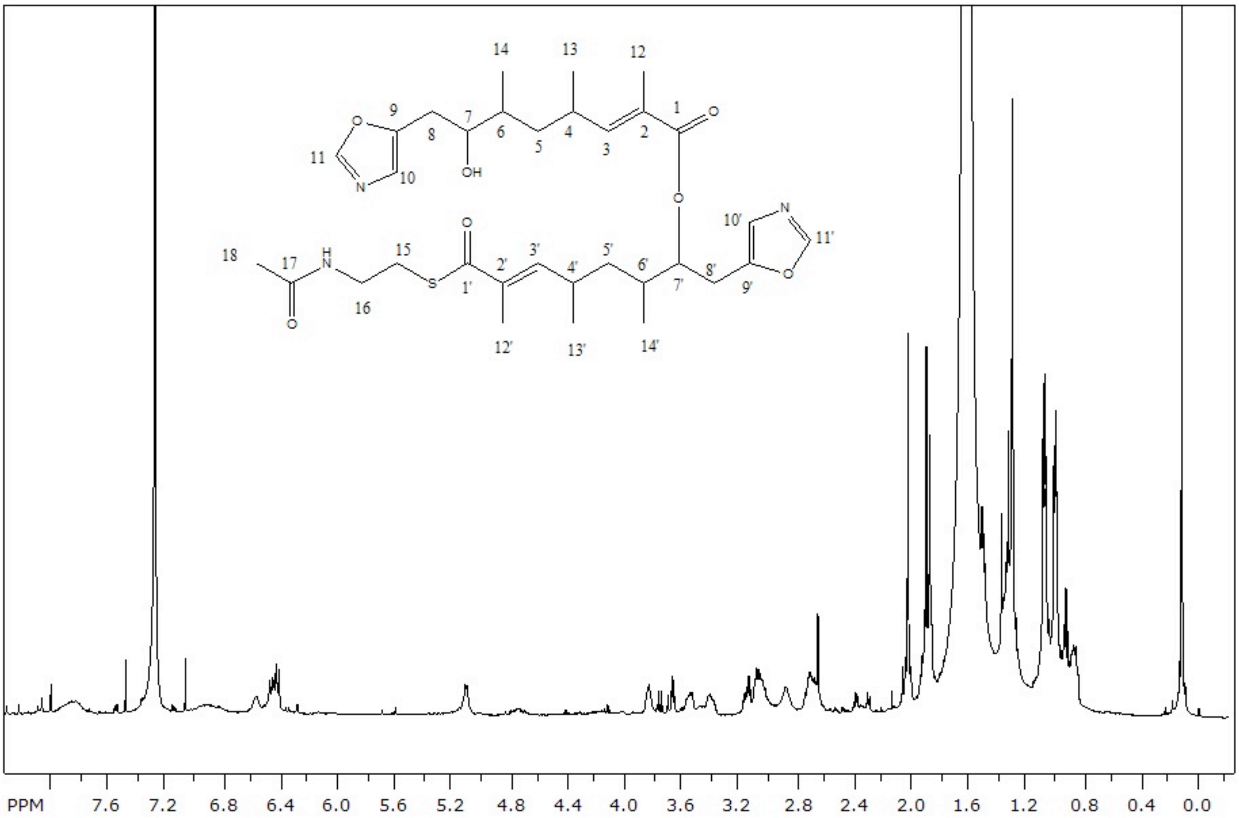

COSY NMR

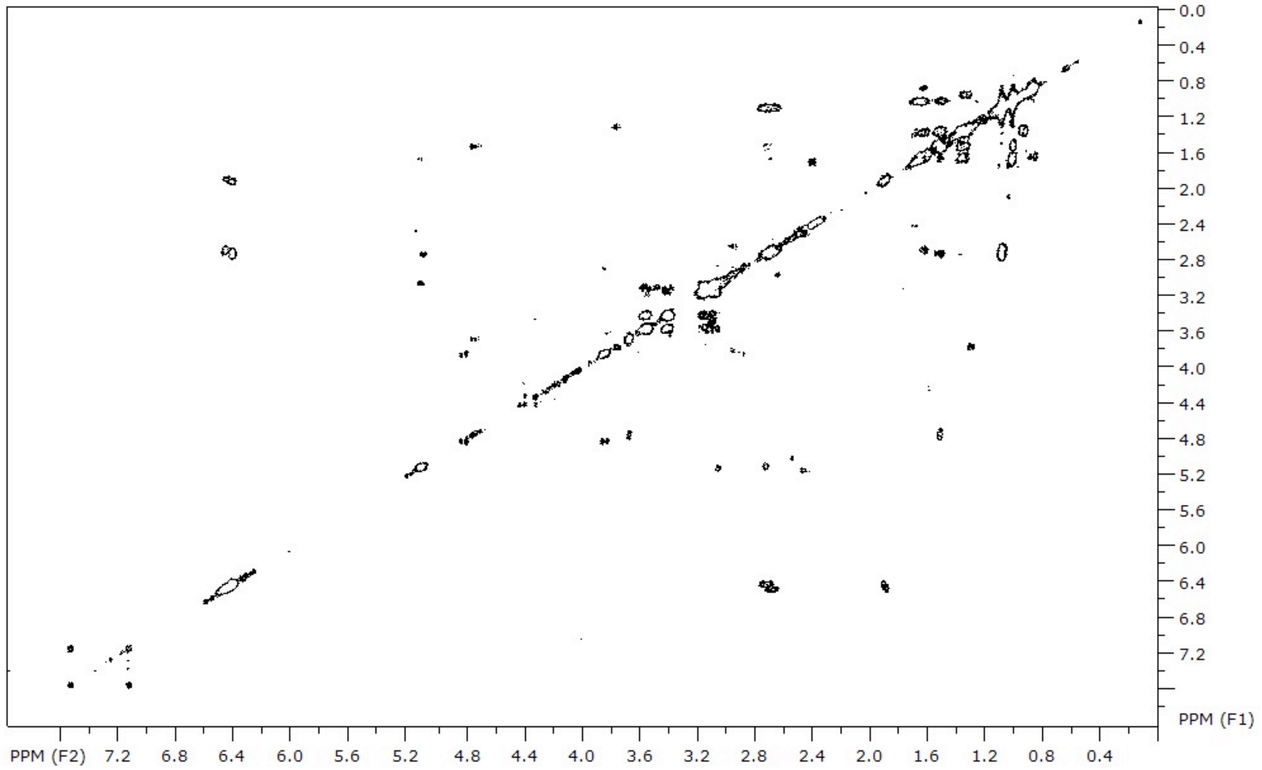

HSQC NMR

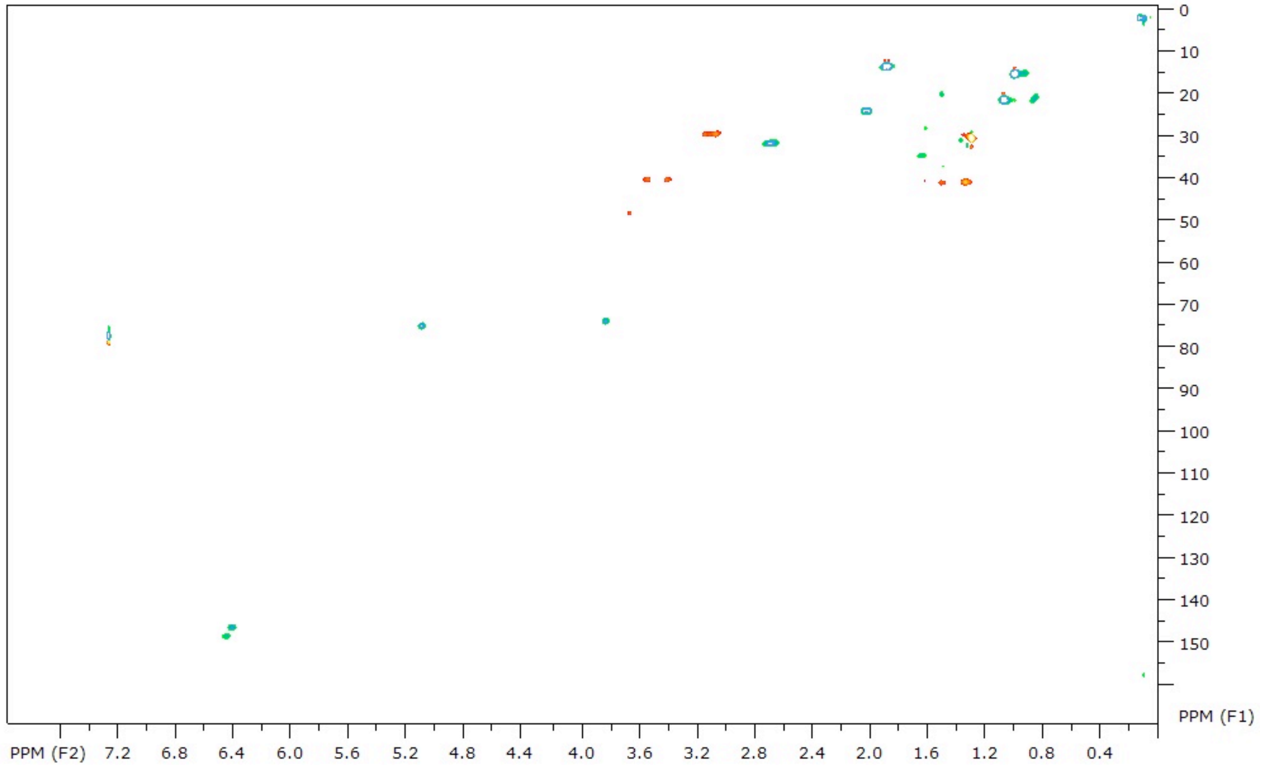

3. Supplemental References

Del Vecchio, F., Petkovic H, Kendrew SG, Low L, Wilkinson B, Lill R, Cortés J, Rudd BA, Staunton J, Leadlay PF. (2003). Active-site residue, domain and module swaps in modular polyketide synthases. J Ind Microbiol Biotechnol. *30,* 489-494.

Giraldes JW, Akey DL, Kittendorf JD, Sherman DH, Smith JL, Fecik RA. (2006). Nat Chem Biol. *2*, 531-536.

Keatinge-Clay AT. (2007). A tylosin ketoreductase reveals how chirality is determined in polyketides. Chem Biol. *14,* 898-908.

Kwan DH, Leadlay PF. (2010). Mutagenesis of a modular polyketide synthase enoylreductase domain reveals insights into catalysis and stereospecificity. ACS Chem Biol. *5*, 829-38.

Scaglione JB, Akey DL, Sullivan R, Kittendorf JD, Rath CM, Kim ES, Smith JL, Sherman DH. (2010). Angew Chem Int Ed Engl. *49*, 5726-5730.

Tsai SC, Miercke LJ, Krucinski J, Gokhale R, Chen JC, Foster PG, Cane DE, Khosla C, Stroud RM. (2001). Proc Natl Acad Sci U S A. *98*, 14808-14813.

Zhou, Y., Prediger, P., Dias, L.C., Murphy, A.C., and Leadlay, P.F. (2015). Macrodiolide formation by the thioesterase of a modular polyketide synthase. Angew. Chem. Int. Ed. Engl. doi: 10.1002/anie.201500401. [Epub ahead of print]
