# Supplementary material for: Iterative Mechanism of Macrodiolide Formation in the Anticancer Compound Conglobatin
Source: Chem Biol. 2015 Jun 18;22(6):745–54. doi: 10.1016/j.chembiol.2015.05.010 (PMC4504003; doi:10.1016/j.chembiol.2015.05.010)
Supplement: Document S2. Article plus Supplemental Information [file mmc2.pdf]

Article

Chemistry & Biology

Iterative Mechanism of Macrodiolide Formation in the Anticancer Compound Conglobatin

Highlights

- The conglobatin cluster has been cloned using a single-step in vitro procedure
- The gene cluster in a heterologous strain confers the ability to produce conglobatin
- A model for ATP-dependent heterocyclization to the oxazole ring is proposed
- The conglobatin thioesterase catalyzes cyclodimerization by an iterative mechanism

Authors

Yongjun Zhou, Annabel C. Murphy, Markiyan Samborskyy, Patricia Prediger, Luiz Carlos Dias, Peter F. Leadlay

Correspondence

pfl10@cam.ac.uk

In Brief

Zhou et al. describe the in vitro cloning from genomic DNA fragments of the intact biosynthetic gene cluster for conglobatin. This provides proof of concept for more convenient recovery of large gene clusters for both known and silent biosynthetic pathways, to enable their detailed analysis.

Zhou et al., 2015, Chemistry & Biology *22*, 745–754
June 18, 2015 ©2015 The Authors
http://dx.doi.org/10.1016/j.chembiol.2015.05.010

CellPress

CellPress

Iterative Mechanism of Macrodiolide Formation in the Anticancer Compound Conglobatin

Yongjun Zhou,[1] Annabel C. Murphy,[1] Markiyan Samborskyy,[1] Patricia Prediger,[2] Luiz Carlos Dias,[3] and Peter F. Leadlay[1,*]
[1]Department of Biochemistry, University of Cambridge, Cambridge CB2 1GA, UK
[2]Faculty of Technology, State University of Campinas UNICAMP, CEP 134840332 Limeira, São Paulo, Brazil
[3]Institute of Chemistry, State University of Campinas, UNICAMP, C.P. 6154, CEP 13084-971 Campinas, São Paulo, Brazil
*Correspondence: pfl10@cam.ac.uk
http://dx.doi.org/10.1016/j.chembiol.2015.05.010
This is an open access article under the CC BY license (http://creativecommons.org/licenses/by/4.0/).

SUMMARY

Conglobatin is an unusual C_2-symmetrical macrodiolide from the bacterium *Streptomyces conglobatus* with promising antitumor activity. Insights into the genes and enzymes that govern both the assembly-line production of the conglobatin polyketide and its dimerization are essential to allow rational alterations to be made to the conglobatin structure. We have used a rapid, direct in vitro cloning method to obtain the entire cluster on a 41-kbp fragment, encoding a modular polyketide synthase assembly line. The cloned cluster directs conglobatin biosynthesis in a heterologous host strain. Using a model substrate to mimic the conglobatin monomer, we also show that the conglobatin cyclase/thioesterase acts iteratively, ligating two monomers head-to-tail then re-binding the dimer product and cyclizing it. Incubation of two different monomers with the cyclase produces hybrid dimers and trimers, providing the first evidence that conglobatin analogs may in future become accessible through engineering of the polyketide synthase.

INTRODUCTION

Oxazole-containing polyketides are an intriguing family of natural products with diverse biological activities. Perhaps the best known are the methyloxazole-containing antitumor compound rhizoxin from "*Burkholderia rhizoxina*," a symbiont of the fungus *Rhizopus microsporus* (Partida-Martinez and Hertweck, 2007), the oxazole triene antibiotic oxazolomycin A (Figure 1, **1**) from *Streptomyces albus*, with potent cytotoxic and antibacterial activity (Mori et al., 1985), and the related herbicidal phthoxazolins/inthomycins (Omura et al., 1990; Henkel and Zeeck, 1991; Tanaka et al., 1993; Shiomi et al., 1995). Complex polyketides in bacteria are typically produced on polyketide synthases (PKSs), remarkable assembly-line multienzymes in which each cycle of chain extension is catalyzed by a different module of fatty acid synthase-related enzymatic activities. A similar logic is used in the biosynthesis of antibiotic peptides on non-ribosomal peptide synthetase multienzymes (NRPSs). The oxazole-containing polyketides are among the select group of natural products in which the biosynthetic assembly line contains both PKS and NRPS modules. A canonical PKS module minimally contains a β-ketoacyl synthase (KS) to catalyze C-C bond formation, an acyltransferase (AT) that recruits the appropriate extender unit, and an acylcarrier protein (ACP) domain. In an NRPS module, the condensation (C) domain forges the new amide bond, while the adenylation domain activates the extender amino acid and transfers it to the peptidyl carrier protein (PCP) domain. Optionally, a PKS module may also contain activities that reduce the initially formed β-ketothioester before it is handed on to the following module. These include ketoreductase (KR), dehydratase (DH), and enoylreductase (ER) domains. In many naturally occurring PKSs (including oxazolomycin), the AT activity is not an integrated domain in the multienzyme, but is present on a stand-alone protein which delivers extender units to every module (trans-AT PKSs). Many PKSs follow the assembly-line paradigm but show non-canonical placement and use of domains (non-colinearity), as well as catalyzing the incorporation of more exotic extender units, such as the gem-dimethyl group in oxazolomycin. Elucidating the molecular basis for these variations is an important step in gaining insight into the evolution of PKS systems, as well as laying the foundation for engineering the production of novel polyketides with potentially useful properties.

The 5-substituted oxazole moiety is also found in conglobatin (Figure 1, **2**), a 16-membered macrocyclic diolide originally isolated from a polyether-producing strain of *Streptomyces conglobatus* ATCC 31005 (Westley et al., 1979) and reported at that time to be essentially devoid of antibiotic or antitumor activity. Recently, however, conglobatin (under the pseudonym FW-04-806) has been reinvestigated and reported to inhibit the proliferation of cancer cell lines, to cause G2/M cell-cycle arrest, to induce apoptosis, and to downregulate client oncoproteins of heat-shock protein Hsp90 (Huang et al., 2014). Its mode of action appears to be novel, via direct binding to the N-terminal domain of Hsp90 and disruption of its interaction with co-chaperone Cdc37 (Huang et al., 2015). The related 20-membered symmetrical oxazole macrodiolides known as samroiyotmycins (Figure 1, **3**) (Dramae et al., 2013) are reported to be active antimalarial compounds. We have recently investigated the biosynthesis of the macrocyclic diolide elaiophylin (Figure 1) and have shown that the thioesterase of the elaiophylin polyketide synthase acts by an iterative mechanism, first ligating two monomers and then re-loading the linear dimer onto the

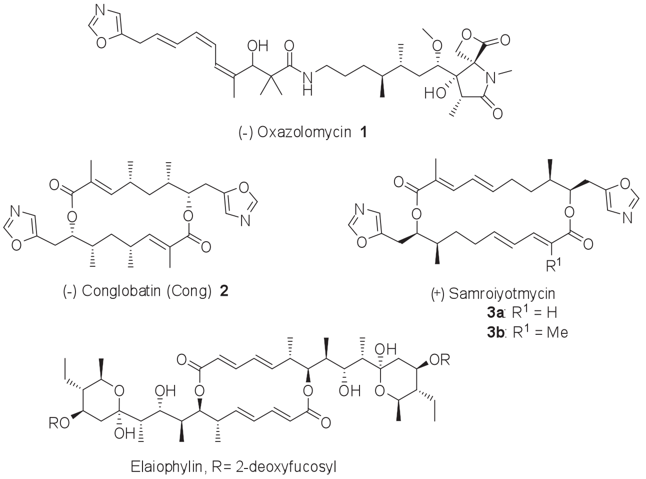

Figure 1. The Structures of Conglobatin and Related Oxazole or Macrodiolide Natural Products

thioesterase active site for dimerization (Zhou et al., 2015). This same mechanism has been previously demonstrated for the thioesterase/cyclase (TE) domain that catalyzes formation of typical non-ribosomal peptide synthetases (Shaw-Reid et al., 1999; Hoyer et al., 2007; Robbel et al., 2009). The X-ray crystal structures of chain-terminating TE domains from the PKS assembly lines have been determined for both macrocyclic polyketides (Tsai et al., 2002; Giraldes et al., 2006; Akey et al., 2006) and linear polyketides (Scaglione et al., 2010; Gehret et al., 2011), providing a valuable framework for mechanistic investigation. These enzymes belong to the α,β-hydrolase family, and the active site is located in the hydrophobic center of an unusual channel that traverses the entire protein. There are subtle differences in the size, shape, and accessibility of this channel in different structures, but it remains difficult to identify individual enzyme-substrate interactions that determine the outcome (Horsman et al., 2015). The ability of several individual polyketide TE domains to catalyze in vitro macrocyclization of thioester substrates has been demonstrated (Boddy et al., 2003; Wang et al., 2009; Pinto et al., 2012). Given the intriguing newly reported biological properties of conglobatin, we were interested in investigating its biosynthesis and defining the mechanism of dimerization in this pathway. We report here that the enzymology of formation of the oxazole ring in conglobatin closely resembles that for oxazolomycin, and that the conglobatin thioesterase follows an iterative mechanism, which has allowed its use in vitro to produce linear dimers and trimers in which the natural conglobatin seco acid monomer is combined with different polyketide building blocks.

RESULTS AND DISCUSSION

Identification of the Conglobatin Biosynthetic Gene Cluster

Conglobatin is a symmetrical polyketide macrodiolide, and inspection of its structure suggests that its assembly is governed by an NRPS/PKS biosynthetic gene cluster. From the known organization of the NRPS/PKS for oxazolomycin (Zhao et al., 2010) we expected that initiation of conglobatin biosynthesis would involve an N-terminal loading module specific for glycine, followed by four PKS extension modules, and that termination would require a C-terminal chain-terminating TE domain. A high-quality draft genome sequence was generated in-house for the conglobatin-producing strain *S. conglobatus* ATCC 31005, and screened using as probe the amino acid sequence of the loading module protein of the oxazolomycin NRPS/PKS (OzmO). This search identified a unique locus in the *S. conglobatus* genome, and detailed in silico analysis of this region strongly supported its identification as the conglobatin gene cluster (Figure 2; Table S1). Experimental confirmation of involvement in conglobatin biosynthesis was obtained after transfer of the entire region into a heterologous host, as described below. We therefore refer to it as the *cong* locus.

Sequence Analysis of the Conglobatin Biosynthetic Gene Cluster

Bioinformatic analysis of the *cong* locus revealed five contiguous open reading frames, spanning 28 kbp, whose gene products could be assigned putative roles in conglobatin biosynthesis. They encode three canonical modular PKSs (CongB, CongC, and CongD), an NRPS module (CongA), and a protein (CongE) of unknown function but with very significant sequence identity (80%) to OzmP from the oxazolomycin biosynthetic gene cluster. These genes appear to be transcribed from a single bidirectional promoter located between *congA* and *congC*. Tellingly, *congA*, *congE*, and *congB* are arranged together in exactly the same way as their counterparts *ozmO*, *ozmP*, and *ozmQ* in the oxazolomycin gene cluster. The precise limits of the cluster have not been rigorously defined, but a potential regulatory gene (*orfR1*) may provide one flank of the gene cluster, while the other may be signaled by *orf1*, encoding nicotinamide adenine dinucleotide phosphate-dependent glutamate dehydrogenase (Figure 3B; Table S1). However, among *orf*s *2–10* there are several other putative regulatory genes (Table S1) which may have roles in conglobatin biosynthesis.

One-Step Cloning of the Conglobatin Gene Cluster and Heterologous Expression of Conglobatin in *Streptomyces coelicolor*

A 41-kbp XhoI-EcoRI DNA fragment was identified that contains the five genes *congA–E*. This region includes *orfR1* on one side and *orf1* to *orf10* on the other side (Figure 3B; Table S1). OrfR1 shows 46% sequence identity to FscRI, which has been shown to act as a positive regulator in the biosynthesis of the polyene FR-008 (Zhang et al., 2015). It also includes the three additional potential regulatory genes *orf*3, *orf7*, and *orf10*. Genomic DNA, extracted and purified from 3-day-old mycelia of *S. conglobatus*, was digested with XhoI and EcoRI (Figure 3A) and fragments smaller than 20 kbp were removed by electrophoresis. To obtain a 5.3-kbp fragment of pSET152 (Bierman et al., 1992) PCR amplification was used with as template the pSET152-derived plasmid pIB139 (Wilkinson et al., 2002). The resulting linear vector fragment had 39- and 41-bp flanking regions, respectively, which are identical with the termini of the 41-kbp target DNA. Vector DNA and the chromosomal fragments were mixed and used for Gibson assembly (Gibson et al., 2009) as described in Experimental Procedures, and the products used in transformation of *Escherichia coli* (Figure 3B).

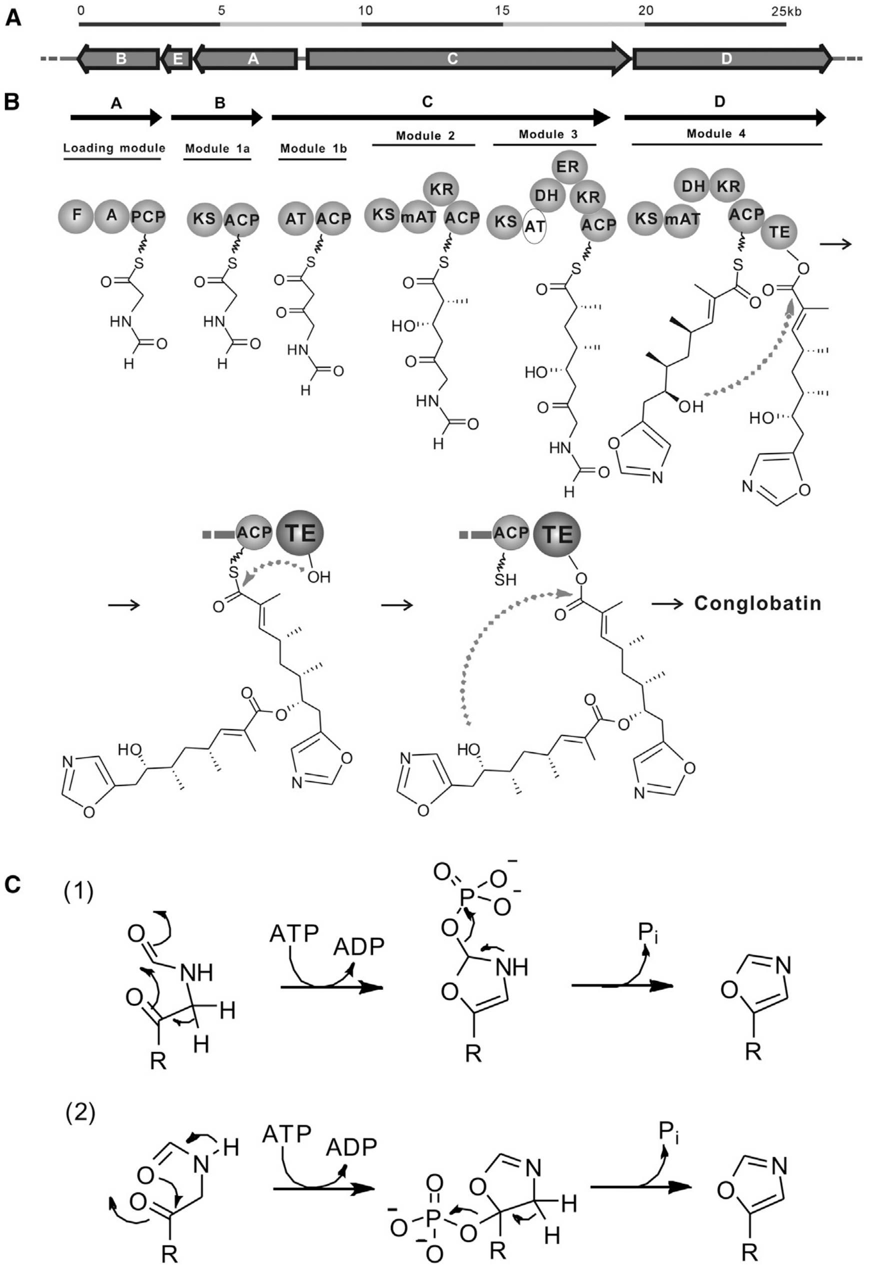

Figure 2. Gene Cluster and Proposed Pathway for Biosynthesis of Conglobatin

(A) Genetic organization of the conglobatin gene cluster (see also Table S1).

(B) Proposed biosynthetic pathway for conglobatin production in *S. conglobatus* (see the amino acid sequence alignment of each functional domain in Figure S2).

(C) Proposed process of 5-methyloxazole formation on the Cong-PKS, catalyzed by putative cyclodehydratase CongE. Phosphorylation of the amide backbone oxygen (mechanism 1) has been previously shown to promote thiazole/oxazole ring formation in microcin natural products (Dunbar et al., 2014). CongE may instead activate the amide oxygen by adenyltransfer from ATP with release of pyrophosphate. The timing of cyclodehydration with respect to the first polyketide elongation step remains to be established. In alternative mechanism 2, ATP is proposed to activate the other keto group for ring closure.

All 30 transformants tested were found to be positive by using colony PCR. The plasmid extracted from one such colony was confirmed by sequencing to have the desired insert, and was designated pYJ24.

Plasmid pYJ24 was introduced into *Streptomyces coelicolor* M1154 (Gomez-Escribano and Bibb, 2011) by conjugation. Transformants (pYJ24/M1154) were confirmed by PCR and sequencing. To test for conglobatin expression, TSBYplus medium (see Experimental Procedures) was used as both seed and production medium. After 4 days of growth, heterologous expression of conglobatin in *S. coelicolor* pYJ24/M1154 was detected using HPLC-MS (high-pressure liquid chromatography-linked mass spectrometry) analysis of extracts. The conglobatin had the same retention time and molecular ion ($[M + H]^+ = 499.4$) as authentic conglobatin produced by *S. conglobatus* (Figures 3C and S1), and produced the same MS/MS fragments from this molecular ion, with *m/z* 453.2, 204.2, and 159.3. The production level in *S. coelicolor* was less than 1% of the level produced by the original strain, which suggests that regulation of conglobatin biosynthesis is far from optimal under these conditions. It may also indicate that additional *S. conglobatus* genes are required for efficient biosynthesis. Nevertheless, this finding provides important confirmation of the identity of the cloned *cong* locus. It also paves the way for future analysis and manipulation of the cluster in a convenient heterologous strain. The efficiency with which the *cong*-bearing genome fragment was cloned from genomic DNA is highly encouraging. It remains to be established whether this direct selection method can be adapted for more general use (and for higher throughput) in whole-cluster transfer into heterologous strains.

Proposed Model of Conglobatin Biosynthesis

Assembly of the conglobatin monomer on the *cong* NRPS-PKS appears to be for the most part "co-linear," as indicated in Figure 2B. Chain initiation takes place on the multienzyme CongA, whose adenylation domain is predicted using NRPSpredictor2 (Röttig et al., 2011) to specifically activate the amino acid glycine. The formyltransferase (F) domain shows 23%, 38%, and 59% sequence identity, respectively, to F domains in the initiation modules of the linear gramicidin NRPS (Schönafinger et al., 2006), the PKS-NRPS for the macrodiolide rhizopodin (Pistorius and Müller, 2012), and the oxazolomycin PKS (Zhao et al., 2010).

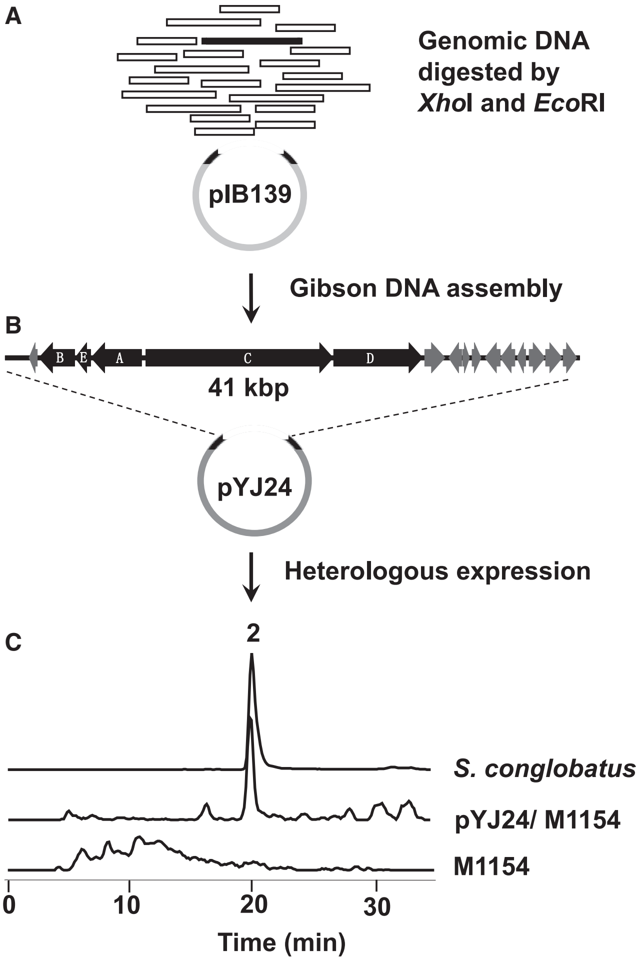

Figure 3. One-Step Cloning and Heterologous Expression of the Conglobatin Gene Cluster

(A) A 41-kbp XhoI-EcoRI DNA fragment (black) containing the five genes *congA–E* is generated by XhoI and EcoRI digestion of total genomic DNA. A 5.3-kbp pSET152 fragment was obtained by PCR amplification using as template the pSET152-derived plasmid pIB139. The resulting linear vector fragment had 39- and 41-bp flanking regions, respectively, identical to the termini of the target DNA (see Experimental Procedures).

(B) Gibson assembly leads to specific cloning of the target fragment, to give the bifunctional *E. coli-Streptomyces* plasmid pYJ24. The deduced open reading frame functions in the fragment are given in Table S1.

(C) Heterologous expression in *S. coelicolor* M1154 is confirmed by HPLC-MS and comparison with authentic compound produced by *S. conglobatus* (see also Figure S1). The mass extraction of *m/z* 499–500 is used to display the data. The y axis scale of *S. conglobatus* is 20 times larger than that of pYJ24/M1154 or M1154.

Marahiel's group (Schönafinger et al., 2006) has demonstrated for linear gramicidin in vitro that the F domain acts, using either N^{10}- or N^5-formyltetrahydrofolate as the formyl group donor, on the initiator amino acid tethered by a thioester linkage to the PCP domain, and also that subsequent chain extension does not proceed without prior formylation. The conglobatin F domain is analogously proposed to act on the glycyl residue tethered to the PCP. The formylglycine thioester could conceivably undergo cyclization at this stage, or could first be transferred via KS1 to the apparently redundant ACP domain C terminus of KS1. Alternatively, cyclization may occur after condensation of formylglycine with malonyl-CoA within extension module 1, which is split between multienzymes CongB and CongC.

The mechanism of cyclodehydration to form an oxazole is not known but clearly resembles the equivalent process in oxazolomycin biosynthesis. In many previously studied examples of oxazole and thiazole formation on assembly-line systems, where an amino acid side chain is involved as a nucleophile, a modified C domain, referred to as a heterocyclization domain, catalyzes formation of the oxazoline or thiazoline, and an oxygenase domain catalyzes subsequent flavin-dependent oxidation to the aromatic ring (Roy et al., 1999; Schneider et al., 2003). Neither of these domains is present in the *ozm* or *cong* gene clusters. Instead, the enigmatic protein CongE (highly similar to OzmP in the oxazolomycin biosynthetic gene cluster) may be responsible for cyclodehydration. Both these proteins belong to the family of N-type ATP (pyro)phosphohydrolases and contain the signature motif SGGKDS for ATP binding (Bork and Koonin, 1994).

Modeling of CongE using Phyre2 (Kelley and Sternberg, 2009) indicates that it possesses the α-β-α domain fold of this phosphohydrolase family and identifies significant structural homology with enzymes using ATP to activate amides for nucleophilic attack, for example, tRNA thiouridine synthetase TtuA from *Pyrococcus horikoshii* (Nakagawa et al., 2013). This is intriguing because ATP-dependent cyclodehydratases of the (unrelated) YcaO family have recently been convincingly shown to be responsible for ring formation in the biosynthesis of thiazole/oxazole-modified microcins (Dunbar et al., 2014). It is therefore tempting to propose that CongE and OzmP might phosphorylate (or alternatively transfer AMP to) an amide carbonyl oxygen to promote oxazole formation (Figure 2C). A further mechanistic precedent for CongE to catalyze ATP-dependent cyclodehydration is the reaction catalyzed by PurM, which catalyzes the ATP-dependent heterocyclization of formylglycinamide ribonucleotide to aminoimidazole ribonucleotide as part of purine biosynthesis (Schrimsher et al., 1986; Dunbar et al., 2012). Biochemical studies to test these models for oxazole formation in conglobatin are under way.

One obvious difference between the *ozm* and *cong* NRPS-PKS multienzymes is that the Cong multienzyme is a canonical *cis*-AT system, rather than having the AT as a stand-alone protein operating in *trans*. All *cong* KS domains showed the presence of the essential active site cysteine and two histidine residues. Domain KS1 differs significantly from KS2, KS3, and KS4 (Figure S2D). The AT of extension module 1 is predicted to recruit malonyl-CoA to provide the extender unit, while AT2 and AT4 are predicted to select (2*S*)-methylmalonyl-CoA (Haydock et al., 1995; Yadav et al., 2003), in agreement with the observed structure of conglobatin. However the active site motif GXSXG (where serine is the catalytically essential residue) is replaced in AT3 by GQAVG, so this domain is evidently inactive (Figure S2A). Our working hypothesis is that the KS2 carries out two successive condensations ("stuttering") before passing the polyketide chain to module 3 for reduction. Such a programmed iteration has previously been invoked for PKS modules of stigmatellin (Gaitatzis et al., 2002), aureothin (He and Hertweck, 2003), borrelidin (Olano et al., 2003), and crocacin

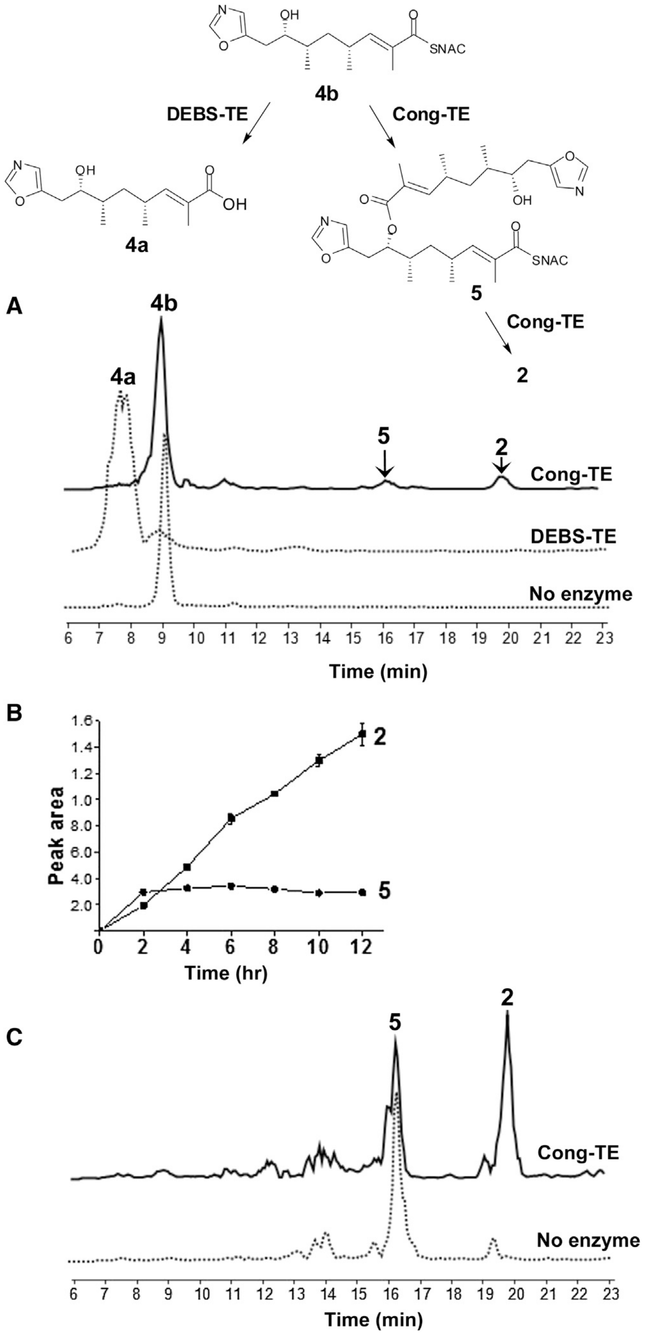

Figure 4. In Vitro Reconstitution of Conglobatin Macrodiolide Formation Catalyzed by Conglobatin Thioesterase

(A) Conglobatin thioesterase (Cong-TE) catalyzed homodimerization of thioester **4b** into **5** and conglobatin **2**, analyzed by HPLC-MS. In contrast, the erythromycin PKS thioesterase (DEBS-TE) hydrolyzed **4b** to **4a**. The structures of **2**, **5**, **4a**, and **4b** were determined by high-resolution (HR)-MS (Table S2), and 1D and 2D NMR analysis (see Experimental Procedures and Supplementary Information).

(B) Time course of production of **5** and **2** catalyzed by Cong-TE.

(C) Linear thioester dimer **5**, re-incubated with fresh Cong-TE, is converted into conglobatin **2**.

This scheme is used to summarize all the conversions. Protein gel is given in Figure S3.

(Müller et al., 2014) biosynthesis. If such a mechanism operates here, the second round of chain extension within module 2 must be followed by immediate transfer of the ketoacylthioester to module 3 because the KR of module 2 is predicted to produce the "wrong" configuration for subsequent dehydration and enoylreduction (see below). The cluster contains a DH domain in each of modules 3 and 4, both predicted to be active as required for the conglobatin structure. Likewise, the three KR domains in the PKS all contain the conserved active site residues expected of active enzymes. The stereochemistry of reduction by the KR in module 2 is predicted, from the presence of characteristic active site motifs (Caffrey, 2003; Reid et al., 2003; Keatinge-Clay, 2007), to give rise to a 2*R*,3*S*-acyl thioester intermediate, consistent with the observed conglobatin structure (Figure S2B), and similarly a characteristic motif (Kwan and Leadlay, 2010) in the ER domain of extension module 3 correctly predicts the formation of a 2*R*-acyl thioester intermediate as required for conglobatin (Figure S2C) (Schregenberger and Seebach, 1984).

Reconstitution of Conglobatin Macrodiolide Formation In Vitro

To study the selectivity and mechanism of the C-terminal TE domain of the Cong-PKS, we expressed this domain as a stand-alone protein in *E. coli* and purified it to homogeneity (Figure S3). The boundaries of the domain were selected on the basis of alignment with homologous TE domains of known structure (Figure S2E). The substrate for dimerization was obtained by alkaline hydrolysis of conglobatin to the monomeric seco acid **4a**, and subsequent conversion of this into its *N*-acetylcysteaminyl (SNAC) thioester **4b** (Figure 4A; Experimental Procedures). After 5 hr of incubation of the Cong-TE with **4b** at 20°C, the reaction was stopped and the mixture was analyzed using HPLC-MS. Two new peaks were detected in the total ion current, one (at 20.0 min) identical to conglobatin **2** and a second (at 16.3 min) with a mass corresponding to that of the linear dimer as its SNAC thioester **5**. In control assays without enzyme, neither **5** nor **2** was detected. When the macrocycle-forming thioesterase from the erythromycin PKS (DEBS-TE) was used instead of Cong-TE, hydrolysis to **4a** was observed, not dimerization (Figure 4A).

The timing the formation of **5** and **2** was investigated by measuring the area of the extracted ion peak at 2-hr intervals over 12 hr of incubation. The production level of **2** increased continuously while that of **5** reached a plateau by 2 hr (Figure 4B), suggesting that **5** might be an intermediate in diolide formation. To verify this, **5** was isolated from the assay mixture and re-incubated with fresh Cong-TE for 5 hr. Analysis showed significant conversion of **5** into conglobatin **2** (Figure 4C). The Cong-TE assay was scaled up 300-fold and the structures of purified **5** and **2** were elucidated by using 1D and 2D nuclear magnetic resonance (NMR) and high-resolution MS (Table S2; NMR data are shown in Supplemental Information).

The chemical competence of **5** as an intermediate in conglobatin formation in vitro strongly supports an iterative mechanism for TE catalysis, as we recently proposed for biosynthesis of the symmetrical 16-membered diolide elaiophylin (Zhou et al., 2015). In this mechanism, the full-length conglobatin monomer attached to the TE active site is attacked by the distal hydroxy

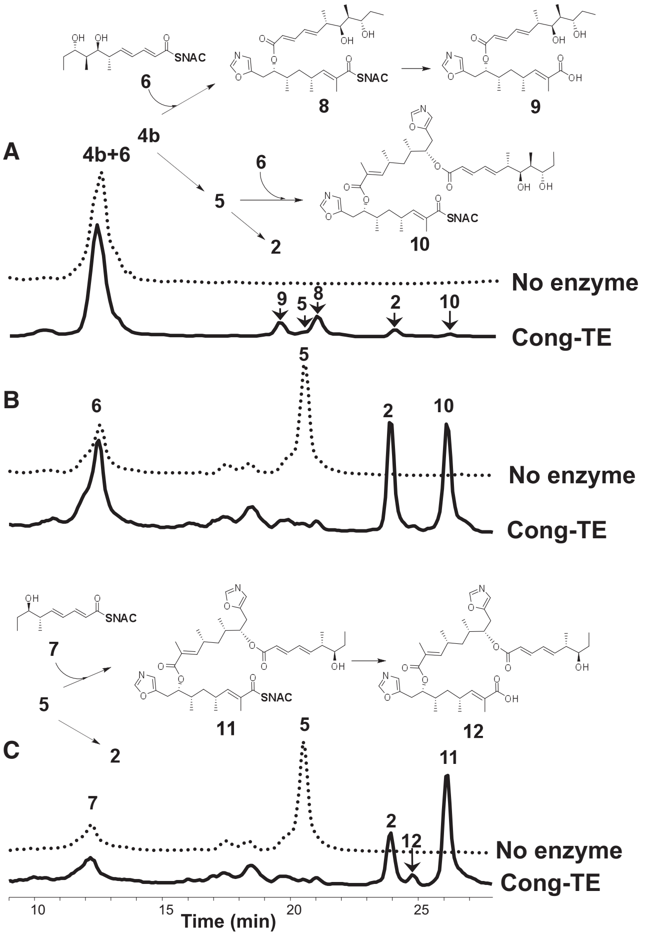

Figure 5. Hybrid Polyketide Dimers and Trimers Generated by Cong-TE from Mixed Substrates

(A) Conglobatin thioesterase (Cong-TE) in the presence of thioesters **4b** and **6** catalyzed homodimerization of **4b** into **5** and conglobatin **2**, and heterodimerization of **4b** and **6** into both linear heterodimer **8** (and its hydrolysis product **9**) and linear heterotrimer **10**.

(B) Linear thioester dimer **5**, re-incubated with fresh Cong-TE in the presence of **6**, is converted into conglobatin **2** and into heterotrimer **10**.

(C) Linear thioester dimer **5**, re-incubated with fresh Cong-TE in the presence of **7**, is converted into **2** and into heterotrimer **11** (and its hydrolysis product **12**). Details of the MS/MS and HR-MS analysis are given in Figure S4 and Table S2, respectively.

group of an identical monomer on the adjacent ACP. The linear dimer produced then re-acylates the TE active site, and finally macrocyclization is catalyzed (Figure 2B). For the elaiophylin TE (ElaTE) in vitro, it was shown that a pentaketide substrate analog **6** gave rise to a symmetrical 16-membered decaketide diolide analog of elaiolide (Zhou et al., 2015). More surprisingly the tetraketide analog **7**, although not itself a substrate, when mixed with **6** and presented to the ElaTE, gave rise to an additional compound, an asymmetrical nonaketide diolide. These results are readily accommodated by the iterative mechanism, since the various acyl transfers to and from the TE may well have different substrate preferences. We were therefore interested in the present work to discover whether **6** or **7** (neither of which is itself a substrate for the Cong-TE) could similarly intervene in the formation of novel dimers, when mixed with the natural seco acid analog **4b**.

Formation of Hybrid Polyketides Using Cong-TE In Vitro

Assays with mixtures of substrates were carried out using the same conditions as for reaction with **4b** alone, except that incubation was for 12 hr. We expected that the analog of the natural substrate for Cong-TE would preferentially acylate the TE but that we might see "cross-coupled" dimers through the nucleophilic attack of **6** on the acyl-enzyme in competition with a second molecule of **4b**. To our initial surprise, co-incubation of **4b** with **6** produced, in addition to the expected **5** and **2**, a new compound whose structure was determined as the SNAC thioester of a hybrid linear dimer **8** (Figures 5A and S4A). This was accompanied by the corresponding carboxylic acid **9**. These cross-coupled products have clearly been formed by nucleophilic attack of **4b** on the **6**-derived acyl-TE intermediate. The hybrid molecule **8** is able to re-acylate the TE and undergoes hydrolysis to **9** in preference to macrocyclization. Surprisingly, a further product was identified as the linear trimeric molecule **10** (Figures 5A and S4C). To confirm the likely mechanism for production of **10**, **5** was purified and co-incubated with Cong-TE in the presence of **6**. As shown in Figure 5B, **5** was converted into a mixture of conglobatin **2** and trimer **10**. The formation of **10** is evidently caused by nucleophilic attack of **5** on the **6**-derived acyl-TE intermediate in competition with macrocyclization.

We extended these findings by co-incubation of the linear homodimeric SNAC thioester **5** with the tetraketide **7**. Analysis of this mixture after 12 hr showed formation of trimer thioester **11** as well as the product of thioester hydrolysis of this compound, **12** (Figures 5C and S4D). We conclude from these findings that the elaiophylin-related thioesters **6** and **7** are both reactive enough (and the Cong-TE flexible enough) to compete successfully with **4b** for acylation of the Cong-TE. However, deacylation is selective, hinting at the presence of a specific binding site on the protein for the incoming nucleophile. Unable to exploit this, nucleophilic attack by water (hydrolysis) or a second molecule of **6** or **7** is slow. In contrast, the conglobatin precursor analogs **4b** and **5** are effective in deacylation, leading to the products described in Figure 5. These results reveal the subtlety of the iterative mechanism operated by macrodiolide TE domains. Meanwhile, the availability of the conglobatin gene sequence will enable engineering of the pathway to produce non-natural conglobatins as potential antitumor compounds. Given their close structural similarity (Figure 1), the conglobatin pathway might also be engineered to provide analogs of the 20-membered antimalarial oxazole macrodiolides **3**, known as samroiyotmycins.

SIGNIFICANCE

The C-terminal chain-terminating thioesterase domains of modular polyketide synthase assembly lines have attracted great interest because they exert a decisive influence on whether polyketide products are released in linear or cyclic form, and on the mode of any cyclization. An understanding of their specificity is also central to the success of attempts to manipulate assembly-line polyketide synthases to synthesize altered products. The thioesterase domains that lead to formation of conglobatin, elaiophylin, and other

C2-symmetrical macrocyclic dilactones are especially intriguing because they apparently bring about the head-to-tail dimerization of identical chains using a single thioesterase active site. We have shown that a recombinant thioesterase can catalyze dimerization to conglobatin in vitro, and that it does so by an iterative mechanism, as shown recently for elaiophylin. Further evidence for the iterative operation of the thioesterase has been provided by finding that the recombinant enzyme can combine two different monomers into hybrid linear dimers or even trimers, suggesting its use as a means of producing diverse complex polyketides from relatively simple building blocks. Meanwhile, the discovery of the *cong* gene cluster, its direct one-step cloning, and the successful demonstration of conglobatin production in a convenient heterologous host together pave the way to deconvolution of the detailed enzymology of oxazole ring formation on the polyketide synthase, and to engineered production of novel conglobatins as potential antitumor compounds. Finally, this work provides proof of concept for using DNA fragments bearing an intact target gene cluster for rapid in vitro capture by Gibson assembly and direct cloning in *E. coli*. Converted to high throughput, this could be a valuable part of a pipeline for identifying the products of silent or cryptic gene clusters by controlled expression in heterologous host strains.

EXPERIMENTAL PROCEDURES

Bacterial Strains and Culture Conditions

S. conglobatus ATCC 31005 (Westley et al., 1979) was grown in TSBY medium (3% tryptone soy broth, 10.3% sucrose, 0.5% yeast extract) at 30°C and 200 rpm to produce mycelium for genomic DNA extraction. For conglobatin production, the seed medium was 3% soybean flour (the supernatant after first autoclaving was used), 5% glucose (autoclaved separately), 0.5% $CaCO_3$, 5 mg/l $CoCl_2 \cdot 6H_2O$, and 0.2% (v/v) anti-foam. For the production medium, $CoCl_2 \cdot 6H_2O$ was omitted. Fermentation was carried out by inoculating 50 ml medium in a 250-ml conical flask fitted with a metal spring, with 10% (v/v) of a 3-day seed culture, then incubating at 30°C, 200 rpm for 5 days.

E. coli DH10B was used for DNA manipulation. *E. coli* ET12567 (pUZ8002) was used for intergeneric conjugation. *E. coli* BL21 CodonPlus (DE3) and BL21 (DE3) plysS were used for protein expression.

DNA Manipulation

Restriction endonucleases and T4 DNA ligase were purchased from New England Biolabs. Chemicals were purchased from Sigma-Aldrich. Plasmid DNA was isolated from an overnight culture using the Plasmid Mini Kit I (Omega BioTek) according to the manufacturer's protocol. PCR amplification was carried out using Phusion High-Fidelity PCR Master Mix from New England Biolabs (for cloning), or BioMix Red from Bioline (for screening purposes). Genomic DNA isolated from 3-day mycelium was used as a template for PCR. DNA sequencing was carried out by the DNA Sequencing Facility in the Department of Biochemistry, University of Cambridge.

Plasmid Construction

The DNA fragment encoding Cong-TE was amplified from genomic DNA of *S. conglobatus* ATCC 31005 by PCR using oligonucleotides Cong-TE-S (5′-ATTATCATATGAGCACGGGCCTGTGCCGGCACCT-3′ [NdeI]) and Cong-TE-A (5′-ATTATCTCGAGCCGGCGGTCCGCCGGAGCGT-3′ [XhoI]). The PCR product was digested with NdeI and XhoI before introduction into the corresponding sites of pET29b(+). The resulting plasmid was designated pYJ41. General procedures for *E. coli* manipulation were carried out according to Sambrook and Russell (2001).

Single-Step Cloning of the Conglobatin Gene Cluster

S. conglobatus genomic DNA was digested with XhoI and EcoRI (FastDigest, Thermo Scientific) at 37°C for 3.5 hr. The digested DNA was fractionated by electrophoresis using 0.6% agarose gel, 40 V, for 15 hr. The gel fraction containing fragments larger than 20 kbp was recovered, the agarose was melted at 50°C, and 200 μl of phenol/chloroform mixture was added and mixed. After centrifugation, the supernatant was moved to another tube and mixed with 0.6 volumes of isopropanol. The mixture was incubated at −20°C for 10 min before precipitating the DNA by centrifugation for 10 min. After washing with 80% ethanol, the DNA was dissolved in 20 μl of water, giving a concentration of 67 ng/μl.

The vector DNA, a 5.3-kbp pSET152 fragment (Bierman et al., 1992), was obtained by PCR amplification using as template the pSET152-derived plasmid pIB139 (Wilkinson et al., 2002) linearized by NdeI and EcoRV, and oligonucleotides pSET152-cong-S (5′-GGCGGAGGCGGCGAGGTCGCGTCACCCGACGGCGGTGCCCAATTCCACACAACATACGAG-3′) and pSET152-cong-A (5′-TGCCGGACACTGGTGGATCATGCAGGACCCGGAAGGCAACGAACTTCTCGACAGACGTAGATC-3′), which contain 39 and 41 bp (underlined) and overlap respectively with the ends of the 41-kbp target DNA. The 5,271-bp PCR product was purified and concentrated to 202 ng/μl. For DNA assembly, 0.5 μl of vector DNA and 4.5 μl of genomic DNA were added to 15 μl of Gibson DNA assembly solution (Gibson et al., 2009). The reaction was carried out at 50°C for 1 hr, then 10 μl of the reaction was used for calcium-assisted transformation of DH10B. Around 100 transformants were obtained, 30 of which were confirmed as positive by using colony PCR with the primers ConConfir-S (5′-AGGACCTCACCACCTGGGAAAC-3′) and ConConfir-A (5′-TAGGTCCGCAGGGTCTGAGGCA-3′). The identity of the 509-bp PCR product was confirmed by sequencing.

Heterologous Expression of the Conglobatin Pathway

The resulting plasmid pYJ24 was introduced into *S. coelicolor* M1154 (Gomez-Escribano and Bibb, 2011) by conjugation. For selection of *Streptomyces* transformants, apramycin and nalidixic acid were used at concentrations of 25 μg/ml. Transformants (pYJ24/M1154) were grown up in TSBY medium supplemented with 25 μg/ml apramycin for genomic DNA extraction, PCR, and sequencing confirmation. To check conglobatin expression, TSBY plus medium (1 l of TSBY medium supplemented with 5 mM $MgCl_2$ and 2 ml of trace element solution as used for R2YE medium [Kieser et al., 2000]) was used as both seed and production medium. For production, a 3-day-old seed culture (supplemented with 25 μg/ml apramycin) was inoculated into 10 volumes of fresh medium. After 4 days, compound **2** was extracted from 1 ml of broth by incubation with 0.5 ml of ethyl acetate for 30 min at 50°C. The organic phase was separated and the solvent was removed, the residue was dissolved in 100 μl of methanol, and 50 μl of this was subjected to LC-MS analysis.

Protein Expression and Purification

Plasmid pYJ41 was introduced into *E. coli* BL21 CodonPlus (DE3) for Cong-TE protein expression, and pKJW63 (Tran et al., 2008) was introduced into *E. coli* BL21 (DE3) plysS for DEBS-TE protein expression. A single colony was inoculated into 5 ml of LB medium containing 50 μg/ml kanamycin and grown overnight at 37°C. 2 ml of the overnight culture was inoculated into 1 l of LB medium containing 50 μg/ml kanamycin and incubated at 37°C, 200 rpm until A_{600} reached 0.6–0.8 before adding 200 μl of 1 M isopropyl-β-D-thiogalactopyranoside and incubating at 22°C for 15 hr to induce protein expression. Cells were pelleted at 11,325 × *g* for 5 min, resuspended in lysis buffer (50 mM Tris-HCl, 0.3 M NaCl [pH 7.2]), and lysed by sonication. The total lysate was centrifuged at 34,925 × *g* for 25 min, and the supernatant was passed through a 0.45-μm filter before loading onto a His-Bind affinity column (1 ml bed volume). The column was washed with 10 column volumes of lysis buffer. Bound proteins were eluted by stepwise increases in the concentration of imidazole (up to 500 mM). Cong-TE and DEBS-TE were eluted from their respective columns at imidazole concentrations of 100 and 80 mM. The proteins were concentrated and buffer was exchanged into 100 mM potassium phosphate buffer (pH 8.2) using Amicon Ultra-4 concentrators (Millipore) fitted with a filter of 10-kDa cut-off. The yield of Cong-TE and DEBS-TE was 6 mg/l and 0.5 mg/l, respectively. The purified proteins were analyzed by 4%–12% Bis-Tris Gel (Novex) SDS-PAGE. Protein concentrations were measured using a NanoDrop 1000 spectrophotometer.

Enzyme Assays

In vitro assays contained, in a total volume of 50 μl, 40 μM Cong-TE or DEBS-TE, 3 mM substrate, and 10% (v/v) DMSO 100 mM potassium phosphate (pH 8.2). After either 5 or 12 hr of incubation at 20°C, the reaction was stopped by adding 200 μl of acetonitrile. After centrifugation, 40 μl of supernatant was injected for HPLC-MS analysis. To accumulate a sufficient amount of **2** and **5** for NMR analysis, the assay was scaled up to 15-ml system with 50 μM Cong-TE, 6 mM substrate **4b**, and 4 hr of incubation at 20°C.

HPLC-MS Procedures

HPLC-MS analysis was performed using an HPLC (Agilent Technologies 1200) coupled to a Thermo Fisher LTQ mass spectrometer fitted with an electrospray ionization (ESI) source. The HPLC was fitted with a Prodigy 5μ C18 column (4.6 × 250 mm, Phenomenex). A solvent system of acetonitrile and water both containing 0.1% formic acid (v/v) was used. Samples were eluted at a flow rate of 0.7 ml min^{-1} with a linear gradient of 40%–100% acetonitrile over 30 min. The mass spectrometer was run in positive ionization mode, scanning from *m/z* 200 to 2000 using a normalized collision energy of 35%. Preparative HPLC purification was performed on an Agilent Technologies 1200 apparatus, using a C18 column (100 Å, 250 × 21.20 mm, 10 μm; Phenomenex) at a flow rate of 15 ml min^{-1}. Sample injection volume was 200 or 500 μl. ESI high-resolution MS was carried out on a Thermo Fisher Orbitrap with 30,000 resolution.

NMR Analysis

NMR spectra were recorded on a Bruker 500-MHz DCH Cryoprobe Spectrometer except for compound **5**, which was recorded on a Bruker 500-MHz TCI Cryoprobe Spectrometer, and compound **4a**, which was recorded on a Bruker 400-MHz Avance III HD Spectrometer. Chemical shifts are expressed in parts per million on the δ scale, referenced to C**H**Cl_3 at δH 7.26 (^{1}H) and **C**HCl_3 at δ C 77.0 (^{13}C). Where ^{1}H signals were obscured by other signals or contaminants, they were obtained from heteronuclear single quantum coherence experiments (indicated by "obs"). All spectra are provided in the Supplemental Information.

Isolation of Conglobatin

To obtain conglobatin **2**, 1 l of broth was extracted three times with 200 ml of ethyl acetate. The combined extracts were evaporated to dryness, re-dissolved in 300 ml of methanol, and extracted twice with 100 ml of hexane. The methanol phase was then evaporated and the residue was dissolved in 5 ml of methanol for further purification by preparative HPLC using water and methanol as solvents, both containing 0.1% formic acid (v/v). The sample was eluted using a gradient of 50%–98% methanol over 30 min. Conglobatin-containing fractions were combined and evaporated to remove methanol. The remaining aqueous phase was then extracted three times with 0.5 volume of ethyl acetate, and evaporation of the ethyl acetate yielded 382 mg of conglobatin as a pale yellow solid. The structure of **2** was confirmed by 1D and 2D NMR (for this and other compounds discussed below, spectra and assignments are shown in the Supplemental Information).

Synthesis of Conglobatin Seco Acid 4a

To generate **4a**, 191 mg of conglobatin was hydrolyzed in 200 ml of 1.2 M NaOH containing 40% methanol at 65°C, and stirred vigorously for 2 hr. The reaction mixture was extracted twice with 60 ml of diethyl ether/hexane (3:1, v/v). The aqueous phase was evaporated to remove methanol, the pH was adjusted to 3.0, and the aqueous phase was extracted three times with 60 ml of ethyl acetate to recover **4a**. After evaporation of the ethyl acetate, the residue was dissolved in 5 ml of methanol and **4a** was purified by preparative HPLC using water and acetonitrile as solvents, both containing 0.1% formic acid (v/v). Elution was carried out with a gradient of 30%–66% acetonitrile over 15 min. Fractions containing **4a** were combined and evaporated to remove acetonitrile. The aqueous solution was then extracted three times with 40 ml of ethyl acetate. Evaporation of the ethyl acetate gave 105 mg of **4a** as a pale yellow oil. The structure of **4a** was confirmed by 1D and 2D NMR.

Synthesis of Conglobatin Seco Acid-SNAC 4b

To a solution of **4a** (60 mg), 1-ethyl-3-(3-dimethylaminopropyl)carbodiimide hydrochloride (EDC-HCl, 48 mg) and dimethylaminopyridine (2.5 mg) in CH_2Cl_2 (5 ml) was added 65 μl of *N*-acetylcysteamine, and the mixture was stirred at room temperature for 1.5 hr. The reaction mixture was then diluted with 50 ml of CH_2Cl_2 and washed twice with 10 ml of 0.01 M HCl, then dried under reduced pressure. The crude product was re-dissolved in methanol and purified by preparative HPLC using the same conditions as used for **4a**. Fractions containing **4b** were combined and evaporated to remove acetonitrile. The resulting aqueous phase was then extracted three times with 0.5 volume ethyl acetate, and after removal of the ethyl acetate 40 mg of **4b** was obtained as a pale yellow oil. The structure of **4b** was confirmed by 1D and 2D NMR.

Purification of 2 and 5 from Cong-TE Assay Mixtures

Cong-TE protein was removed from the assay solution by adding 4 volumes of acetonitrile, then centrifuged. The supernatant was evaporated to remove acetonitrile, and the remaining aqueous phase was extracted three times with 0.5 volume of ethyl acetate. The organic phase was evaporated to dryness and dissolved in methanol for purification by preparative HPLC. Water and acetonitrile were used for elution with a gradient of 40%–98% acetonitrile over 30 min. Product-containing fractions were combined and evaporated to remove acetonitrile. The remaining aqueous solution was then extracted three times with 0.5 volume of ethyl acetate. Approximately 0.3 mg of **5** and 0.6 mg of **2** were obtained after removal of the organic extract. The identity of **2** was confirmed by comparison of 1D and 2D NMR data with data obtained for the authentic sample isolated from *S. conglobatus*. The structure of **5** was confirmed by 1D and 2D NMR.

ACCESSION NUMBERS

The sequence of the conglobatin biosynthetic gene cluster of *S. conglobatus* ATCC 31005 and the flanking regions has been submitted to the European Nucleotide Archive under accession number European Nucleotide Archive: LN_849060.

SUPPLEMENTAL INFORMATION

Supplemental Information includes two tables, four figures, and 18 NMR spectra and can be found with this article online at http://dx.doi.org/10.1016/j.chembiol.2015.05.010.

ACKNOWLEDGMENTS

We gratefully acknowledge BBSRC (project grant BB/J007250/1 to P.F.L.), the European Commission (Marie Curie Fellowship to Y.Z.), and the University of Cambridge (Herchel Smith Research Fellowship to A.C.M.), and Ms. Asha Boodhun (Department of Chemistry, University of Cambridge) for help in HR-MS analysis. L.C.D. acknowledges the support of Fundação de Amparo à Pesquisa do Estado de São Paulo (FAPESP, Proc. 2012/04616-3 and 2012/02230-0). P.F.L. is an International Research Awardee of the Alexander von Humboldt Foundation.

Received: March 24, 2015
Revised: May 15, 2015
Accepted: May 17, 2015
Published: June 18, 2015

REFERENCES

Akey, D.L., Kittendorf, J.D., Giraldes, J.W., Fecik, R.A., Sherman, D.H., and Smith, J.L. (2006). Structural basis for macrolactonization by the pikromycin thioesterase. Nat. Chem. Biol. *2*, 537–542.

Bierman, M., Logan, R., O'Brien, K., Seno, E.T., Rao, R.N., and Schoner, B.E. (1992). Plasmid cloning vectors for the conjugal transfer of DNA from *Escherichia coli* to *Streptomyces* spp. Gene *116*, 43–49.

Boddy, C.N., Scheider, T.L., Hotta, K., Walsh, C.T., and Khosla, C. (2003). Epothilone C macrolactonization and hydrolysis are catalyzed by the isolated thioesterase domain of epothilone polyketide synthase. J. Am. Chem. Soc. *125*, 3428–3429.

Bork, P., and Koonin, E.V. (1994). A P-loop-like motif in a widespread ATP pyrophosphatase domain: implications for the evolution of sequence motifs and enzyme activity. Proteins *20*, 347–355.

Caffrey, P. (2003). Conserved amino acid residues correlating with ketoreductase stereospecificity in modular polyketide synthases. ChemBioChem *4*, 649–662.

Dramae, A., Nithithanasilp, S., Choowong, W., Rachtawee, P., Prabpai, S., Kongsaeree, P., and Pittayakhajonwut, P. (2013). Antimalarial 20-membered macrolides from *Streptomyces* sp BCC33756. Tetrahedron *69*, 8205–8208.

Dunbar, K.L., Melby, J.O., and Mitchell, D.A. (2012). YcaO domains utilize ATP to activate amide backbones during peptide cyclodehydrations. Nat. Chem. Biol. *8*, 569–575.

Dunbar, K.L., Chekan, J.R., Cox, C.L., Burkhart, B.J., Nair, S.K., and Mitchell, D.A. (2014). Discovery of a new ATP-binding motif involved in peptidic azoline biosynthesis. Nat. Chem. Biol. *10*, 823–829.

Gaitatzis, N., Silakowski, B., Kunze, B., Nordsiek, G., Blöcker, H., Höfle, G., and Müller, R. (2002). The biosynthesis of the aromatic myxobacterial electron transport inhibitor stigmatellin is directed by a novel type of modular polyketide synthase. J. Biol. Chem. *277*, 13082–13090.

Gehret, J.J., Gu, L., Gerwick, W.H., Wipf, P., Sherman, D.H., and Smith, J.L. (2011). Terminal alkene formation by the thioesterase of curacin A biosynthesis: structure of a decarboxylating thioesterase. J. Biol. Chem. *286*, 14445–14454.

Gibson, D.G., Young, L., Chuang, R.Y., Venter, J.C., Hutchison, C.A., 3rd, and Smith, H.O. (2009). Enzymatic assembly of DNA molecules up to several hundred kilobases. Nat. Methods *6*, 343–345.

Giraldes, J.W., Akey, D.L., Kittendorf, J.D., Sherman, D.H., Smith, J.L., and Fecik, R.A. (2006). Structural and mechanistic insights into polyketide macrolactonization from polyketide-based affinity labels. Nat. Chem. Biol. *2*, 531–536.

Gomez-Escribano, J.P., and Bibb, M.J. (2011). Engineering *Streptomyces coelicolor* for heterologous expression of secondary metabolite gene clusters. Microb. Biotechnol. *4*, 207–215.

Haydock, S.F., Aparicio, J.F., Molnár, I., Schwecke, T., Khaw, L.E., König, A., Marsden, A.F., Galloway, I.S., Staunton, J., and Leadlay, P.F. (1995). Divergent sequence motifs correlated with the substrate specificity of (methyl)malonyl-CoA:acyl carrier protein transacylase domains in modular polyketide synthases. FEBS Lett. *374*, 246–248.

He, J., and Hertweck, C. (2003). Iteration as programmed event during polyketide assembly; molecular analysis of the aureothin biosynthesis gene cluster. Chem. Biol. *10*, 1225–1232.

Henkel, T., and Zeeck, A. (1991). Sekundärstoffe aus dem chemischen Screening, 16. Inthomycine, neue Oxazol-triene aus *Streptomyces* sp. Liebigs Ann. Chem. *1991*, 367–373.

Horsman, M.E., Hari, T.P., and Boddy, C.N. (2015). Polyketide synthase and non-ribosomal peptide synthetase thioesterase selectivity: logic gate or a victim of fate? Nat. Prod. Rep., [Epub ahead of print].

Hoyer, K.M., Mahlert, C., and Marahiel, M.A. (2007). The iterative gramicidin S thioesterase catalyzes peptide ligation and cyclization. Chem. Biol. *14*, 13–22.

Huang, W., Ye, M., Zhang, L.R., Wu, Q.D., Zhang, M., Xu, J.H., and Zheng, W. (2014). FW-04-806 inhibits proliferation and induces apoptosis in human breast cancer cells by binding to N-terminus of Hsp90 and disrupting Hsp90-Cdc37 complex formation. Mol. Cancer *13*, 150–162.

Huang, W., Wu, Q., Zhang, M., Kong, Y., Cao, P., Zheng, W., Xu, J., and Ye, M. (2015). Novel Hsp90 inhibitor FW-04-806 displays potent antitumor effects in HER2-positive breast cancer cells as a single agent or in combination with lapatinib. Cancer Lett. *356*, 862–871.

Keatinge-Clay, A.T. (2007). A tylosin ketoreductase reveals how chirality is determined in polyketides. Chem. Biol. *14*, 898–908.

Kelley, L.A., and Sternberg, M.J.E. (2009). Protein structure prediction on the web: a case study using the Phyre server. Nat. Protoc. *4*, 363–371.

Kieser, T., Bibb, M.J., Buttner, M.J., Chater, K.F., and Hopwood, D.A. (2000). Practical Streptomyces Genetics (John Innes Foundation).

Kwan, D.H., and Leadlay, P.F. (2010). Mutagenesis of a modular polyketide synthase enoylreductase domain reveals insights into catalysis and stereospecificity. ACS Chem. Biol. *59*, 829–838.

Mori, T., Takahashi, K., Kashiwabara, M., Uemura, D., Katayama, C., Iwadare, S., Shizuri, Y., Mitomo, R., Nakano, F., and Matsuzaki, A. (1985). Structure of oxazolomycin, a novel β-lactone antibiotic. Tetrahedron Lett. *26*, 1073–1076.

Müller, S., Rachid, S., Hoffmann, T., Surup, F., Volz, C., Zaburannyi, N., and Müller, R. (2014). Biosynthesis of crocacin involves an unusual hydrolytic release domain showing similarity to condensation domains. Chem. Biol. *21*, 855–865.

Nakagawa, H., Kuratani, M., Goto-Ito, S., Ito, T., Katsura, K., Terada, T., Shirouzu, M., Sekine, S., Shigi, N., and Yokoyama, S. (2013). Crystallographic and mutational studies on the tRNA thiouridine synthetase TtuA. Proteins *81*, 1232–1244.

Olano, C., Wilkinson, B., Moss, S.J., Braña, A.F., Méndez, C., Leadlay, P.F., and Salas, J.A. (2003). Evidence from engineered gene fusions for the repeated use of a module in a modular polyketide synthase. Chem. Commun. *2003*, 2780–2782.

Omura, S., Tanaka, Y., Kanaya, I., Shinose, M., and Takahashi, Y. (1990). Phthoxazolin, a specific inhibitor of cellulose biosynthesis, produced by a strain of *Streptomyces* sp. J. Antibiot. *43*, 1034–1036.

Partida-Martinez, L.P., and Hertweck, C. (2007). A gene cluster encoding rhizoxin biosynthesis in "*Burkholderia rhizoxina*", the bacterial symbiont of the fungus *Rhizopus microsporus*. ChemBioChem *8*, 41–45.

Pinto, A., Wang, M., Horsman, M., and Boddy, C.N. (2012). 6-deoxyerythronolide B synthase thioesterase-catalyzed macrocyclization is highly stereoselective. Org. Lett. *14*, 2278–2281.

Pistorius, D., and Müller, R. (2012). Discovery of the rhizopodin biosynthetic gene cluster in *Stigmatella aurantiaca* Sg a15 by genome mining. ChemBioChem *13*, 416–426.

Reid, R., Piagentini, M., Rodriguez, E., Ashley, G., Viswanathan, N., Carney, J., Santi, D.V., Hutchinson, C.R., and McDaniel, R. (2003). A model of structure and catalysis for ketoreductase domains in modular polyketide synthases. Biochemistry *42*, 72–79.

Robbel, L., Hoyer, K.M., and Marahiel, M.A. (2009). TioS T-TE—a prototypical thioesterase responsible for cyclodimerization of the quinoline- and quinoxaline-type class of chromodepsipeptides. FEBS J. *276*, 1641–1653.

Röttig, M., Medema, M.H., Blin, K., Weber, T., Rausch, C., and Kohlbacher, O. (2011). NRPSpredictor2—a web server for predicting NRPS adenylation domain specificity. Nucleic. Acids Res. *39*, W362–W367.

Roy, R.S., Gehring, A.M., Milne, J.C., Belshaw, P.J., and Walsh, C.T. (1999). Thiazole and oxazole peptides: biosynthesis and molecular machinery. Nat. Prod. Rep. *16*, 249–263.

Sambrook, J., and Russell, D.W. (2001). Molecular Cloning: A Laboratory Manual (Cold Spring Harbor Laboratory Press).

Scaglione, J.B., Akey, D.L., Sullivan, R., Kittendorf, J.D., Rath, C.M., Kim, E.S., Smith, J.L., and Sherman, D.H. (2010). Biochemical and structural characterization of the tautomycetin thioesterase: analysis of a stereoselective polyketide hydrolase. Angew. Chem. Int. Ed. Engl. *49*, 5726–5730.

Schneider, T.L., Shen, B., and Walsh, C.T. (2003). Oxidase domains in epothilone and bleomycin biosynthesis: thiazoline to thiazole oxidation during chain elongation. Biochemistry *42*, 9722–9730.

Schönafinger, G., Schracke, N., Linne, U., and Marahiel, M.A. (2006). Formylation domain: an essential modifying enzyme for the nonribosomal biosynthesis of linear gramicidin. J. Am. Chem. Soc. *128*, 7406–7407.

Schregenberger, C., and Seebach, D. (1984). Synthesis and determination of the absolute configuration of the macrodiolide (+)-conglobatin. Tetrahedron Lett. *25*, 5881–5884.

Schrimsher, J.L., Schenmdel, F.J., Stubbe, J., and Smith, J.M. (1986). Purification and characterization of aminoimidazole ribonucleotide synthetase from *Escherichia coli*. Biochemistry *25*, 4366–4371.

Shaw-Reid, C.A., Kelleher, N.L., Losey, H.C., Gehring, A.M., Berg, C., and Walsh, C.T. (1999). Assembly line enzymology by multimodular nonribosomal peptide synthetases: the thioesterase domain of *E. coli* EntF catalyzes both elongation and cyclolactonization. Chem. Biol. *6*, 385–400.

Shiomi, K., Arai, N., Shinose, M., Takahashi, Y., Yoshida, H., Iwabuchi, J., Tanaka, Y., and Omura, S. (1995). New antibiotics phthoxazolins B, C and D produced by *Streptomyces* sp. KO-7888. J. Antibiot. *48*, 714–719.

Tanaka, Y., Kanaya, I., Shiomi, K., Tanaka, H., and Omura, S. (1993). Phthoxazolin A, a specific inhibitor of cellulose biosynthesis from microbial origin I. J. Antibiot. *46*, 1214–1218.

Tran, L., Tosin, M., Spencer, J.B., Leadlay, P.F., and Weissman, K.J. (2008). Covalent linkage mediates communication between ACP and TE domains in modular polyketide synthases. ChemBioChem *9*, 905–915.

Tsai, S.-C., Lu, H., Cane, D.E., Khosla, C., and Stroud, R.M. (2002). Insights into channel architecture and substrate specificity from crystal structures of two macrocycle-forming thioesterases of modular polyketide synthases. Biochemistry *41*, 12598–12606.

Wang, M., Zhou, H., Wirz, M., Tang, Y., and Boddy, C.N. (2009). A thioesterase from an iterative fungal polyketide synthase shows macrocyclization and cross coupling activity and may play a role in controlling iterative cycling through product offloading. Biochemistry *48*, 6288–6290.

Westley, J.W., Liu, C.M., Evans, R.H., and Blount, J.F. (1979). Conglobatin, a novel macrolide dilactone from *Streptomyces conglobatus* ATCC 31005. J. Antibiot. *32*, 874–877.

Wilkinson, C.J., Hughes-Thomas, Z.A., Martin, C.J., Böhm, I., Mironenko, T., Deacon, M., Wheatcroft, M., Wirtz, G., Staunton, J., and Leadlay, P.F. (2002). Increasing the efficiency of heterologous promoters in actinomycetes. J. Mol. Microbiol. Biotechnol. *4*, 417–426.

Yadav, G., Gokhale, R.S., and Mohanty, D. (2003). Computational approach for prediction of domain organization and substrate specificity of modular polyketide synthases. J. Mol. Biol. *328*, 335–363.

Zhang, P., Zhao, Z., Li, H., Chen, X.L., Deng, Z., Bai, L., and Pang, X. (2015). Production of the antibiotic FR-008/candicidin in *Streptomyces* sp. FR-008 is co-regulated by two regulators, FscRI and FscRIV, from different transcription factor families. Microbiology *161*, 539–552.

Zhao, C., Coughlin, J.M., Ju, J., Zhu, D., Wendt-Pienkowski, E., Zhou, X., Wang, Z., Shen, B., and Deng, Z. (2010). Oxazolomycin biosynthesis in *Streptomyces albus* JA3453 featuring an "acyltransferase-less" type I polyketide synthase that incorporates two distinct extender units. J. Biol. Chem. *285*, 20097–20108.

Zhou, Y., Prediger, P., Dias, L.C., Murphy, A.C., and Leadlay, P.F. (2015). Macrodiolide formation by the thioesterase of a modular polyketide synthase. Angew. Chem. Int. Ed. Engl. *54*, 5232–5235.

Chemistry & Biology, Volume *22*

Supplemental Information

Iterative Mechanism of Macrodiolide Formation

in the Anticancer Compound Conglobatin

Yongjun Zhou, Annabel C. Murphy, Markiyan Samborskyy, Patricia Prediger, Luiz Carlos Dias, and Peter F. Leadlay

1. Tables and Figures

Table S1. Deduced ORF functions in the 41 kbp insert cloned in pYJ24 (related to Figure 3B).

Protein	Size (aa)	Proposed function	Identity/ query cover	Protein Homolog [a]
**CongA**	1210	Loading module	47%, 94%	OzmO, ABS90476.1
**CongB**	856	Module 1a	53% , 99%	OzmQ, ABS90478.1
**CongC**	4476	Module 1b, 2, and 3	47%, 95%	DEBS2, Q03132
**CongD**	2124	Module 4	48%, 87%	DEBS2, Q03132
**CongE**	375	Unknown, N-terminal ATP binding motif	80%, 99%	OzmP, WP_037634315
**R1**	195	LuxR family transcriptional regulator	46%, 98%	FscRI, AAQ82551.1
**1**	487	NADP-specific glutamate dehydrogenase	67%, 90%	P31026.2
**2**	315	Oxidoreductase	46%, 90%	P35320.1
**3**	163	HTH-type transcriptional regulator	32%, 77%	P44558.1
**4**	217	Methyltransferase	28%, 66%	B3QLI9.1
**5**	446	Unknown	no hits	
**6**	350	Unknown	no hits	
**7**	208	HTH-type transcriptional regulator	28%, 80%	P96676.1
**8**	381	L-threonine 3-dehydrogenase	90%, 89%	Q82MN2.1
**9**	400	Glycine acetyltransferase	66%, 94%	P0AB78.1
**10**	332	LysR-family transcriptional regulator	29%, 75%	P20668.3

[a] CongA-E and R1 are given GenBank No. Orf 1 to 10 are given the No. of UniProtKB/Swiss-Prot

Table S2. HR-MS Data of the Compounds Used or Generated in Cong-TE Assay (related to Figure 4).

Products	Formula	calc. m/z	det. m/z	Error (ppm)
**2**	$C_{28}H_{38}N_2O_6H^+$	499.2803	499.2791	-2.29
**4a**	C_{14} H_{21} O_4 N_1H^+	268.1543	268.1533	-4.05
**4b**	$C_{18}H_{28}O_4N_2SH^+$	369.1843.	369.1829	-3.59
**5**	$C_{32}H_{47}N_3O_7SNa^+$	640.3027	640.3007	-3.08
**8**	$C_{31}H_{48}N_2O_7SH^+$	593.3255	593.3256	0.17
**9**	$C_{27}H_{41}NO_7H^+$	492.2956	492.2976	4.06
**10**	$C_{45}H_{67}N_3O_{10}SH^+$	842.4620	842.4644	2.85
**11**	$C_{42}H_{61}N_3O_9SH^+$	784.4201	784.4186	-1.99

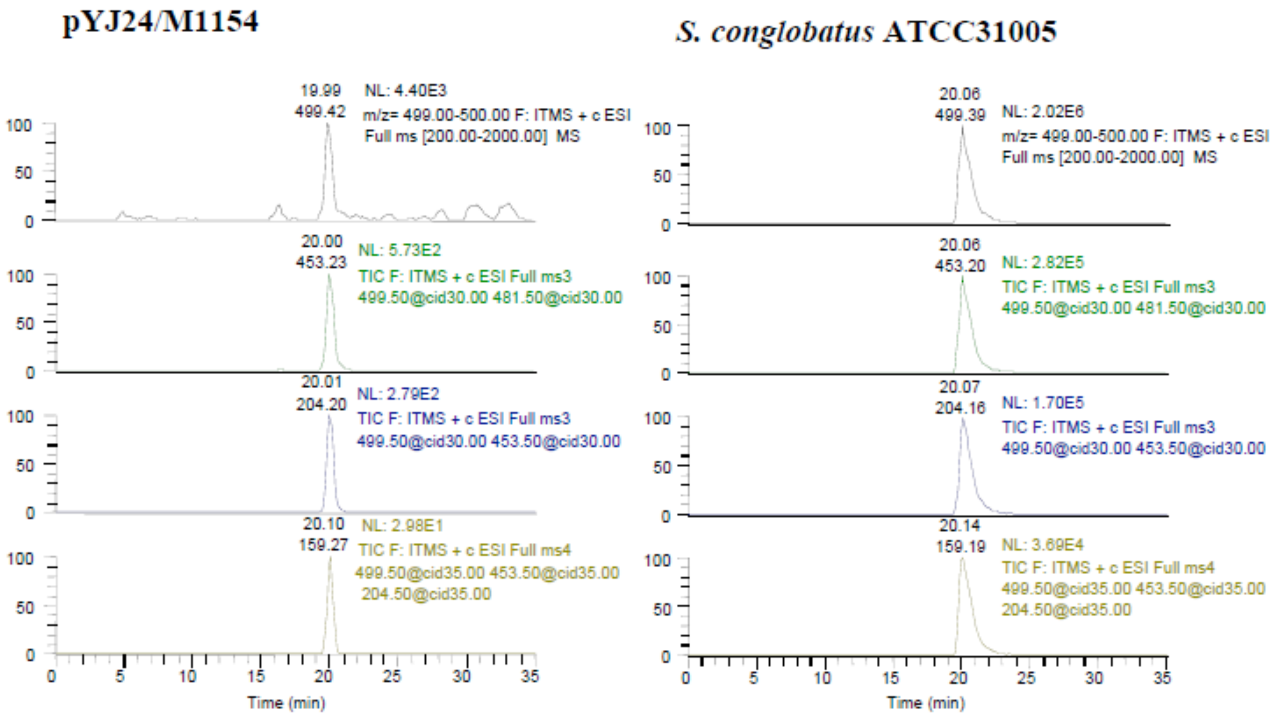

Figure S1. LC-MS/MS analysis of conglobatin heterologous expression in M1154 (related to Figure 3C).

The authentic compound produced by *S. conglobatus* ATCC31005 served as a positive control.

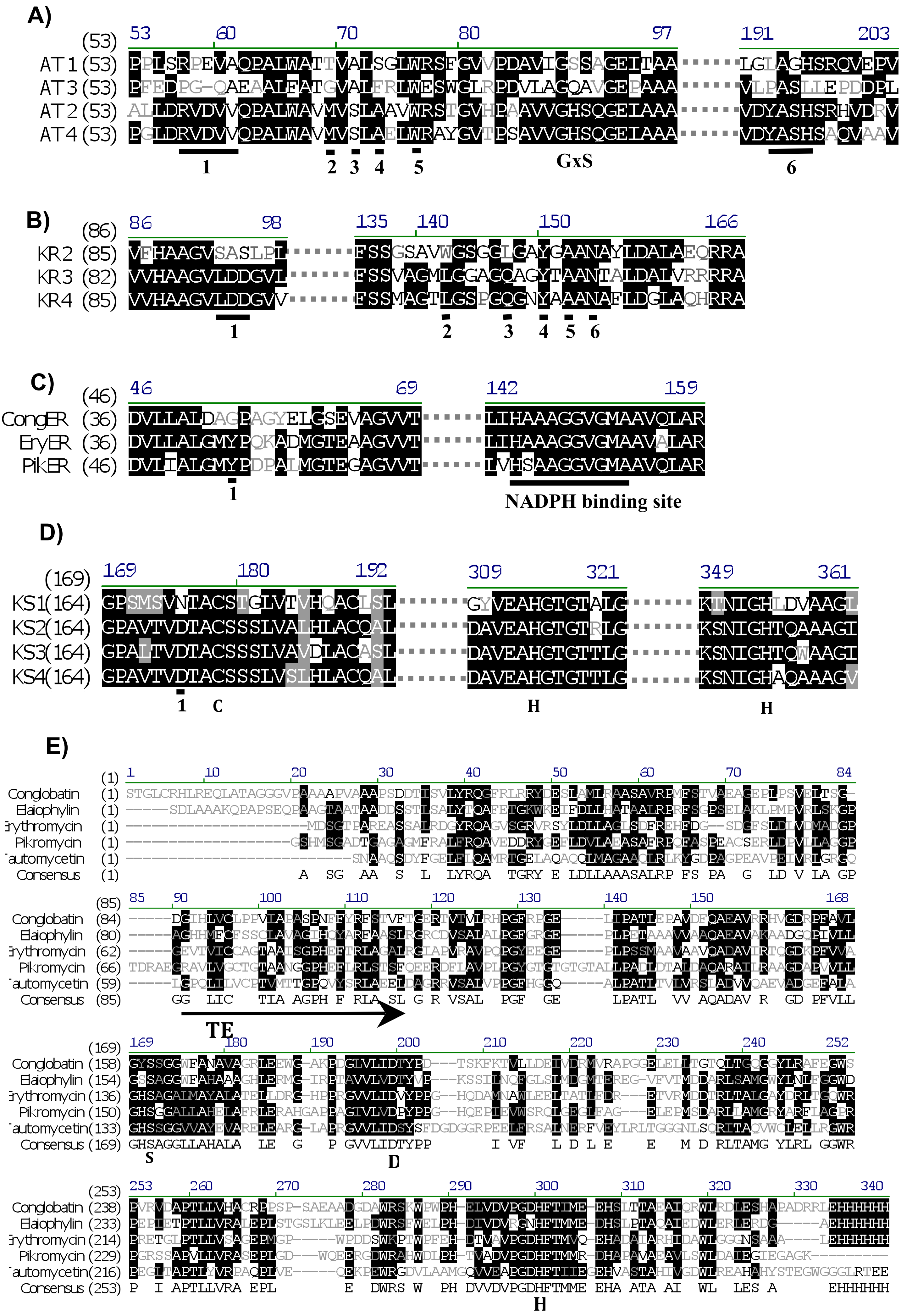
A)
AT1 (53)
AT3 (53)
AT2 (53)
AT4 (53)
1
2
3
4
5
GxS
6
B)
KR2 (85)
KR3 (82)
KR4 (85)
1
2
3
4
5
6
C)
CongER (36)
EryER (36)
PikER (46)
1
NADPH binding site
D)
KS1(164)
KS2(164)
KS3(164)
KS4(164)
1
C
H
H
E)
Conglobatin
Elaiophylin
Erythromycin
Pikromycin
Tautomycetin
Consensus
TE
S
D
H

Figure S2. Amino Acid Sequence Alignment of AT, KR, ER, KS and TE Domains (related to Figure 2). A) Both AT2 and AT4 contain the "YASH" motif specific for loading methylmalonyl unit (Del Vecchio et al., 2003). AT3 has no Ser residue in the "GxS" motif and should be a cryptic domain. The "RVDVV" motif marked by 1 and "MxSxAxxW" motif marked by 2 to 5 are also the footprint of methylmalonyl-CoA specificity. B) KR2 should yield the configuration of *2R* and *3S* according to the residues of position 1: no LDD, 2: W, and 3: no H. KR3 and KR4 should deliver the product with the configuration of *2R* and *3R* to further reduction carried on respectively by DH3 and ER3 in module 3 and DH4 in module 4 according to the residues of position 1: LDD, 5: no P (Keatinge-Clay AT, 2007). C) ER (CongER) from module 3 should generated *2R* configuration rather than *2S* in the case of Y present at 1 position (Kwan *et al.*, 2010). EryER and PikER are ER respectively derived from erythromycin and pikromycin. D) All of the four KS domains have the essential active site cysteine (C) and two conserved histidine (H) residues. KS1 differs from the other three KS domains by the alteration of residue N instead of D in position 1. E) Multiple alignment of the functional TE domains expressed in *E. coli*. Cong-TE was investigated in this work. The TEs respectively derived from erythromycin (Tsai et al., 2001), pikromycin (Giraldes et al., 2006), and tautomycetin (Scaglione *et al.*, 2010) were characterized by crystal structure. The TE from eliophylin was characterized by *in vitro* experiments (Zhou et al., 2015).The start region of the TE domains and the active residues are all labeled.

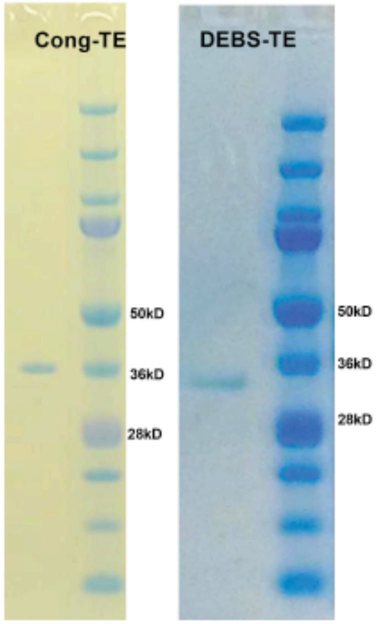

Figure S3. SDS-PAGE gel of the purified proteins (related to Figure 4 and Figure 5).

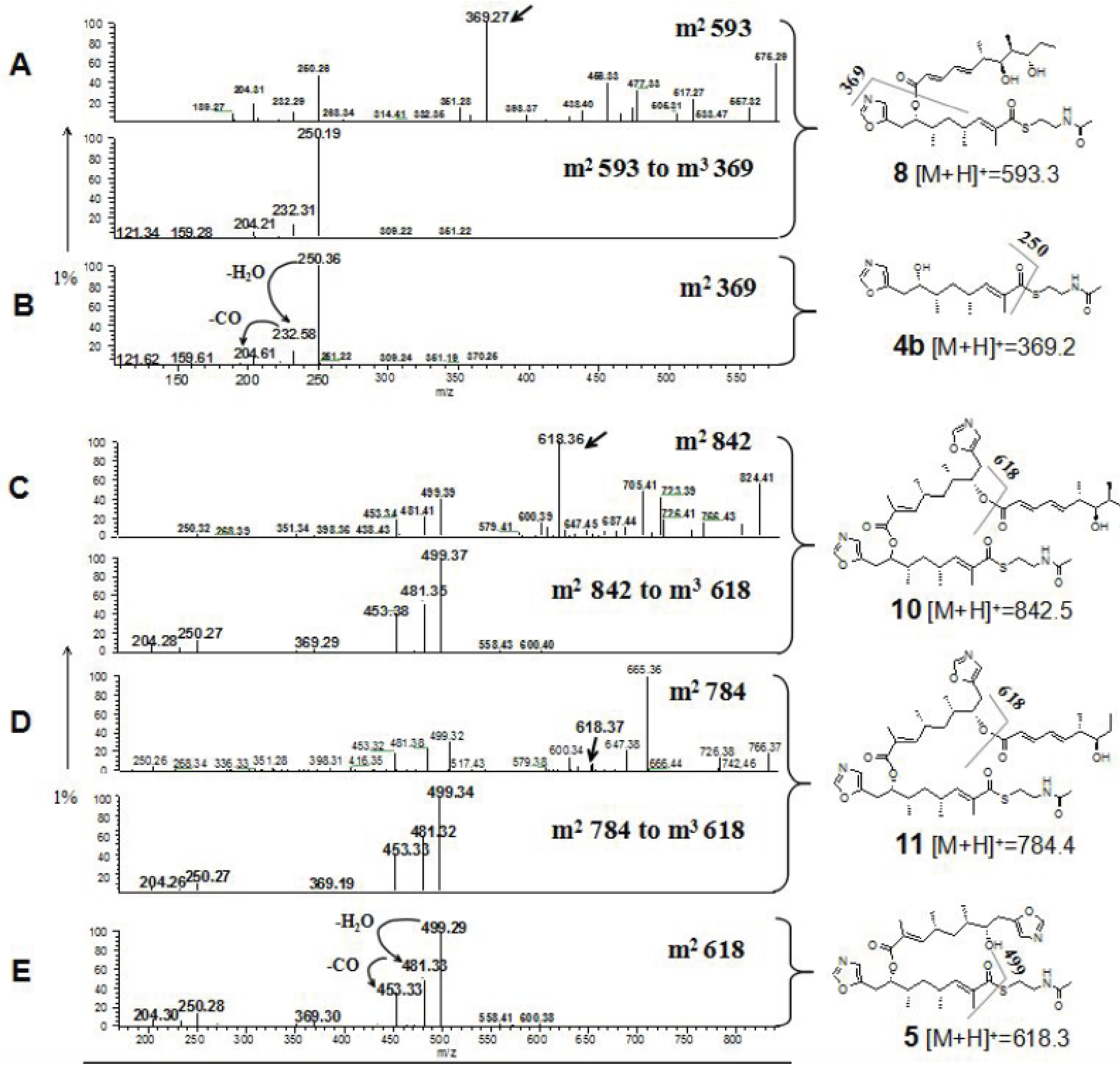

Figure S4. **MS/MS Analysis of Compounds 8, 10, and 11 (related to Figure 5).**

The MS-MS fingerprints of known compounds **4b** and **5** are elucidated respectively in sections B and E. The MS-MS of **4b**, *m/z* 369 (B) is identical to the MS-MS-MS of *m/z* 369 derived from the MS-MS of **8**, *m/z* 593 (A). The MS_2 of **5**, *m/z* 618 (E) is identical to the MS_3 of *m/z* 618 derived from the MS-MS of either **10**, *m/z* 842 (C) or **11**, *m/z* 784 (D). The predicted fragmentations are indicated in the chemical structures.

2. NMR Spectra

2. 1. Compound 2 (produced from fermentation).

^{1}H NMR

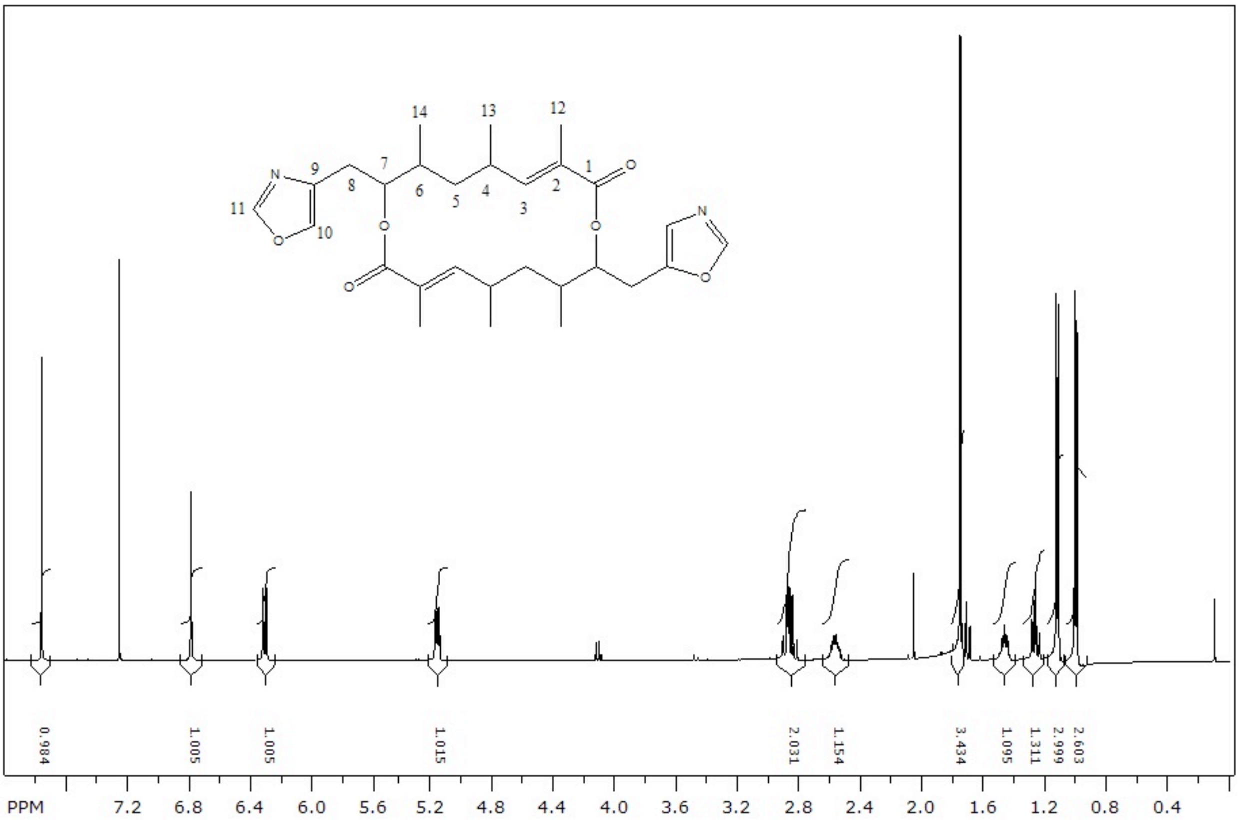

^{13}C NMR

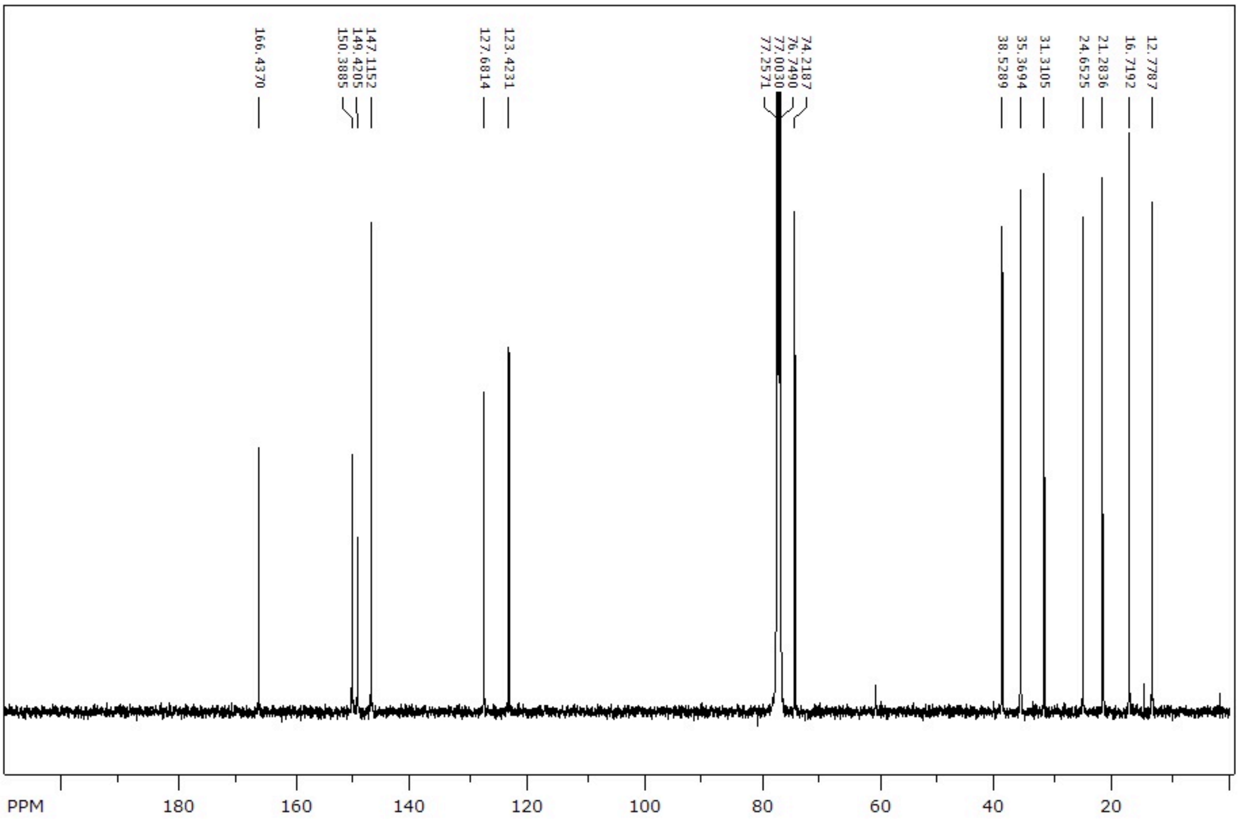

COSY NMR

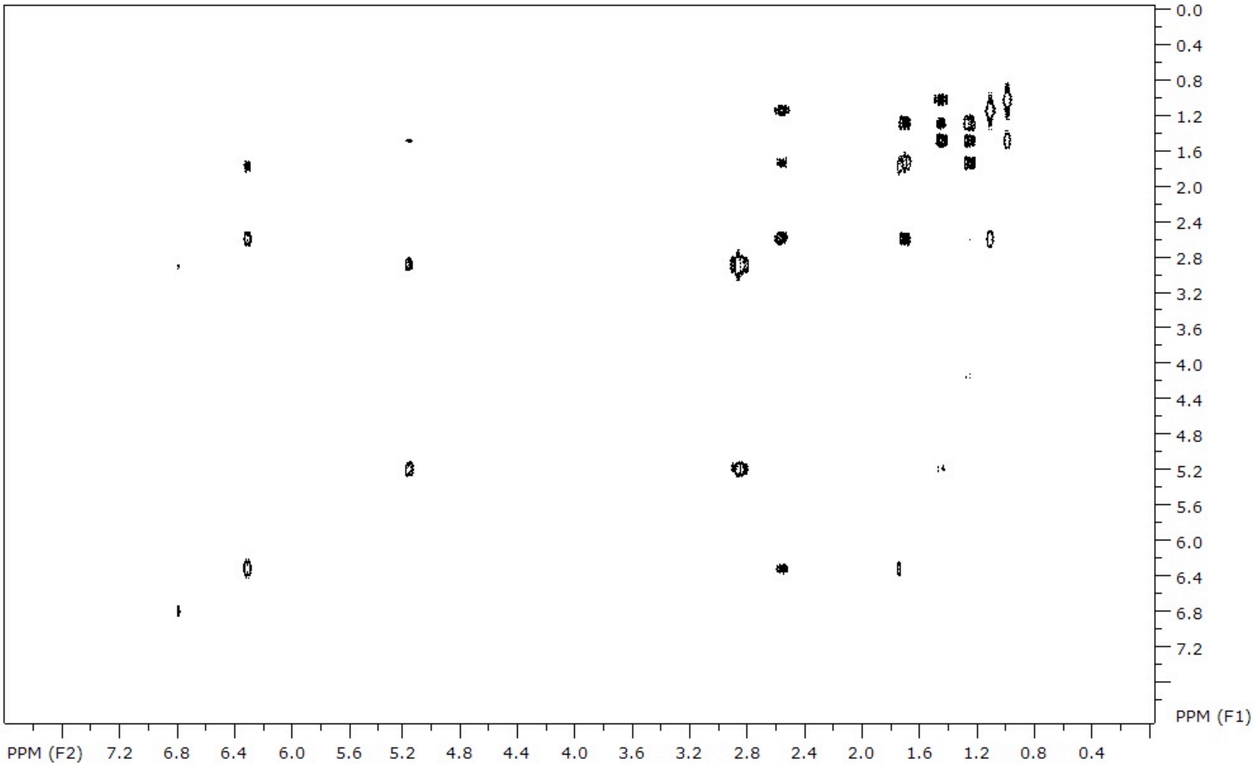

HSQC NMR

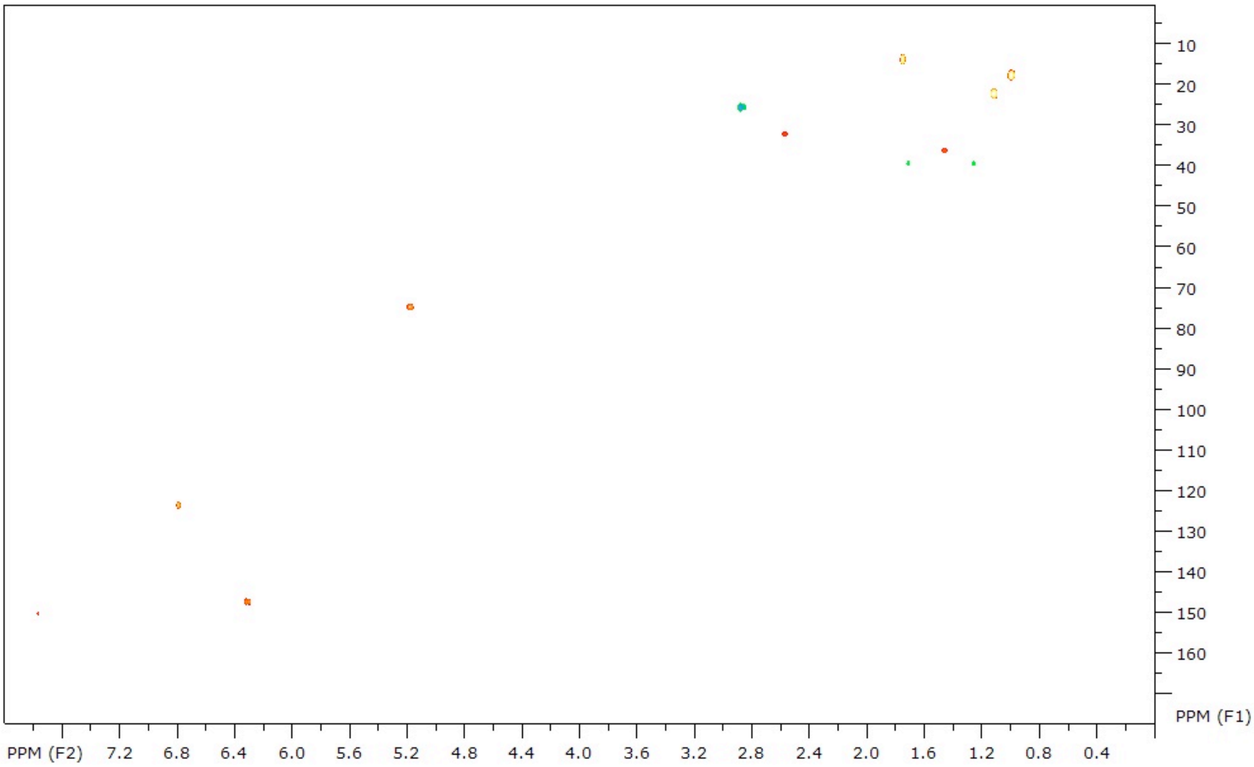

HMBC NMR

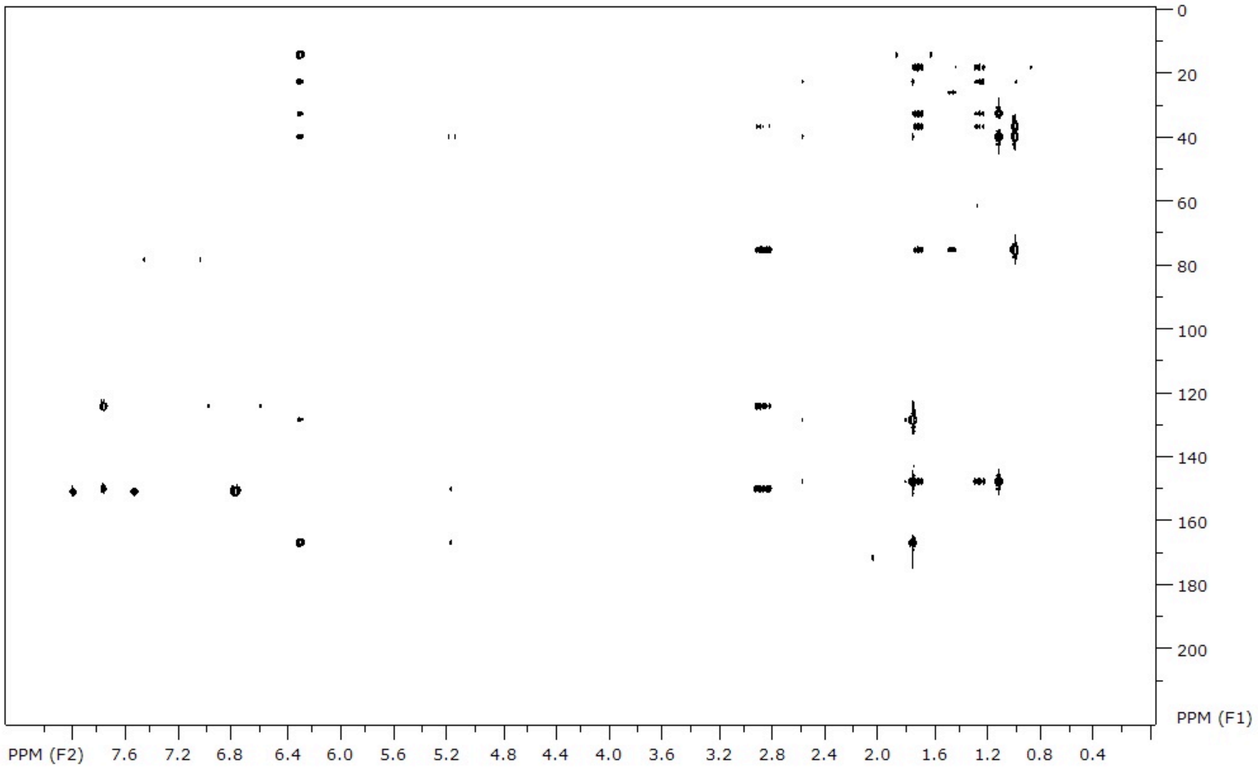

2.2. Compound 4a.

1H NMR

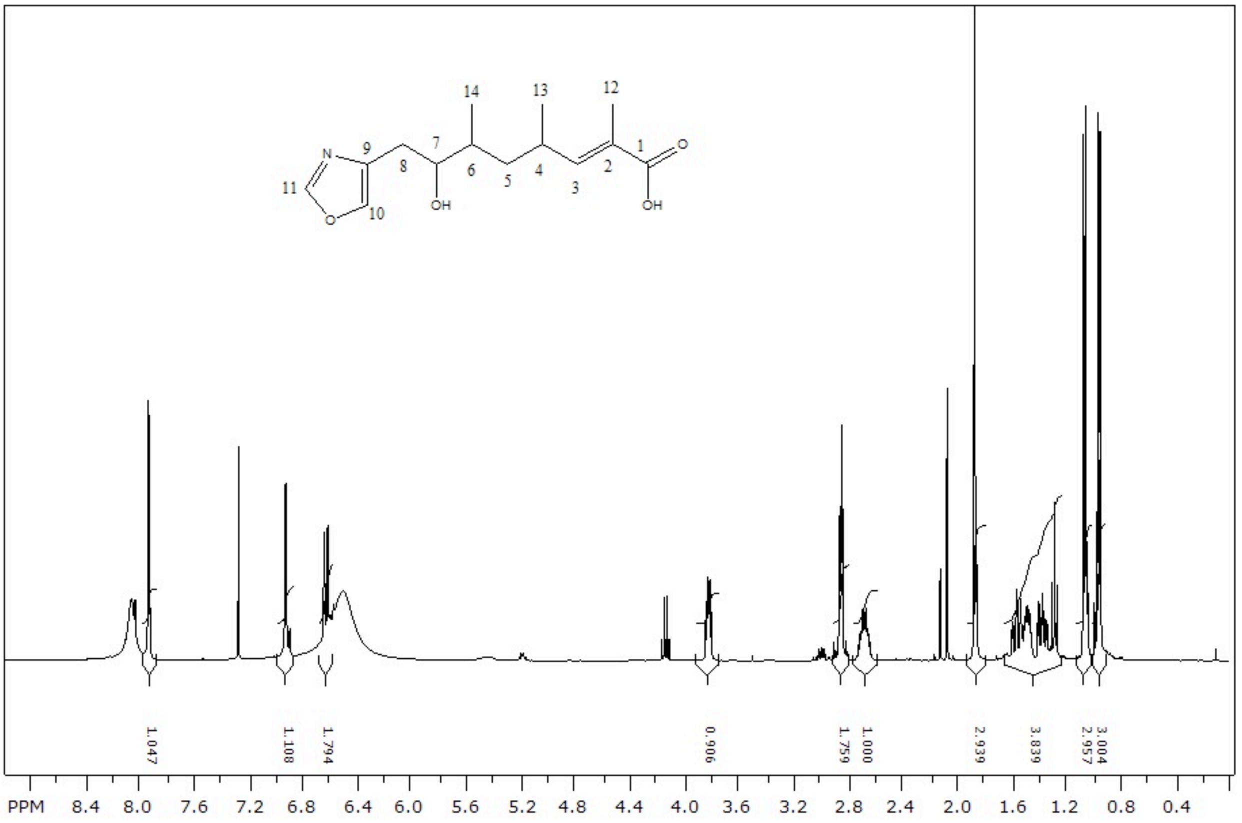

^{13}C NMR

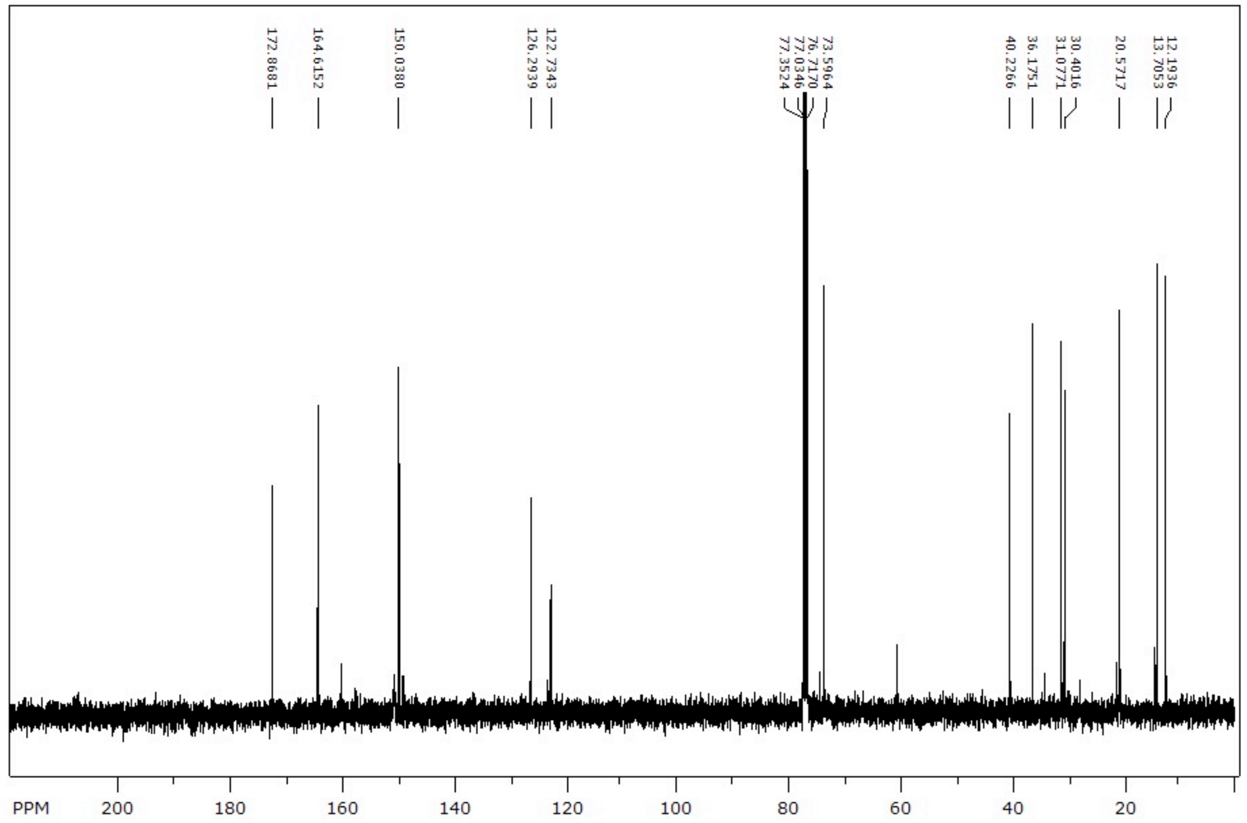

COSY NMR

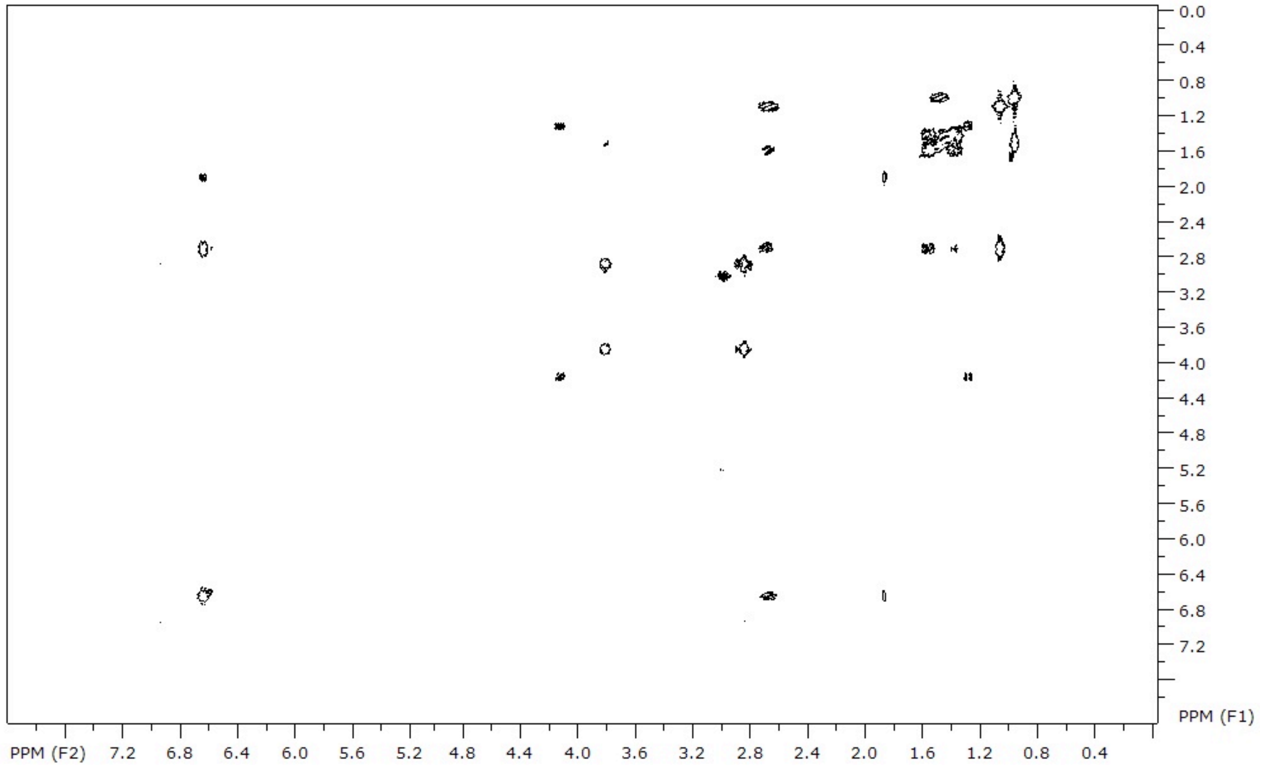

HSQC NMR

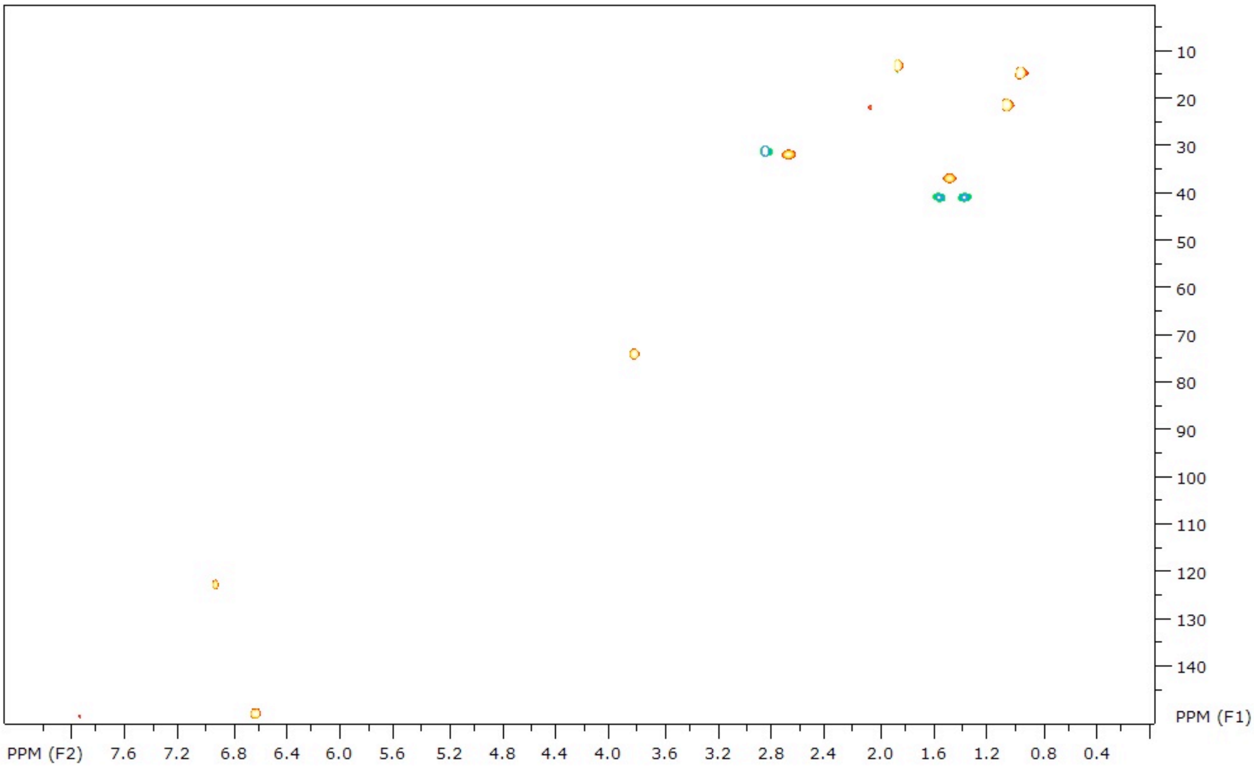

HMBC NMR

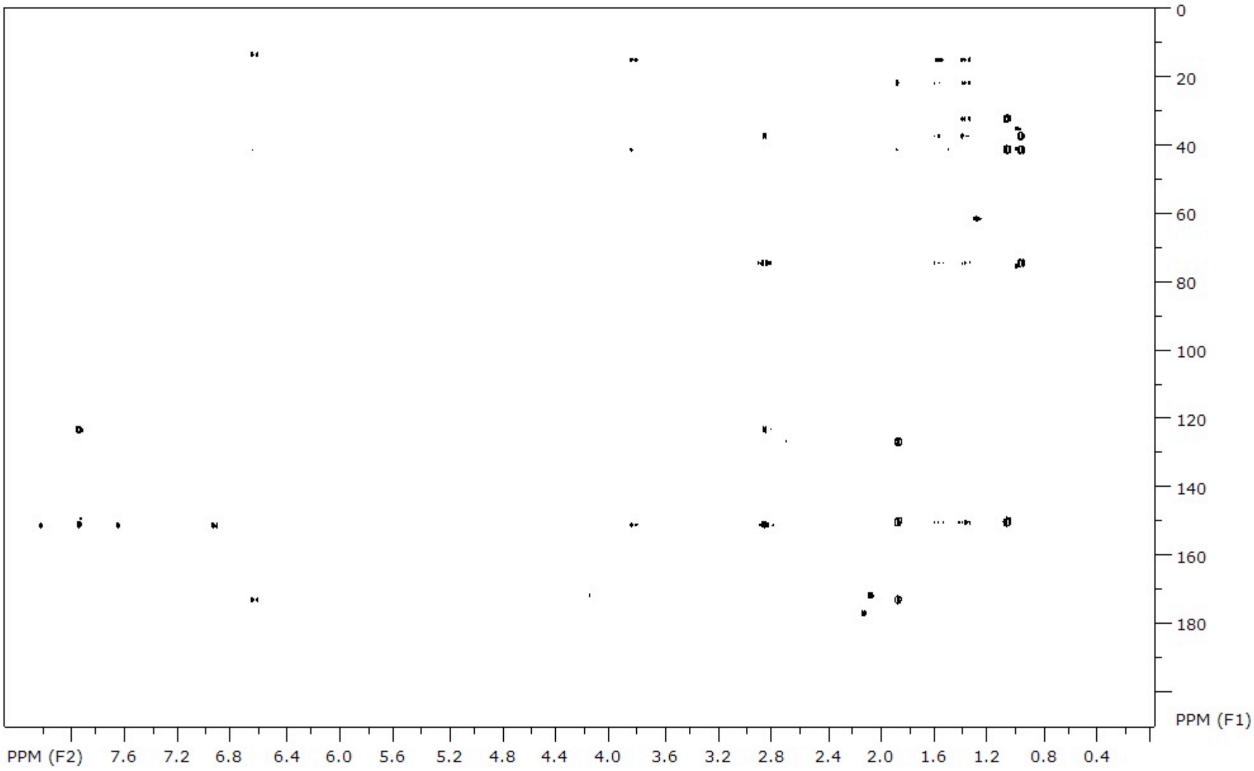

2.3. Compound 4b.

¹H NMR

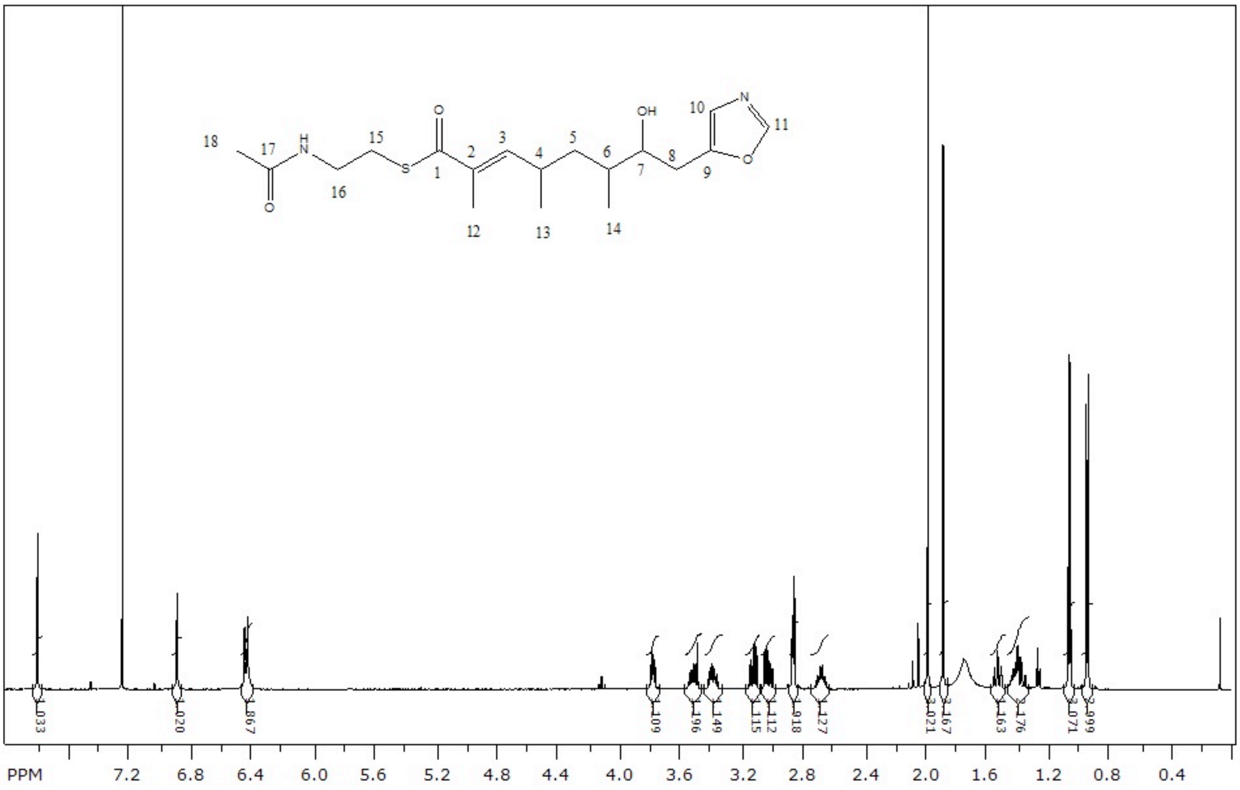

¹³C NMR

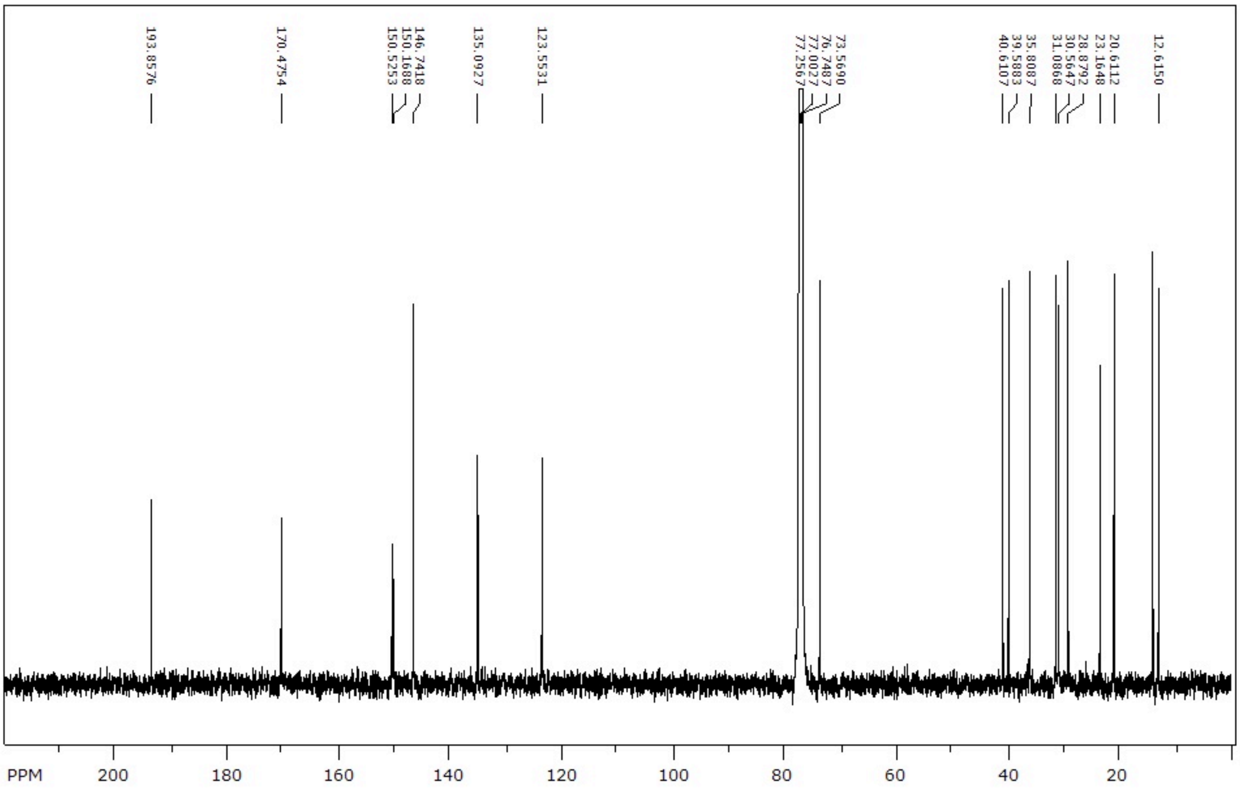

COSY NMR

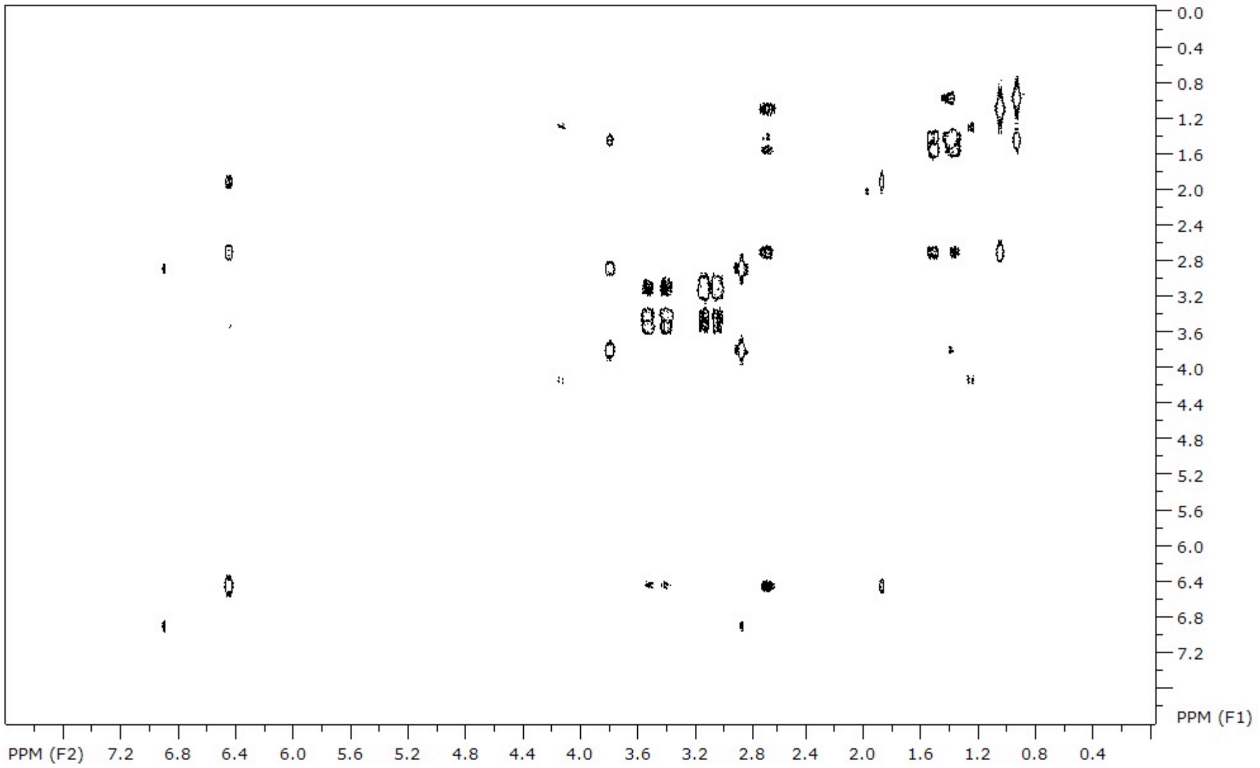

HSQC NMR

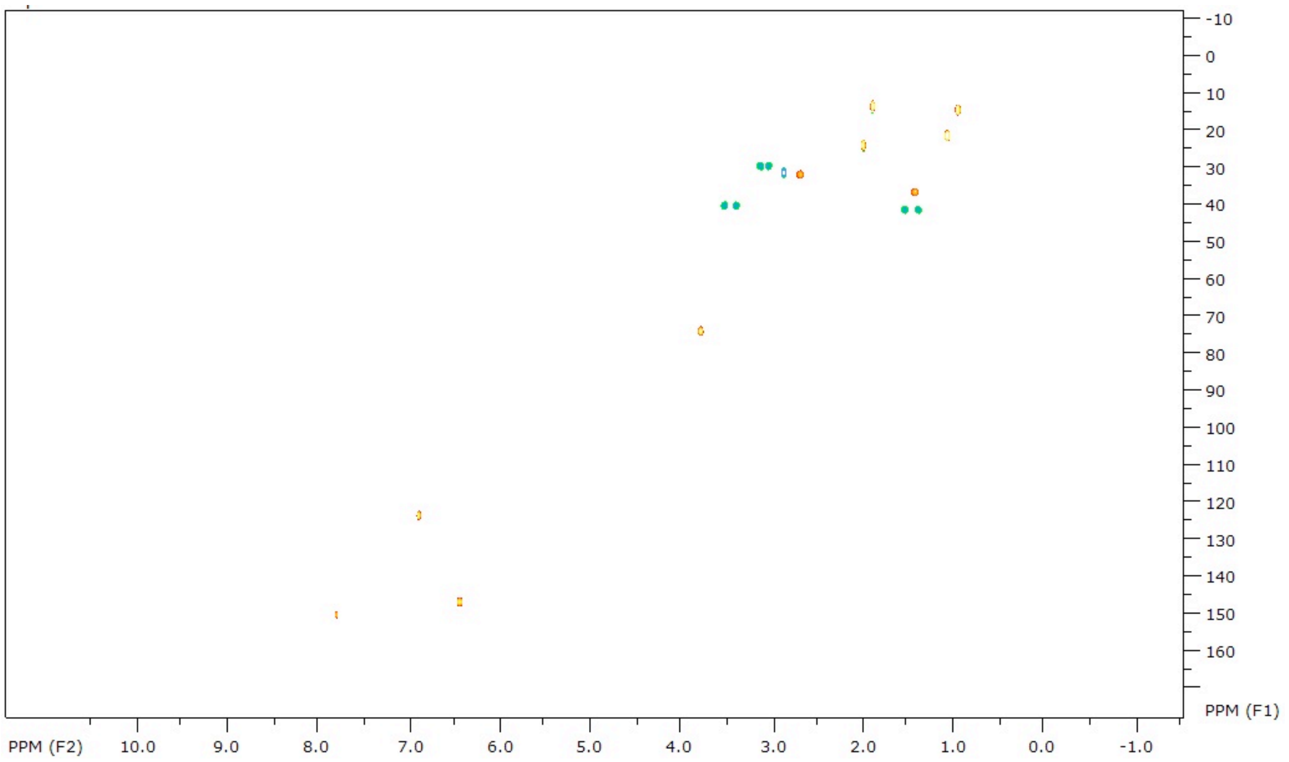

HMBC NMR

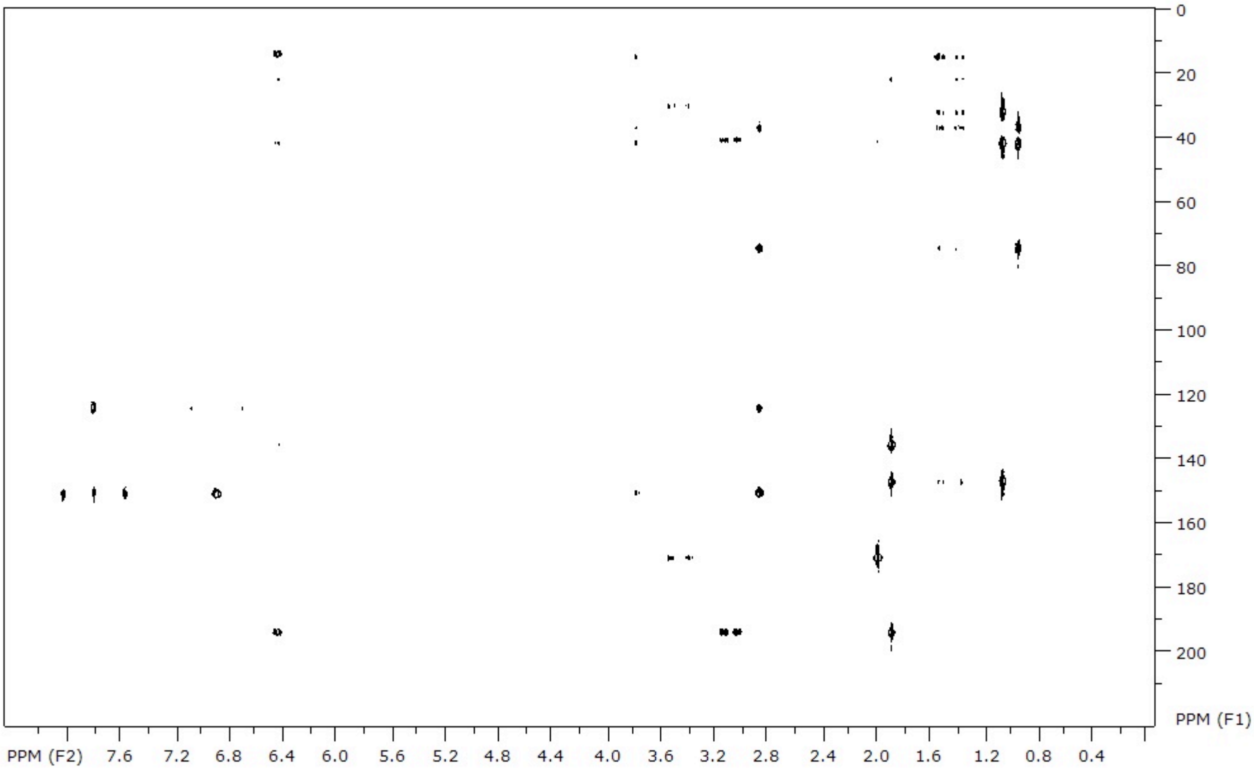

2.4. Compound 5.

^{1}H NMR

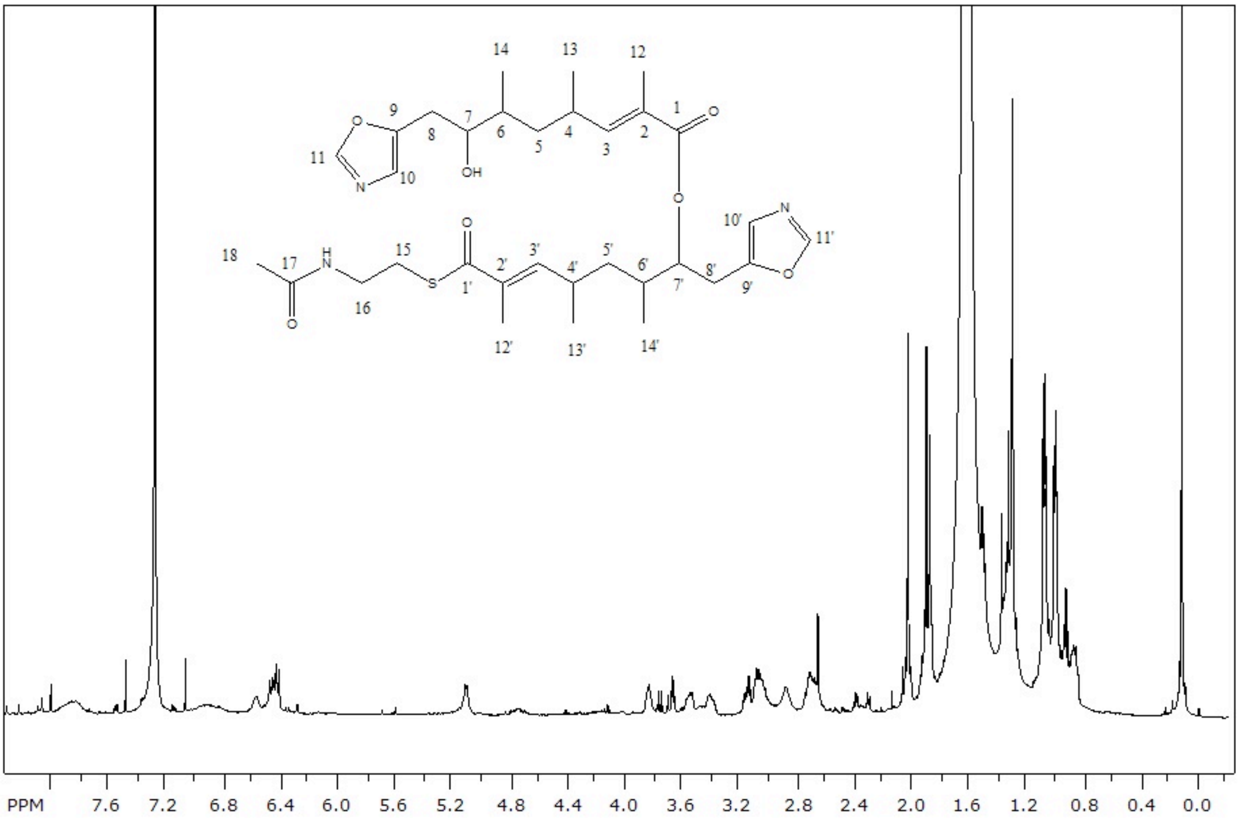

COSY NMR

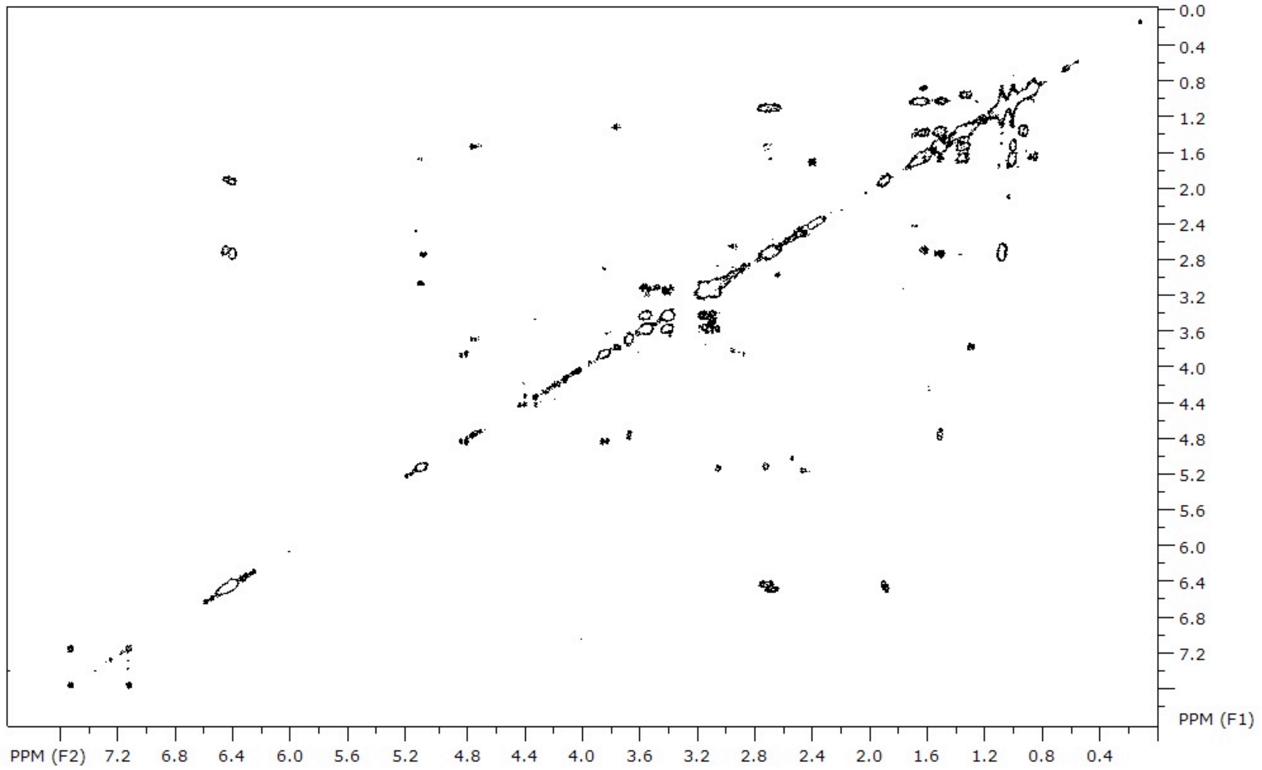

HSQC NMR

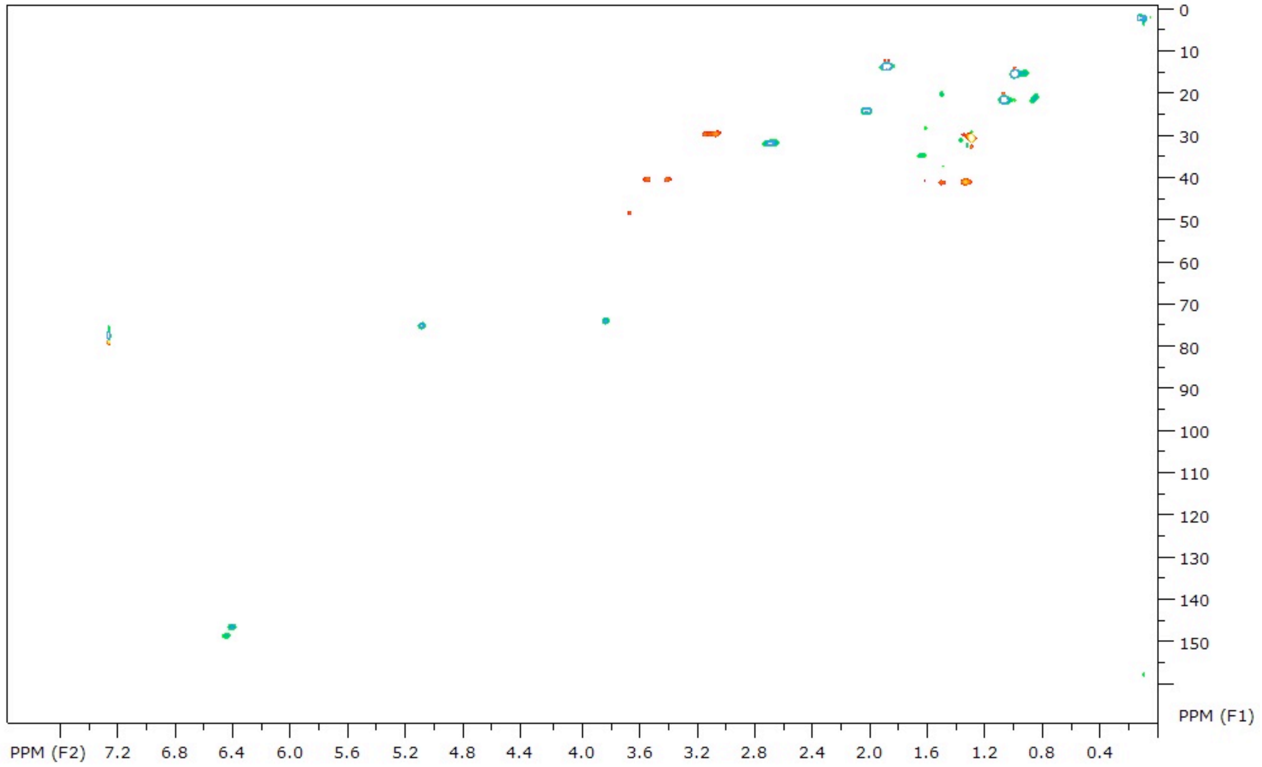

3. Supplemental References

Del Vecchio, F., Petkovic H, Kendrew SG, Low L, Wilkinson B, Lill R, Cortés J, Rudd BA, Staunton J, Leadlay PF. (2003). Active-site residue, domain and module swaps in modular polyketide synthases. J Ind Microbiol Biotechnol. *30,* 489-494.

Giraldes JW, Akey DL, Kittendorf JD, Sherman DH, Smith JL, Fecik RA. (2006). Nat Chem Biol. *2*, 531-536.

Keatinge-Clay AT. (2007). A tylosin ketoreductase reveals how chirality is determined in polyketides. Chem Biol. *14,* 898-908.

Kwan DH, Leadlay PF. (2010). Mutagenesis of a modular polyketide synthase enoylreductase domain reveals insights into catalysis and stereospecificity. ACS Chem Biol. *5*, 829-38.

Scaglione JB, Akey DL, Sullivan R, Kittendorf JD, Rath CM, Kim ES, Smith JL, Sherman DH. (2010). Angew Chem Int Ed Engl. *49*, 5726-5730.

Tsai SC, Miercke LJ, Krucinski J, Gokhale R, Chen JC, Foster PG, Cane DE, Khosla C, Stroud RM. (2001). Proc Natl Acad Sci U S A. *98*, 14808-14813.

Zhou, Y., Prediger, P., Dias, L.C., Murphy, A.C., and Leadlay, P.F. (2015). Macrodiolide formation by the thioesterase of a modular polyketide synthase. Angew. Chem. Int. Ed. Engl. doi: 10.1002/anie.201500401. [Epub ahead of print]
